# The role of spacer length and flexibility in peptide self-assembly

**DOI:** 10.3762/bjoc.22.77

**Published:** 2026-06-25

**Authors:** Julian Link, Albin Lahu, Manfred Wagner, Tanja Weil, David Y W Ng

**Affiliations:** 1 Max Planck Institute for Polymer Research, Ackermannweg 10, D-55128 Mainz, Germany

**Keywords:** nanostructures, peptide amphiphiles, peptide nanofibers, self-assembly, spacer length

## Abstract

Spacer length is a key molecular parameter governing the self-assembly of short peptides. Here, we investigate isoleucine-cysteine-alanine (ICA) tripeptides containing carbon spacers of 6, 3, or 0 methylene units linking the peptide backbone to a hydrophobic naphthalene (Nap) π-block. Using complementary spectroscopic and microscopic techniques, we show that spacer length controls the balance between conformational flexibility and directional non-covalent interactions, thereby dictating assembly pathways and material properties. The results establish a correlation between spacer length and assembly propensity, with the longest spacer (C_6_) consistently promoting aggregation more effectively than the intermediate analogue (C_3_), whereas peptides containing the rigid C_0_-spacer fail to develop ordered nanostructures. These findings identify spacer length as a powerful design parameter for tuning peptide self-assembly across multiple length scales.

## Introduction

Short self-assembling peptides are an eminent class of materials used in supramolecular chemistry due to their advantageous properties such as facile synthesis, programmable molecular information [1–4], structural diversity [5–6], biocompatibility [7–9], and responsiveness to environmental stimuli [10–12]. These peptide monomers can be synthesized in high throughput through solid-phase peptide synthesis (SPPS) and have shown a great structural versatility ranging from the formation of different secondary structures, such as α-helices, β-sheets [13–15], and β-turns [16], to the assembly into distinct supramolecular morphologies, for instance nanosheets [17–18], droplets [19], nanotubes [1,20], nanofibrils [21–22], and coiled coils [23–24]. The information of the secondary structures and supramolecular morphologies are encoded within the sequence, similar to the primary structure of proteins, therefore enabling on-demand production of hierarchical nanostructures. Besides the sequence, the implementation of hydrophobic elements, such as aromatic π-blocks, steroids or alkyl chains, have been successfully applied to enable and modulate the self-assembling properties of short peptides through directional and cooperative non-covalent forces [25–26]. Coupling these to the *N*-terminus of the peptide sequence leads to the formation of peptide amphiphiles with a general architecture of a hydrophobic tail attached to a hydrophilic peptide domain [27–28]. Such peptide amphiphiles have emerged as powerful building blocks to engineer highly-ordered nanostructures in biomedicine [29], nanoscience [30], and cosmetics [31].

While extensive efforts have focused on varying peptide sequences [32] and hydrophobic motifs [33], the role of the linker (or spacer) between these domains has received comparatively little attention. Previous studies indicate that the flexibility and rigidity of the spacer segment strongly influence self-assembly, gelation and bulk mechanical properties of hydrogels [34]. In contrast, studying peptide amphiphiles at lower concentrations, where ordered nanostructures form without macroscopic gelation, is important in elucidating the molecular principles of self-assembly.

**Scheme 1:**
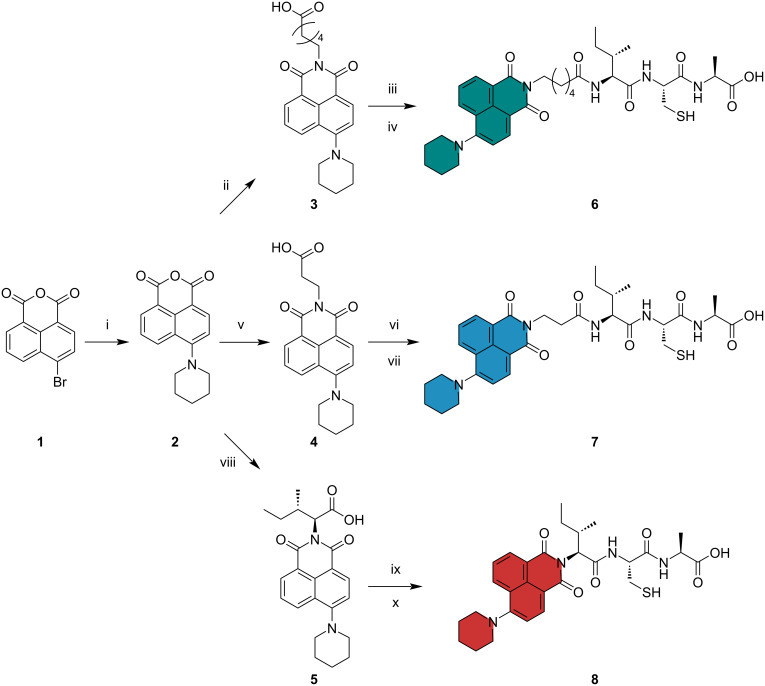
Synthetic scheme for the naphthalene–peptide conjugates C_6_-Nap-ICA **6**, C_3_-Nap-ICA **7**, and C_0_-Nap-ICA **8**. Conditions: i) piperidine, 2-methoxyethanol, 125 °C, overnight; ii) 6-aminohexanoic acid, ethanol, reflux, overnight; iii) H_2_N-Ile-Cys-Ala-Clt-resin, PyBOP^®^, *N*,*N*’-DIPEA, DMF, rt, overnight; iv) TFA (95%), TIPS (2.5%), H_2_O (2.5%), rt, 2 h; v) 3-aminopropanoic acid, ethanol, reflux, overnight; vi) H_2_N-Ile-Cys-Ala-Clt-resin, PyBOP^®^, *N*,*N*’-DIPEA, DMF, rt, overnight; vii) TFA (95%), TIPS (2.5%), H_2_O (2.5%), rt, 2 h; viii) ʟ-isoleucine, ethanol, reflux, overnight, ix) H_2_N-Cys-Ala-Clt-resin, PyBOP^®^, *N*,*N*’-DIPEA, DMF, rt, overnight; x) TFA (95%), TIPS (2.5%), H_2_O (2.5%), rt, 2 h.

To address this gap, we designed a series of peptide amphiphiles with systematically varied spacer lengths. The peptide scaffold consists of the pro-assembling tripeptide Ile-Cys-Ala (ICA), which forms fibrous networks upon functionalization with aromatic groups at the *N*-terminus [35]. To enable both, π–π interactions and fluorescence-based characterization, we chose a naphthalene imide fluorophore as the hydrophobic segment (Nap). The fluorophore was attached either directly to the peptide *N*-terminus (C_0_-spacer), via β-alanine (C_3_-spacer), or 6-aminohexanoic acid (C_6_-spacer), yielding a systematic set of peptide–fluorophore conjugates (Scheme 1). This design allows us to dissect the relationship between spacer length and hierarchical self-assembly. The peptides have been analyzed with respect to their assembly properties using assembly conversion determinations, fluorescence microscopy, transmission electron microscopy (TEM), atomic force microscopy (AFM), circular dichroism (CD) spectroscopy, and nuclear magnetic resonance (NMR) spectroscopy. The results of this work emphasize the importance of π-block flexibility on supramolecular assembly as improved assembly characteristics are observed with increasing spacer length. Therefore, we hypothesize that the spacer length influences assembling properties of amphiphilic peptides directly through the rotational freedom of the π-block, thus enabling or limiting the possible intermolecular interactions. These results help to understand the fundamental role of spacers within peptide amphiphiles and improve the rational design of future peptide assemblies.

## Results and Discussion

### Synthesis

The naphthalene imide spacers were synthesized in two steps starting from 4-bromo-1,8-naphthalic anhydride (**1**) (Scheme 1 and Supporting Information File 1, Scheme S1). First, the bromo substituent in compound **1** was replaced by piperidine in a metal-free Buchwald–Hartwig amination reaction to obtain 6-(piperidin-1-yl)-1*H*,3*H*-benzo[*de*]isochromene-1,3-dione (**2**) with good yield (79%, Figures S1–S6 in Supporting Information File 1) after purification through recrystallization from ethanol [36]. Compound **2** was used as the precursor for the synthesis of the C_6_-/C_3_-, and C_0_-spacer building blocks **3**, **4**, and **5**. To synthesize the C*_x_*-spacers, the anhydride was transformed into the imides by refluxing the corresponding amines with the anhydride in the presence of a catalytic amount of 4-(dimethylamino)pyridine (4-DMAP). Through reaction of **2** with 6-aminohexanoic acid, 6-(1,3-dioxo-6-(piperidin-1-yl)-1*H*-benzo[*de*]isoquinolin-2(3*H*)-yl)hexanoic acid (C_6_-Nap, **3**) was obtained. 3-(1,3-Dioxo-6-(piperidin-1-yl)-1*H*-benzo[*de*]isoquinolin-2(3*H*)-yl)propanoic acid (C_3_-Nap, **4**) was synthesized through reaction of **2** with β-alanine and (2*S*,3*S*)-2-(1,3-dioxo-6-(piperidin-1-yl)-1*H*-benzo[*de*]isoquinolin-2-(3*H*)-yl)-3-methylpentanoic acid (C_0_-Nap, **5**) was obtained from compound **2** and ʟ-isoleucine. The products were washed with 1 M HCl and brine and did not require further purification. The naphthalene imides **3**, **4**, and **5** were obtained with good yields (87–99%) and in high purities (90–95%) as characterized by liquid chromatography–mass spectrometry (LC–MS) and NMR spectroscopy (Supporting Information File 1, Figures S7–S24).

The target tripeptide (ICA) was synthesized in a microwave-assisted peptide synthesizer using the fluorenylmethyloxycarbonyl (Fmoc) solid-phase peptide synthesis strategy [37], synthesizing the peptide from the C- to N-terminus on a chlorotrityl (Clt)-resin according to the general procedure described in the experimental section. To synthesize the C_6_ and C_3_-spacer variants, C_6_-Nap-ICA **6** and C_3_-Nap-ICA **7**, the free amine of H_2_N-Ile-Cys-Ala-Clt-resin was functionalized with the naphthalene imides **3** and **4**, respectively, using (benzotriazol-1-yloxy)tripyrrolidinophosphonium hexafluorophosphate (PyBOP^®^) and *N*,*N’*-diisopropylethylamine (*N*,*N’*-DIPEA) in peptide-grade dimethylformamide (DMF). After the coupling, the peptides were cleaved from the resin under acidic conditions (95% TFA) in the presence of TIPS (2.5%) and H_2_O (2.5%) acting as scavengers to suppress carbocation-mediated side reactions [38]. Furthermore, the utilization of the Clt-resin, instead of the commonly used Wang resin, reduced *S*-alkylation of the thiol side chains [39]. C_0_-Nap-ICA **8** was obtained through coupling of C_0_-Nap **5** to H_2_N-Cys-Ala-Clt-resin using PyBOP^®^ and *N*,*N*’-DIPEA. All peptides were purified via high performance liquid chromatography (HPLC) and subsequently characterized using LC–MS and NMR spectroscopy revealing high purities (>95%) with overall yields between 25–37% (Supporting Information File 1, Figures S25–S33).

### Supramolecular self-assembly

Next, we analyzed the naphthalene-containing peptides **6**, **7**, and **8** regarding their self-assembling properties. Initially, the peptides were dissolved in 2,2,2-trifluoroethanol (TFE) to form a stock solution (10 mM) and added to phosphate-buffered saline (PBS, 50 mM, pH 7.4) to obtain 100 µM final peptide and 1% final TFE concentrations.

By diluting the peptides in neutral aqueous buffer conditions, the hydrophobic intermolecular interactions of the peptide molecules are promoted, thereby inducing self-assembly into supramolecular structures. Here, the π–π interactions of the naphthalene moieties, which are expected to be directly influenced by the flexibility of the spacer length, become the driving force for the self-assembly of the peptide monomers.

**Figure 1:**
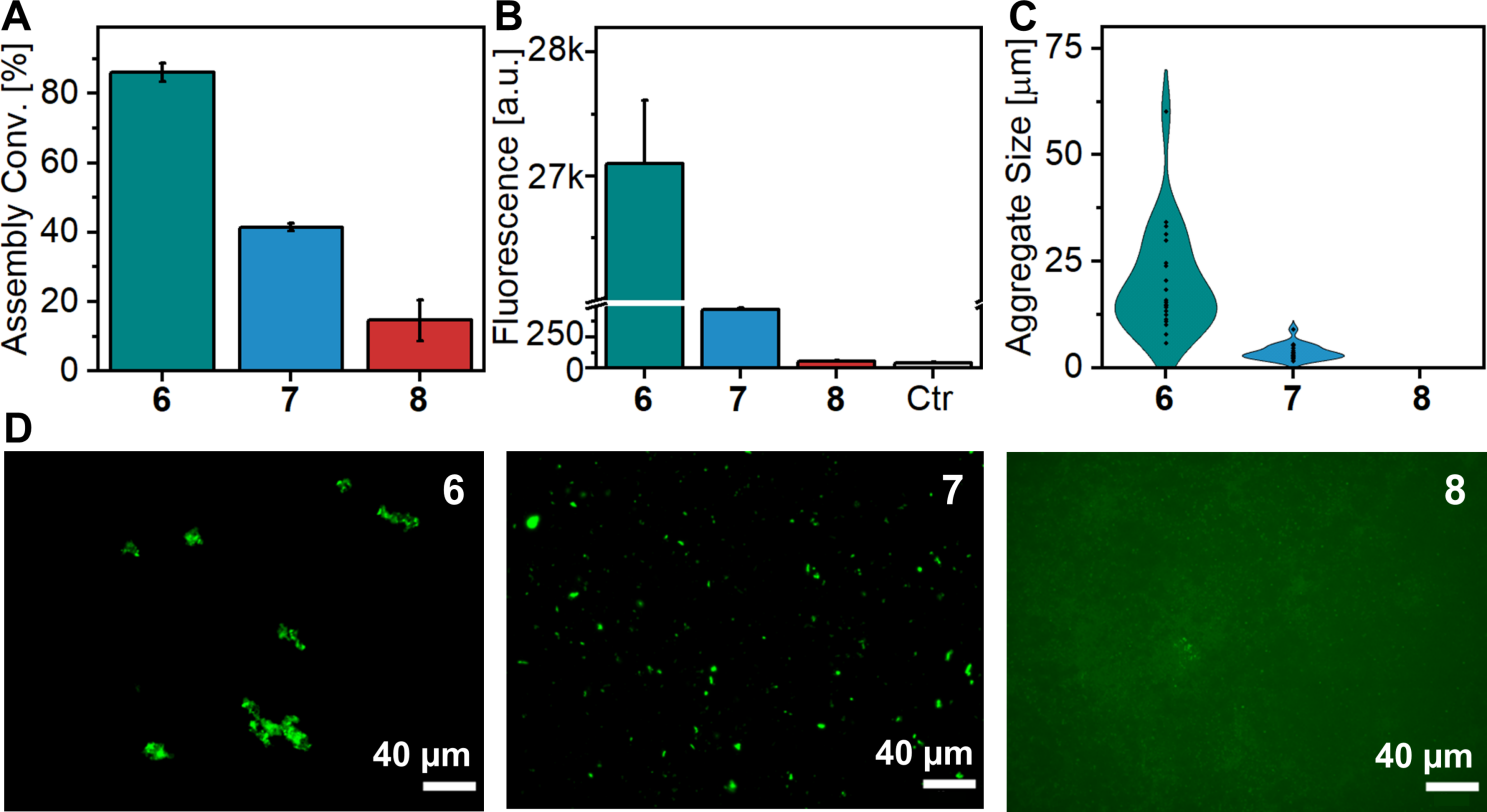
A) Bar plot representing the assembly conversion of C_6_-Nap-ICA **6**, C_3_-Nap-ICA **7**, and C_0_-Nap-ICA **8** (all 100 µM) in PBS (50 mM, pH 7.4, 1% TFE) determined through analytical HPLC. B) Bar plot showing the fluorescence intensity of Proteostat^®^ at λ_em_ = 600 nm (λ_ex_ = 550 nm) after incubation with C_6_-Nap-ICA **6**, C_3_-Nap-ICA **7**, or C_0_-Nap-ICA **8** (all 100 µM) in PBS (50 mM, pH 7.4, 1% TFE). Control shows the fluorescence of Proteostat^®^ in PBS (50 mM, pH 7.4, 1% TFE). C) Violin plot depicting the distribution of assembly sizes for C_6_-Nap-ICA **6**, C_3_-Nap-ICA **7**, and C_0_-Nap-ICA **8** (all at 100 µM) in PBS (50 mM, pH 7.4, 1% TFE) after 24 h incubation at room temperature. The sizes were determined using fluorescence microscopy, *n* = 21. D) Fluorescence microscopy images of C_6_-Nap-ICA **6**, C_3_-Nap-ICA **7**, or C_0_-Nap-ICA **8** (all 100 µM) in PBS (50 mM, pH 7.4, 1% TFE) with green fluorescence filter (λ_ex_ = 470/40 nm, λ_em_ = 525/50 nm), scale bar = 40 µm.

The assembly conversion of peptide monomers to supramolecular structures was analyzed using an analytical HPLC-based study (Figure 1A) [40]. The peptide stock solutions prepared in TFE (10 mM) were diluted in PBS (50 mM, pH 7.4) to obtain 100 µM peptide samples (1% TFE). The mixtures were then incubated at room temperature for 24 h to promote self-assembly. Corresponding samples in methanol (1% TFE) were prepared in parallel and used as molecular, non-assembled controls. After incubation, all samples were filtered (0.2 µm) to separate aggregated peptides from soluble peptide monomers and tyramine (200 µM in MeOH, standard) was added to the filtrates as an internal standard (Figure S34 in Supporting Information File 1). Following, the absorbance of peptide monomers in the filtrates were analyzed, referenced to the molecular controls, to obtain the assembly conversion. In methanol, where no assembly occurs, the retention of the analyzed peptides occurs between 16 and 18 min (Supporting Information File 1, Figure S35). In contrast, lower peptide monomer signals were detected in the PBS-incubated samples as self-assembly removes the peptide monomers from the bulk solution. The assembly conversion rates were calculated based on the integrated absorbance intensities at λ = 214 nm using Equation S1 (Supporting Information File 1) and are presented in Figure 1A as bar plots. Interestingly, a high assembly conversion of the molecular state to the assembled state can only be observed for C_6_-Nap-ICA **6**, (86 ± 3)%. At the tested concentration, C_3_-Nap-ICA **7** assembles at (41 ± 1)%, whereas C_0_-Nap-ICA **8** only assembles minimally, having a low assembly conversion rate of (14 ± 6)%, therefore indicating that intermolecular interactions are weaker for the peptide monomers with short spacer lengths. The obtained results were confirmed by the Proteostat^®^ aggregation assay (Figure 1B). Proteostat^®^ fluorescence increases upon binding to hydrophobic environments present in supramolecular peptide assemblies, particularly β-sheets [41]. Compared to the PBS buffer (50 mM, pH 7.4, 1% TFE) controls, an increase in fluorescence can be observed with increasing spacer length, with the highest fluorescence detected for C_6_-Nap-ICA **6**. Thioflavin T (ThT) assays of the peptides under identical conditions further confirmed the improved self-assembly propensity with increased spacer length, following the same trend as the assembly conversion and Proteostat^®^ aggregation assay (Figure S36 in Supporting Information File 1). The increase in ThT-fluorescence observed for C_6_-Nap-ICA **6** further underlines the presence of β-sheet structures in the peptide fibrils [42–43].

Subsequent analysis of the peptide structures using fluorescence microscopy with a green fluorescence filter (λ_ex_ = 470/40 nm, λ_em_ = 525/50 nm) further confirmed the absence of self-assembled structures for C_0_-Nap-ICA **8** (Figure 1D and Supporting Information File 1, Figure S37). For C_3_- (**7**) and C_6_-Nap-ICA **6**, the formation of supramolecular structures was observed.

Determination of the aggregate sizes revealed large aggregates for C_6_-Nap-ICA **6** at (20 ± 12) µm compared to small aggregates for C_3_-Nap-ICA **7** at (4 ± 2) µm size (Figure 1C and D).

**Figure 2:**
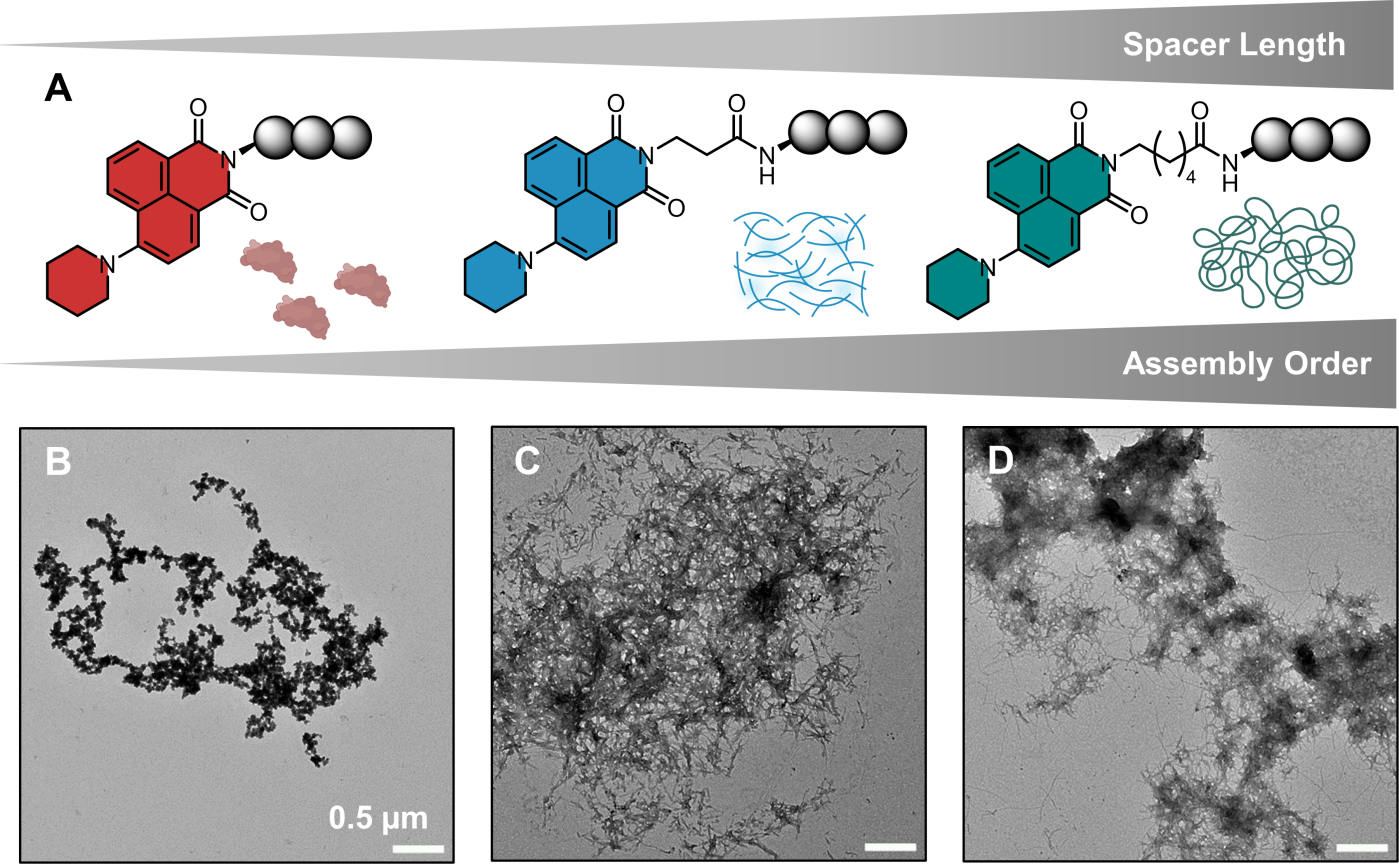
A) Scheme depicting the self-assembly propensities of C_6_-Nap-ICA **6**, C_3_-Nap-ICA **7**, and C_0_-Nap-ICA **8** in aqueous media. B) TEM micrograph of C_0_-Nap-ICA **8** (100 µM) in PBS (50 mM, pH 7.4, 1% TFE) after 24 h incubation at room temperature. C) TEM micrograph of C_3_-Nap-ICA **7** (100 µM) in PBS (50 mM, pH 7.4, 1% TFE) after 24 h incubation at room temperature. D) TEM micrograph of C_6_-Nap-ICA **6** (100 µM) in PBS (50 mM, pH 7.4, 1% TFE) after 24 h incubation at room temperature. The structures were stained with uranyl(IV) acetate (4%), scale bar = 0.5 µm. Created partly in BioRender. Lahu, A. (2026) https://BioRender.com/bvkpq0n. This content is not subject to CC BY 4.0.

Next, concentration-dependent TEM studies were performed to gain deeper insight into the correlation of spacer length and the formation of nanostructures (see Figure 2B–D and Supporting Information File 1, Figure S38). For this purpose, three peptide concentrations were tested, namely 50, 100 and 200 μM in PBS (50 mM, pH 7.4, 1% TFE). Direct attachment of the fluorophore to the peptide backbone (**8**) did not result in ordered structures at any tested concentration: no assemblies were observed at 50 µM, while only small disordered aggregates were formed at 100 and 200 µM. In contrast, both the C_3_- (**7**) and C_6_-spacer (**6**) variants formed well-defined nanofibers. The C_3_-variant **7** exhibited fibers only at 100 and 200 µM, whereas the C_6_-variant **6** displayed fibers even at 50 µM (Supporting Information File 1, Figure S38). To further analyze these fibrillar morphologies, dry-state AFM measurements with tapping mode at 250 kHz were performed (Supporting Information File 1, Figure S39). The peptides were assembled in Milli-Q-H_2_O (1% TFE) with ordered, fibrillar structures observed for C_6_-Nap-ICA **6** with height profiles ranging from 2.4–6.9 nm. C_3_-Nap-ICA **7** also displayed fibrillar structures, however, their vertical distance was observed to be much lower (0.7–0.8 nm). In contrast, C_0_-Nap-ICA **8** showed no supramolecular structures (Supporting Information File 1, Figure S39). Control TEM experiments in Milli-Q-H_2_O (1% TFE) revealed fibrillar structures for peptides **6** and **7** and no supramolecular structures for **8** (Supporting Information File 1, Figure S40). Therefore, the AFM results confirm the results of the observed supramolecular structures by TEM and indicate a strong correlation of spacer length and supramolecular assembly as well as morphology.

Next, CD spectroscopy was used to further analyze the structures of the self-assembling peptides (Supporting Information File 1, Figure S41). The C_0_-spacer variant **8** exhibits minima at 200, 217, and 225 nm. The minimum at 200 nm suggests disordered regions typical of random coils, while the minima at 217 nm and 225 nm correspond to a possible combination of transitions (i.e., n → π* transitions of the peptide backbone in β-sheet secondary structures and aromatic contribution of the naphthalene imide units) [44–47].

The C_3_-spacer variant **7** displays a small positive maximum at 198 nm and similar minima at 217 and 225 nm, but lacks the minimum at 200 nm, suggesting increased order. In the near-UV region, maxima at 280 and 390 nm and a minimum at 330 nm indicate strong aromatic π–π* contributions [48]. Finally, the C_6_-spacer variant **6** displays a far-UV CD spectrum more similar to the C_0_-spacer **8**, with minima at 200, 215, and 230 nm, consistent with a mixture of β-sheet contents, disordered regions, and aromatic contributions. However, in the near-UV, it shows pronounced differences. With intense maxima at 250 nm and a broad feature at 300–350 nm as well as corresponding minima at 280 and 390 nm, the spectrum displays the characteristic bisignate dichroic peaks arising from aromatic exciton coupling [44]. Altogether, the CD spectroscopy results indicate improved aromatic π-block interactions with increasing spacer lengths. These improvements in interactions are expected to cause the observed differences in self-assembly and therefore supramolecular morphologies.

**Figure 3:**
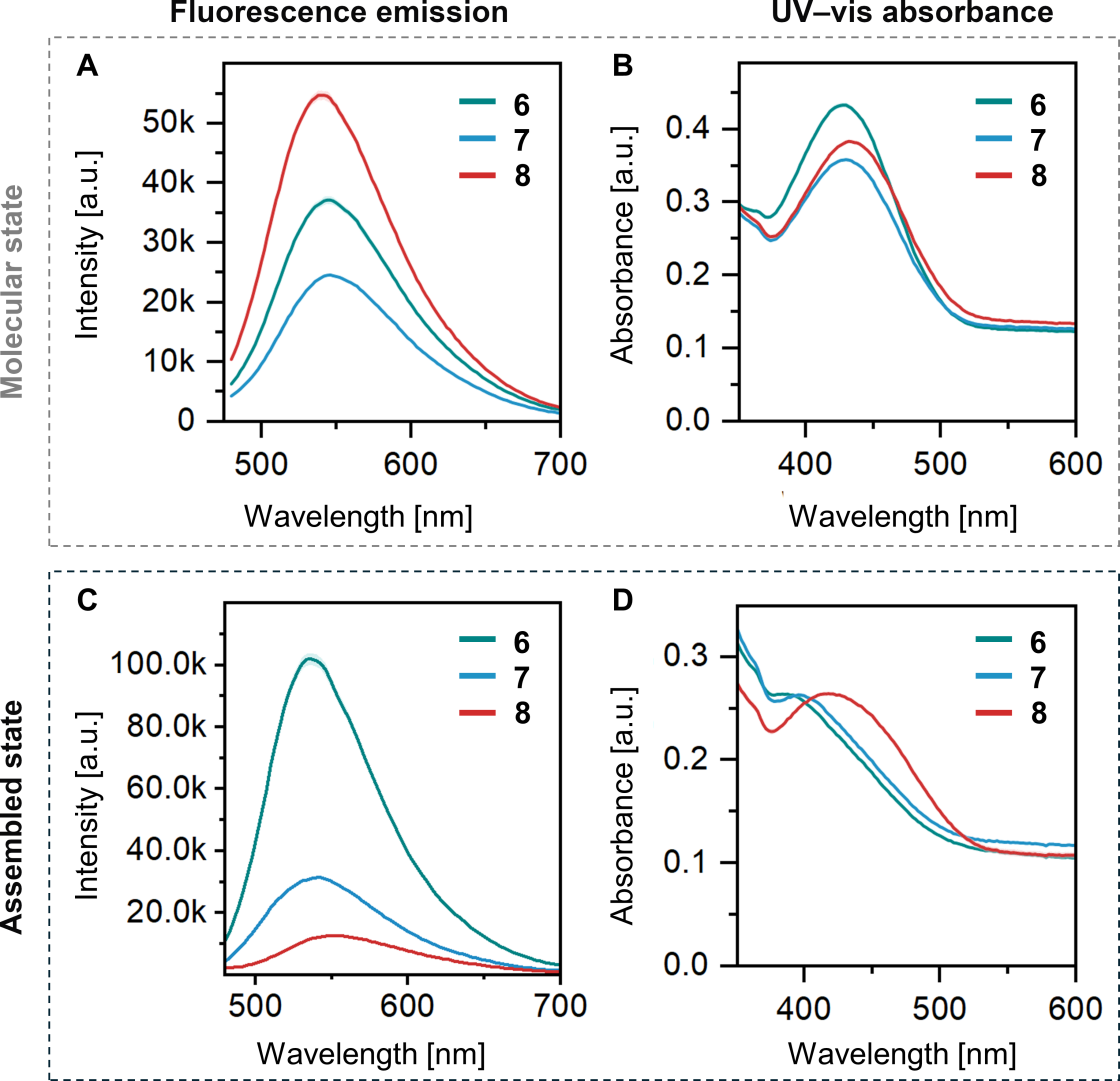
A) Fluorescence emission and B) absorbance of C_6_-Nap-ICA **6**, C_3_-Nap-ICA **7**, and C_0_-Nap-ICA **8** (all 100 µM) in TFE at λ_em_ = 475–700 nm (λ_ex_ = 405 nm) and λ = 350–600 nm. The intensities are plotted against the wavelength. C) Fluorescence emission and D) absorbance of C_6_-Nap-ICA **6**, C_3_-Nap-ICA **7**, and C_0_-Nap-ICA **8** (all 100 µM) in PBS (50 mM, pH 7.4, 1% TFE) at λ_em_ = 475–700 nm (λ_ex_ = 405 nm) and λ = 350–600 nm. The intensities are plotted against the wavelength.

During self-assembly, the attached fluorophores are brought into close proximity, enabling electronic coupling between transition dipoles that can result in characteristic spectral shifts [49]. This behavior is commonly rationalized within the framework of H- and J-aggregates. H-type aggregates typically exhibit a hypsochromic (blue) shift of the absorption maximum [49–50], whereas J-type aggregates display a bathochromic (red) shift [51–52]. To evaluate the influence of spacer length on the spectroscopic properties of the otherwise identical fluorophores, the three peptide conjugates were investigated at 100 μM under non-assembling conditions (100% TFE) and assembling conditions (1% TFE, 99% PBS). In the dissolved state (100% TFE, Figure 3B), all three peptide conjugates exhibited similar absorbance maxima at 430 ± 2 nm.

Upon switching to aqueous assembling conditions, pronounced hypsochromic shifts were observed for C_6_-Nap-ICA (**6**) and C_3_-Nap-ICA (**7**), with absorption maxima at 392 nm (**6**) and 396 nm (**7**), respectively. In contrast, the C_0_-variant **8**, which does not form ordered nanostructures, showed only a minor shift to 426 nm (Figure 3D). The substantial blue shifts observed for compounds **6** and **7** are consistent with excitonic H-type coupling arising from π–π-stacking interactions within ordered assemblies, as also suggested by the CD data (Supporting Information File 1, Figure S41). The magnitude of these shifts suggests the formation of electronically coupled face-to-face chromophore stacks, whereas **8** remains largely monomeric or in a non-ordered state. Analysis of the emission spectra reveals subtle differences already in the dissolved state: the C_0_-spacer-containing compound **8** emits at 540 nm, while the C_3_- (**7**) and C_6_-spacer (**6**) variants both emit at 546 nm, indicating a slightly different microenvironment of the fluorophore in **8** even under non-assembling conditions (Figure 3A). Upon transitioning to the aqueous buffer system, **6** and **7** exhibit mild blue shifts of their emission maximum, whereas **8** shows a red shift (Figure 3C). Additionally, the fluorescence intensities follow the trend C_6_ > C_3_ > C_0_. While ideal H-aggregates are often associated with fluorescence quenching due to exciton splitting and forbidden lowest-energy transitions, the increased emission intensity observed for **6** and **7** suggests deviation from the idealized limit. The enhanced fluorescence likely reflects suppression of non-radiative decay pathways upon self-assembly, potentially due to conformational restriction of the fluorophore, including reduced flexibility of the piperidine unit. In contrast, the red shift observed for **8** under aqueous conditions may arise from changes in solvent polarity surrounding the fluorophore.

Overall, the pronounced hypsochromic absorption shifts for C_6_-Nap-ICA **6** and C_3_-Nap-ICA **7** in assembling conditions support excitonic H-type coupling within ordered supramolecular assemblies. These findings are consistent with the TEM, AFM, Proteostat^®^, and ThT aggregation assay results, which demonstrate more pronounced and ordered nanostructure formation for C_6_-Nap-ICA **6** and C_3_-Nap-ICA **7**. The obtained results from all aforementioned analysis tools are summarized in Table 1.

**Table 1:** Summary of the assembly characteristics of C_6_-Nap-ICA **6**, C_3_-Nap-ICA **7**, and C_0_-Nap-ICA **8** at 100 µM in PBS (50 mM, pH 7.4, 1% TFE).

Peptide	Assembly conversion^a^ [%]	Aggregate sizes^b^ [µm]	Morphology^c^	Secondary structure^d^	Absorbance/emission maximum^e^ [nm]

**6** (C_6_)	86 ± 3	20 ± 12	fibrils	β-sheet (high)	392/537
**7** (C_3_)	41 ± 1	4 ± 2	fibrils	β -sheet (low)	396/543
**8** (C_0_)	14 ± 6	–	amorphous	–	426/550

^a^Determined using the assembly conversion assay at λ = 214 nm by analytical HPLC; ^b^determined using fluorescence microscopy with a green fluorescence filter (λ_ex_ = 470/40 nm, λ_em_ = 525/50 nm), *n* = 21; ^c^determined via TEM; ^d^determined using Proteostat^®^ (λ_ex_ = 550 nm, λ_em_ = 600 nm) and ThT (λ_ex_ = 450 nm, λ_em_ = 482 nm) aggregation assays on a Tecan Spark 20M multi-plate reader in the aggregated state; ^e^determined through absorbance and fluorescence (λ_ex_ = 405 nm) scans on a Tecan Spark 20M multi-plate reader in the aggregated state.

**Figure 4:**
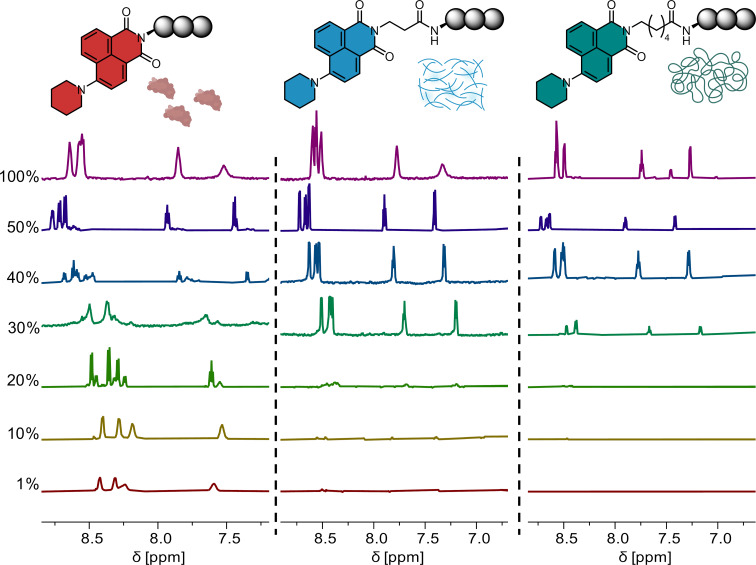
^1^H NMR spectroscopy data (700 MHz, 298 K, mixtures of TFE-*d*_3_ and D_2_O) of C_0_-Nap-ICA **8** (left), C_3_-Nap-ICA **7** (middle), and C_6_-Nap-ICA **6** (right) with increasing TFE-*d*_3_ content. Spectra were normalized to internal standard of dimethyl sulfone. Here, the aromatic region of the naphthalene signals is shown for comparison between the analytes. Full spectra are available in Supporting Information File 1, Figures S42–S62. Created partly in BioRender. Lahu, A. (2026) https://BioRender.com/bvkpq0n. This content is not subject to CC BY 4.0.

^1^H NMR spectroscopy provides valuable insight into the self-assembly behavior of molecules by monitoring spectral changes upon increasing the fraction of an organic co-solvent. A gradual, linear change in signal intensity or chemical shift with increasing co-solvent content is indicative of simple dissolution and bulk solvent effects. In contrast, a pronounced transition from broad, poorly resolved resonances arising from large, slowly tumbling aggregated species to sharp, well-resolved signals characteristic of smaller, faster-tumbling species points to a cooperative self-assembly process [53–56]. To probe this behavior, the three peptide conjugates were dissolved in mixtures of trifluoroethanol-*d*_3_ (TFE-*d*_3_) and PBS-buffered D_2_O with increasing TFE-*d*_3_ content (1:99, 10:90, 20:80, 30:70, 40:60, 50:50, and 100:0, v/v) and analyzed by ^1^H NMR spectroscopy (Figure 4).

At 1% TFE-*d*_3_ content, C_0_-Nap-ICA **8** exhibits detectable resonances, even though line broadening effects are noticeable. This observation is consistent with a largely monomeric state in aqueous medium with a small population of aggregates contributing to slower molecular tumbling and thus line broadening. In contrast, the peptide conjugates with longer spacer lengths show no observable NMR signals at this co-solvent composition due to the formation of substantially larger aggregates that undergo rapid transverse relaxation. The pronounced aggregation of these conjugates is further evidenced by inspection of the corresponding NMR tubes, where clear solid–liquid phase separation was observed (Supporting Information File 1, Figure S63).

Upon increasing the TFE-*d*_3_ content to 20%, the resonances of the C_0_-spacer conjugate **8** sharpen and well-defined multiplicities become visible as the aggregates are dissolved into monomeric states. At this composition, the first signals appear for the C_3_-spacer **7** derivative, whereas no resonances are yet observed for the C_6_-spacer-containing peptide **6**. The requirement of a higher TFE-*d*_3_ fraction for visible NMR signals indicates higher intrinsic aggregation propensity for the C_6_-spacer in comparison to the C_3_-spacer. Further increasing the TFE-*d*_3_ content to 30% results in the emergence of sharp, well-resolved signals for both peptides **6** and **7**. This pronounced transition suggests that the aggregates present in aqueous media are disrupted once a critical level of organic co-solvent is reached. The additional increase in TFE-*d*_3_ content to 50% does not produce further qualitative changes in the spectra, indicating that the systems remain in a predominantly solvated, molecular regime. In summary, this ^1^H NMR spectroscopy study demonstrates that the self-assembly propensity of the peptide conjugates increases with spacer length. These results highlight the importance of flexibility within the linker segment for enabling productive intermolecular interactions that promote the formation of ordered self-assembled structures.

## Conclusion

In this work, we investigated how the spacer segment connecting a short self-assembling peptide core to an *N*-terminal π-block modification influences the resulting self-assembly behavior. Although previous studies have extensively varied both peptide sequences and assembly-promoting π-blocks, the role of the spacer region has received comparatively little attention. By analyzing a systematic series of structurally related peptide conjugates with increasing spacer lengths, we demonstrated that flexibility within this segment is essential for enabling cooperative intermolecular interactions and efficient self-assembly.

Peptide conjugates bearing the most rigid spacer (C_0_-Nap-ICA **8**) failed to form ordered nanostructures, whereas constructs with longer aliphatic spacers (C_3_-Nap-ICA **7** and C_6_-Nap-ICA **6**) assembled readily into well-defined supramolecular architectures. Moreover, spectroscopic and biochemical analyses reveal a clear correlation between spacer length and assembly propensity. Circular dichroism spectroscopy, NMR spectroscopy, and aggregation assays consistently show that the incorporation of a C_6_-spacer promotes self-assembly more effectively than a C_3_-spacer. These results establish the spacer segment as a decisive structural element in the design of short amphiphilic peptides.

The present study focused on assemblies primarily driven by hydrophobic interactions of the π-block and supported by peptide–peptide interactions. In addition, the investigated spacers were limited to relatively short, hydrophobic aliphatic chains. Future work could expand these design principles by exploring longer peptide sequences as well as a broader range of spacer lengths and compositions, including more polar and synthetic linkers such as PEG-based segments. Such studies will further refine the understanding of spacer-controlled peptide self-assembly and provide additional strategies for the rational development of responsive peptide materials.

## Experimental

### Microwave-assisted peptide synthesis

Peptides were synthesized using the Fmoc-SPPS strategy [37], synthesizing the peptide from the C- to N-terminus in a microwave-assisted peptide synthesizer on a Fmoc-ʟ-Ala-Clt-resin at scales of 0.10 mmol. Fmoc-ʟ-Ala-Clt-resin (0.10 mmol) was swollen in DMF (15 mL) at room temperature for one hour before transferring it into the peptide synthesizer. In the peptide synthesizer, DMF was removed through the draining process and DMF (3.5 mL) was added prior to the coupling. Prior to every coupling, the Fmoc-protecting group was cleaved by one deprotection step using a solution of 8% piperidine (DMF, 2.1 mL) for 80 s at 110 °C. The amino acids (0.5 M in DMF, 1.0 mL), DIC (0.75 M in DMF, 1.0 mL) and Oxyma Pure (0.26 M in DMF, 1.5 mL) were added to the resin and the solution was heated to 105 °C for 60 s and held at that temperature for 30 s. The reaction mixture was drained, the Fmoc group was removed as described before and washed three times with DMF. The following coupling steps were performed in the same way. The final deprotection was performed via addition of a solution of 8% piperidine (DMF, 2.1 mL) for 80 s at 110 °C. The resin solvent was drained and the resin was washed three times with DMF (4 mL each).

### Assembly conversion rate determination

The monomeric peptides were dissolved in TFE at high concentrations (10 mM) before dilution to different concentrations using PBS (50 mM, pH 7.4) and methanol. The final peptide concentrations were 100 μM in PBS (50 mM, pH 7.4, 1% TFE) and 100 μM in MeOH (1% TFE). The samples were incubated at room temperature for 24 h. Then, the samples were filtered through a syringe filter (0.2 µm) and diluted with an internal standard, tyramine (200 µM in MeOH, 1:1, v/v).

Conversion rate measurements were performed on an Atlantis T3 column (4.6 × 100 mm, 5 μm) at a flowrate of 1 mL/min. All measurement steps were performed using gradients of ACN and Milli-Q-H_2_O, each acidified with 0.1% TFA. The gradient started at 0% ACN content and was linearly increased to 100% over 24 min. Absorbance was recorded at 214 and 254 nm wavelengths. The softwares LabSolutions by Shimadzu and Origin Pro by OriginLab^®^ were used to process the generated HPLC spectra. The samples were analyzed in triplicates (*n* = 3).

### Assembly characterization by NMR

To investigate the self-assembly propensity of the three peptide conjugates, the compounds were dissolved in mixtures of TFE-*d*_3_ and PBS-buffered D_2_O with increasing TFE-*d*_3_ content (1:99, 10:90, 20:80, 30:70, 40:60, 50:50, and 100:0, v/v) and analyzed by ¹H NMR spectroscopy. The aqueous component of the solvent mixtures was prepared by lyophilizing 40 mL of commercial PBS to dryness and redissolving the residue in the same volume of D_2_O. Dimethyl sulfone (DMS, 6.96 mg) was added as an internal reference.

Each peptide conjugate was initially dissolved in TFE-*d*_3_, followed by the addition of the DMS-containing PBS/D_2_O buffer (250 µL) and additional PBS/D_2_O buffer without DMS to achieve the desired solvent compositions. The exact volumes used are summarized in Supporting Information File 1, Table S1.

## Supporting Information

File 1 Experimental, characterization data and copies of spectra.

## Data Availability

All data that supports the findings of this study is available in the published article and/or the supporting information of this article.
