# Supplementary material for: The role of spacer length and flexibility in peptide self-assembly
Source: Beilstein J Org Chem. 2026 Jun 25;22:986–96. doi: 10.3762/bjoc.22.77 (PMC13313022; doi:10.3762/bjoc.22.77)
Supplement: File 1 — Experimental, characterization data and copies of spectra. [file Beilstein_J_Org_Chem-22-986-s001.pdf]

BEILSTEIN JOURNAL OF ORGANIC CHEMISTRY

Supporting Information

for

The role of spacer length and flexibility in peptide self-assembly

Julian Link, Albin Lahu, Manfred Wagner, Tanja Weil and David Y. W. Ng

Beilstein J. Org. Chem. **2026,** *22,* 986–996. doi:10.3762/bjoc.22.77

Experimental, characterization data and copies of spectra

License and Terms: This is a supporting information file under the terms of the Creative Commons Attribution License (https://creativecommons.org/licenses/by/4.0). Please note that the reuse, redistribution and reproduction in particular requires that the author(s) and source are credited and that individual graphics may be subject to special legal provisions.
The license is subject to the *Beilstein Journal of Organic Chemistry* terms and conditions: (https://www.beilstein-journals.org/bjoc/terms)

Table of contents

1 Materials and instruments S3

1.1 Materials S3

1.2 Instruments S3

1.2.1 Microwave peptide synthesizer S3

1.2.2 Thin-layer chromatography (TLC) S3

1.2.3 High-performance liquid chromatography (HPLC) S3

1.2.4 Nuclear magnetic resonance spectroscopy (NMR) S4

1.2.5 Liquid chromatography–mass spectrometry (LC–MS) S4

1.2.6 Self-assembly S4

1.2.7 Proteostat® aggregation assay S4

1.2.8 Thioflavin T (ThT) aggregation assay S5

1.2.9 Transmission electron microscopy (TEM) S5

1.2.10 Atomic force microscopy (AFM) S5

1.2.11 Circular dichroism (CD) spectroscopy S5

1.2.12 Assembly conversion rate determination S6

1.2.13 Fluorescence microscopy S6

1.2.14 General procedure for automated peptide synthesis S6

1.2.15 Purification of peptides S7

2 Synthesis S7

2.1 Synthesis of small molecules S7

2.1.1 Synthesis of 4-piperidinyl-1,8-naphthalic anhydride S8

2.1.2 Synthesis of 6-(1,3-dioxo-6-(piperidin-1-yl)-1*H*-benzo[*de*]isoquinolin-2(3*H*)-yl) hexanoic acid (C_6-Nap) S12

2.1.3 Synthesis of 3-(1,3-dioxo-6-(piperidin-1-yl)-1*H*-benzo[*de*]isoquinolin-2(3*H*)-yl)-propanoic acid (C_3-Nap) S16

2.1.4 Synthesis of (2*S*,3*S*)-2-(1,3-dioxo-6-(piperidin-1-yl)-1*H*-benzo[*de*]iso-quinolin-2(3*H*)-yl)-3-methylpentanoic acid (C_0-Nap). S20

2.2 Synthesis of peptides S24

2.2.1 Synthesis of C_6-Nap-ICA S24

2.2.2 Synthesis of C_3-Nap-ICA S26

2.2.3 Synthesis of C_0-Nap-ICA S29

3 Self-assembly S32

3.1 Assembly conversion rate S32

3.2 Thioflavin T (ThT) assay S33

3.3 Fluorescence microscopy, transmission electron and atomic force microscopy... S34

3.4 Circular dichroism (CD) spectroscopy S36

4 NMR study with increasing TFE content S37

References S50

1 Materials and instruments

1.1 Materials

Reagents and solvents were purchased from commercial vendors and used without further purification. For the synthesis of peptides, peptide synthesis grade reagents were used. High performance liquid chromatography (HPLC) was performed using acetonitrile (ACN) in HPLC grade. MilliQ-H_2O for HPLC and reactions was obtained from a Millipore purification system by Merck Millipore.

1.2 Instruments

1.2.1 Microwave peptide synthesizer

A LibertyBlue 2.0 automated microwave synthesizer from CEM Corporation was used to perform solid-phase peptide synthesis (SPPS). The system was purged with dimethylformamide (DMF) prior and during usage. Piperidine was used for deprotection of the peptide protecting groups and the used amino acids were dissolved in DMF. *N*,*N*'-Diisopropylcarbodiimide (DIC) and ethyl cyanohydroxyiminoacetate (Oxyma Pure) were employed as coupling agents and dissolved in DMF as well.

1.2.2 Thin-layer chromatography (TLC)

The analysis of compounds by means of thin-layer chromatography (TLC) was performed using Alugram Sil G/UV_{254} plates, by Macherey-Nagel. The analytes were detected by extinction of ultraviolet (UV) absorption at a wavelength of 254 nm and 365 nm or by staining of the TLC plate using potassium permanganate solution.

1.2.3 High-performance liquid chromatography (HPLC)

Peptides were purified by preparative HPLC using a setup from Shimadzu. For purification, a Phenomenex Gemini 5 µm NX-C18 110 Å 150 × 30 mm column was used at a flow rate of 25 mL/min. All measurements and purification steps were performed using gradients of ACN and MilliQ-H_2O, each acidified with 0.1% trifluoroacetic acid (TFA). The absorbance was recorded at wavelengths of 214 and 254 nm. For processing of the HPLC spectra, the software LabSolutions by Shimadzu and PowerPoint by Microsoft Corporation were used. Analytical measurements were performed on an Atlantis T3 column (4.6 × 100 mm, 5 µm) at a flowrate of 1 mL/min. All measurements and purification steps were performed using gradients of ACN and MilliQ-H_2O, each acidified with 0.1% TFA. Absorbance was recorded at 214 and 254 nm wavelengths. The software LabSolutions by Shimadzu and PowerPoint by Microsoft Corporation were used to process the generated HPLC spectra.

1.2.4 Nuclear magnetic resonance spectroscopy (NMR)

^{1}H and ^{13}C NMR-measurements were conducted with AVANCE NEO 400- and AVANCE 700-spectrometers by Bruker at 298 K in deuterated solvents. All deuterated solvents were used without further purification and purchased from Sigma Aldrich. The internal standards were set depending on the solvent used.

$CHCl_3$: ^{1}H: δ = 7.26 ppm, ^{13}C: δ = 77.16 ppm; DMSO-d_5H: ^{1}H: δ = 2.50 ppm, ^{13}C: δ = 39.52 ppm; CF_3CHDOD: ^{1}H: δ = 3.88 ppm, ^{13}C: δ = 61.50, 126.30 ppm.

The acquired data was processed using the software MestReNova 14.2.0 by Mestrelab Research. The chemical shifts δ were indicated in parts per million (ppm). Multiplets were abbreviated as following: s = singlet, d = doublet, t = triplet, q = quartet, p = quintet, m = multiplet.

1.2.5 Liquid chromatography–mass spectrometry (LC–MS)

Compounds were analyzed by HPLC–ESIMS on an LC–MS 2020 from Shimadzu using a Kinetex 2.6 µm EVO C_{18} 100 Å LC 50 × 2.1 mm column. MilliQ-H_2O, acidified with 0.1% formic acid, and ACN, acidified with 0.1% formic acid, were used as solvents for all measurements. The solvent gradient started with 5% ACN and 95% MilliQ-H_2O. This solvent ratio was kept constant for 2 min, then the ACN content was linearly increased to 95% within 14 min. The generated data was processed with LabSolutions by Shimadzu and Origin Pro by OriginLab®.

1.2.6 Self-assembly

The monomeric peptides were dissolved in 2,2,2-trifluoroethanol (TFE) at high concentrations (20/10 mM) before dilution using phosphate-buffered saline (PBS) buffer (50 mM, pH 7.4) with final TFE concentrations of 1%. The samples were incubated at room temperature for 24 h.

1.2.7 Proteostat® aggregation assay

The peptide samples were assembled according to the general procedure. The Proteostat® Protein Aggregation Assay Kit was purchased from Enzo Life Sciences, Inc. 0.52 µL of the Proteostat stock solution was diluted with 98.48 µL MilliQ-H_2O and 1.0 µL assay buffer. 5 µL of this solution was added to 45 µL peptide solution in a Greiner 96 black well plate. The solutions were incubated in the dark at room temperature for 15 min while shaking at 500 rpm. The fluorescence intensity was measured with bandwidths of 20 nm and an excitation wavelength of λ_{ex} = 550 nm as well as an emission wavelength of λ_{em} = 600 nm. The experiment was repeated three times. Control measurements were performed by incubating Proteostat® solution similarly in PBS buffer (50 mM, pH 7.4, 1% TFE).

1.2.8 Thioflavin T (ThT) aggregation assay

The peptide samples were assembled according to the general procedure. 50 µM Thioflavin T (ThT) was added and the samples were incubated for 15 min at room temperature in the dark at 500 rpm. The fluorescence intensity was measured with bandwidths of 10 nm and an excitation wavelength of λ_{ex} = 450 nm as well as an emission wavelength of λ_{em} = 482 nm in a Greiner 96 well plate black. The experiment was repeated three times. Control measurements were performed by incubating ThT similarly in PBS buffer (50 mM, pH 7.4, 1% TFE).

1.2.9 Transmission electron microscopy (TEM)

TEM images were recorded on a JEOL 1400 transmission electron microscope at a voltage of 120 kV. The images were visualized using the software ImageJ. Unless stated otherwise, all samples were prepared in PBS (50 mM, pH 7.4, 1% TFE) and incubated for 24 h at room temperature. The samples were applied to Formvar/carbon-film coated copper grids (300 mesh) by Plano GmbH. The TEM grids were prepared by covering the grid with a 3 µL droplet of the sample solution and incubation for 5 minutes. The excess solution was removed and the grids were stained with 7 µL uranyl(IV) acetate solution (4%) for 2.5 min. The TEM grids were washed three times with MilliQ-H_2O and dried before being used for microscopy.

1.2.10 Atomic force microscopy (AFM)

Atomic force microscopy (AFM) (Park NX20 and Bruker Dimension ICON) was employed to characterize the morphology of the system. The samples were assembled according to the general procedure in MilliQ-H_2O (1% TFE) air-dried prior casting on freshly cleaved silicon wafers. Imaging was performed under tapping mode using a cantilever with a resonance frequency of 285 kHz and a spring constant of 42 $N \cdot m^{-1}$. The resulting AFM images were further processed and analyzed with Gwyddion 2.63.

1.2.11 Circular dichroism (CD) spectroscopy

CD spectra were recorded on a JASCO J-1500 spectrometer in a 1 mm high precision cell by Hellma Analytics. The collected data was processed using Spectra Analysis by JASCO and Origin Pro by OriginLab®. Samples were prepared in 50 mM PBS buffer (pH 7.4) with peptide concentrations of 100 µM and 1% TFE. The samples were incubated for 24 h at room temperature and the spectra were recorded at wavelengths from 185 nm to 300 nm with a bandwidth of 1 nm, data pitch of 0.2 nm, and scanning speed at 5 nm/min, while using 300 µL of the prepared sample solution. The samples were measured three times and the collected spectra were averaged. Background measurements were performed using PBS buffer (50 mM, pH 7.4, 1% TFE, 300 µL) and subtracted from the peptide spectra.

1.2.12 Assembly conversion rate determination

The monomeric peptides were dissolved in TFE at high concentrations (10 mM) before dilution to different concentrations using PBS (50 mM, pH 7.4) and methanol. The final peptide concentrations were 100 µM in PBS (50 mM, pH 7.4, 1% TFE) and 100 µM in MeOH (1% TFE). The samples were incubated at room temperature for 24 h. Then, the samples were filtered through a syringe filter (0.2 µm) and diluted with an internal standard, tyramine (200 µM in MeOH, 1:1, *v/v*).

Conversion rate measurements were performed on an Atlantis T3 column (4.6 × 100 mm, 5 µm) at a flowrate of 1 mL/min. All measurement steps were performed using gradients of ACN and MilliQ-H_2O, each acidified with 0.1% TFA. The gradient started at 0% ACN content and was linearly increased to 100% over 24 min. Absorbance was recorded at 214 and 254 nm wavelengths. The software LabSolutions by Shimadzu and Origin Pro by OriginLab® were used to process the generated HPLC spectra.

1.2.13 Fluorescence microscopy

The samples were incubated according to the general procedure for self-assembly. Fluorescence images of the peptides were taken using a Keyence BZ-X810 fluorescence microscope with a 40× LD PH lens. The fluorescence of the naphthalene moiety was analyzed using a GFP filter with the following wavelength settings: λ_{ex} = 470/40 nm, λ_{em} = 525/50 nm.

1.2.14 General procedure for automated peptide synthesis

Peptides were synthesized using the Fmoc-SPPS strategy [1], synthesizing the peptide from *C*- to *N*-terminus in a microwave-assisted peptide synthesizer on an Fmoc-ʟ-Ala Clt-resin at scales of 0.10 mmol. Fmoc-ʟ-Ala Clt-resin (0.10 mmol) was swollen in DMF (15 mL) at room temperature for one hour before transferring it into the peptide synthesizer. In the peptide synthesizer, DMF was removed through the draining process and DMF (3.5 mL) was added prior to the coupling. Prior to every coupling, the Fmoc-protecting group was cleaved by one deprotection step using a solution of 8% piperidine (DMF, 2.1 mL) for 80 s at 110 °C. The amino acids (0.5 M in DMF, 1.0 mL), DIC (0.75 M in DMF, 1.0 mL) and Oxyma Pure (0.26 M in DMF, 1.5 mL) were added to the resin and the solution was heated to 105 °C for 60 s and held at that temperature for 30 s. The reaction mixture was drained, the Fmoc group was removed as described before and washed three times with DMF. The following coupling steps were performed in the same way. The final deprotection was performed via addition of a solution of 8% piperidine (DMF, 2.1 mL) for 80 s at 110 °C. The resin solvent was drained and the resin was washed three times with DMF (4 mL each).

1.2.15 Purification of peptides

Crude peptide products were purified via HPLC. The crudes were dissolved in 20 mL of ACN/MilliQ-H_2O 9:1 (*v*/*v*, 5% TFA) and filtered through a syringe filter (0.2 µm). For purification, a Phenomenex Gemini 5 µm NX-C18 110 Å 150 × 30 mm column was used at a flowrate of 25 mL/min. The gradient started at 0% ACN (0.1% TFA) and was linearly increased to 100% ACN (0.1% TFA) through the span of 25 min. Fractions were collected and detected by measurements of the absorbance at wavelengths of 214 and 254 nm. The compounds were then identified using LC–MS and NMR spectroscopy. The product fractions were collected in a round-bottomed flask (RBF) and narrowed down to 40 mL under reduced pressure. The residual solvents were removed via lyophilization overnight.

2 Synthesis

2.1 Synthesis of small molecules

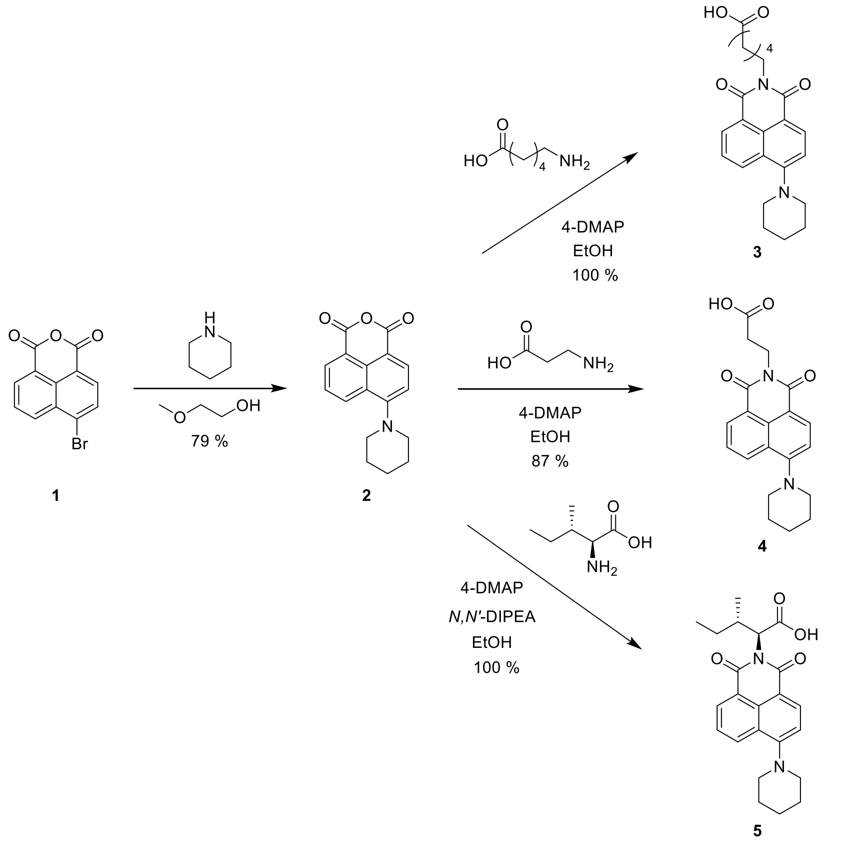

Scheme S1: Two-step synthesis of the naphthalene-π-blocks C_6-Nap **3**, C_3-Nap **4** and C_0-Nap **5** starting from 4-bromo-1,8-naphthalic anhydride (**1**).

2.1.1 Synthesis of 4-piperidinyl-1,8-naphthalic anhydride

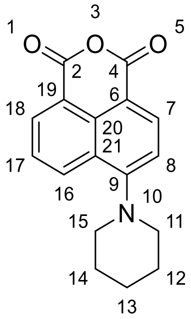

The synthesis was performed according to a general procedure by Li et al. [2] 4-Bromo-1,8-naphtalic anhydride (**1**, 1.00 g; 3.61 mmol; 1.0 equiv) was dissolved in 2-methoxyethanol (10 mL) and stirred for 10 min at room temperature. Then, piperidine (750 µL; 7.22 mmol; 2.0 equiv) was added and the reaction mixture was stirred overnight under reflux conditions. The reaction mixture was cooled to room temperature. The suspension was further cooled in an ice bath and filtered. The orange solid was washed three times with small amounts of cold EtOH and the orange crude was recrystallized from EtOH. 4-Piperidinyl-1,8-naphtalic anhydride (**2**, 800 mg; 2.85 mmol; 79%) was obtained as orange crystals and used without further purification.

LC–MS: (pos.): calculated for $[M + H]^+$: m/z = 282.3; measured: m/z = 282.1.

R_f: 0.58 (DCM/MeOH = 9:1, *v/v*).

^{1}H NMR, COSY (400 MHz, $CDCl_3$, 298 K): δ [ppm] = 8.58 (dd, 1H, J = 7.3 Hz, 1.2 Hz, H_{16}); 8.50 (d, 1H, J = 8.2 Hz, H_{18}); 8.43 (dd, 1H, J = 8.5 Hz, 1.2 Hz, H_7); 7.71 (dd, 1H, J = 8.5 Hz, 7.3 Hz, H_{17}); 7.19 (d, 1H, J = 8.2 Hz, H_8); 3.33–3.25 (m, 4H, $H_{11,15}$); 1.90 (p, 4H, J = 5.8 Hz, $H_{12,14}$); 1.75 (tq, 2H, J = 7.9 Hz, 4.1 Hz, H_{13}).

^{13}C NMR, HSQC, HMBC (101 MHz, $CDCl_3$, 298 K): δ [ppm] = 161.7, 160.9, 158.6, 135.2, 133.3, 132.3, 125.8, 115.1, 54.5, 26.3, 24.4.

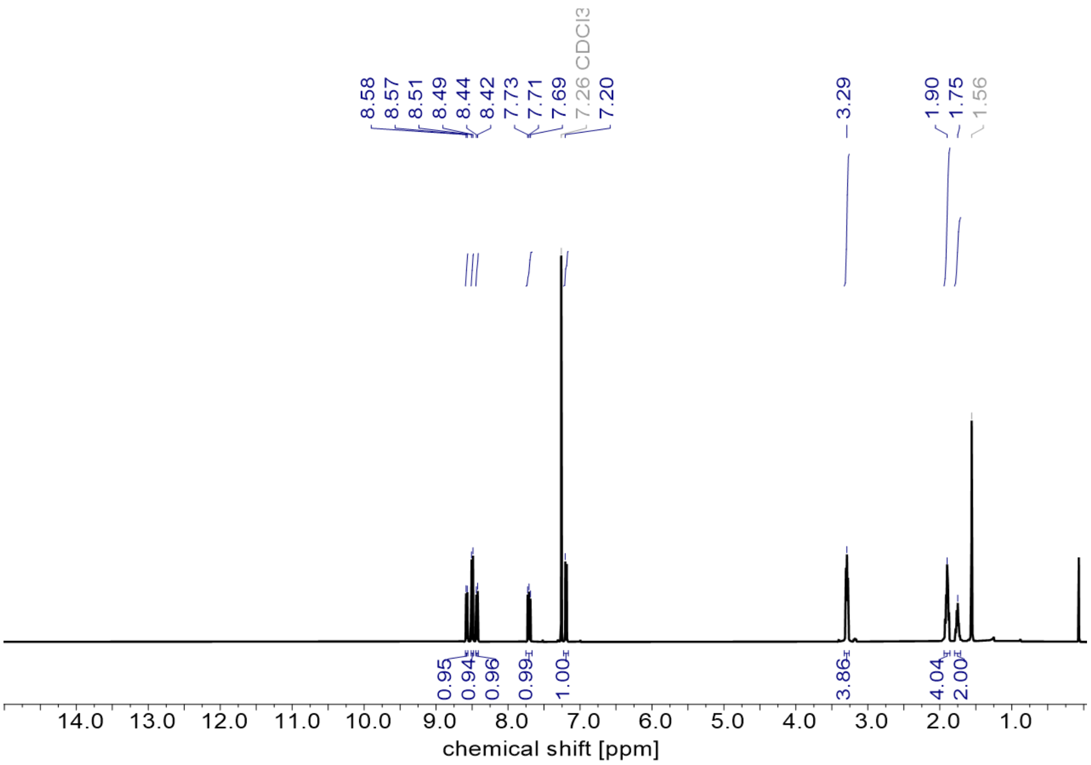

Figure S1: ^{1}H NMR spectrum ($CDCl_3$, 400 MHz, 298 K) of 4-piperidinyl-1,8-naphthalic anhydride (**2**).

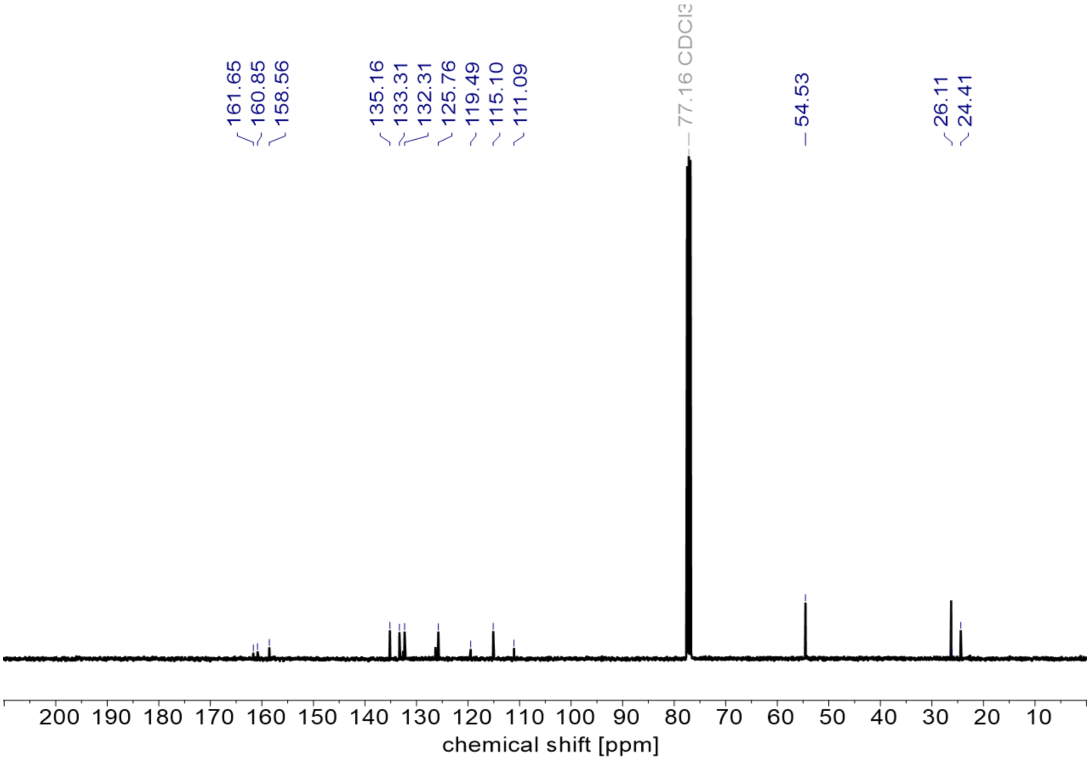

Figure S2: ^{13}C NMR spectrum ($CDCl_3$, 101 MHz, 298 K) of 4-piperidinyl-1,8-naphthalic anhydride (**2**).

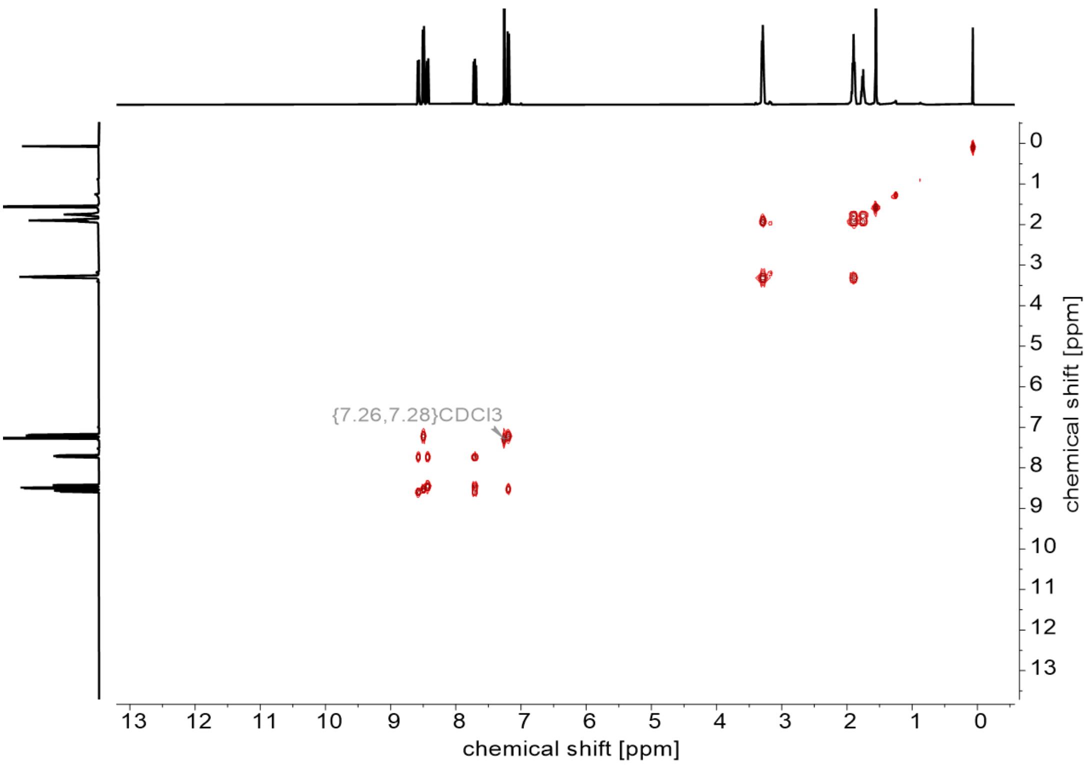

Figure S3: $^{1}H,^{1}H$-COSY-NMR spectrum ($CDCl_3$, 400 MHz, 298 K) of 4-piperidinyl-1,8-naphthalic anhydride (**2**).

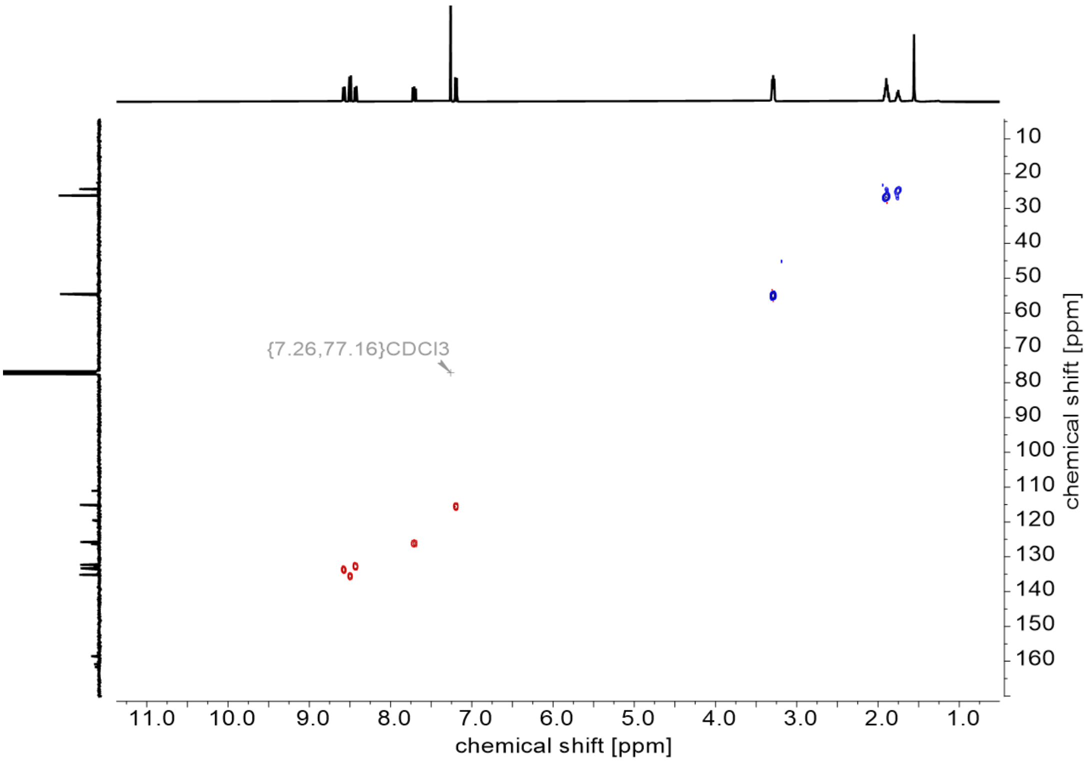

Figure S4: $^{1}H,^{13}C$-HSQC-NMR spectrum ($CDCl_3$, 101 MHz, 298 K) of 4-piperidinyl-1,8-naphthalic anhydride (**2**).

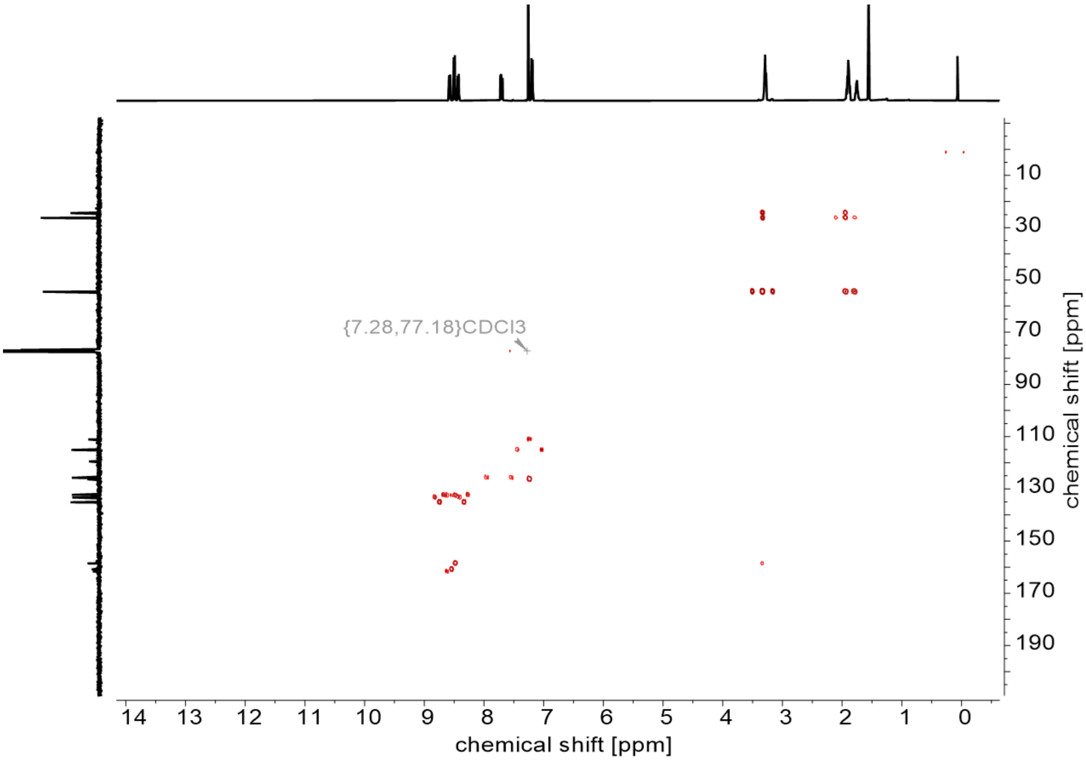

Figure S5: ^{1}H,^{13}C-HMBC-NMR spectrum ($CDCl_3$, 101 MHz, 298 K) of 4-piperidinyl-1,8-naphthalic anhydride (**2**).

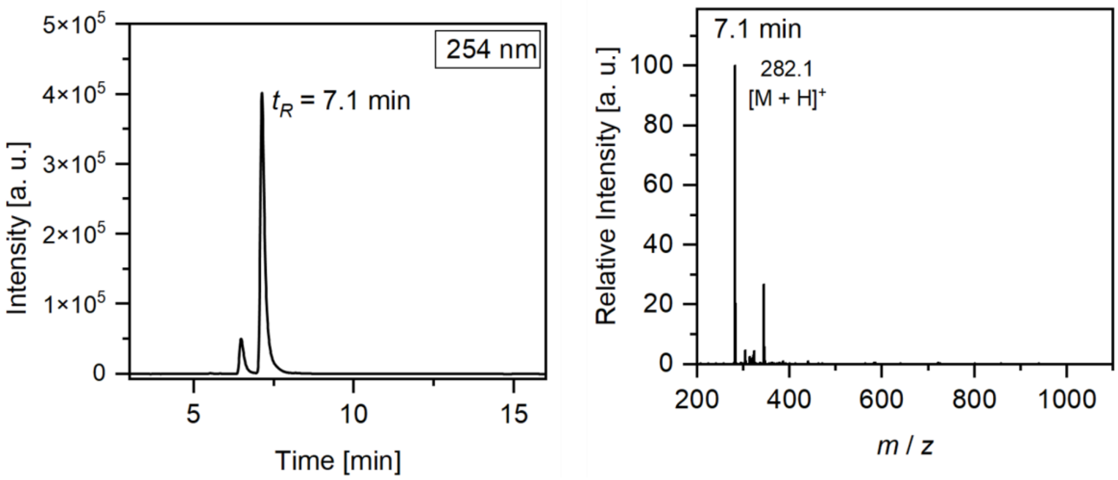

Figure S6: LC elugram (left, detection at 214 nm) and corresponding mass data (right) of 4-piperidinyl-1,8-naphthalic anhydride (**2**).

2.1.2 Synthesis of 6-(1,3-dioxo-6-(piperidin-1-yl)-1*H*-benzo[*de*]isoquinolin-2(3*H*)-yl) hexanoic acid (C_6-Nap)

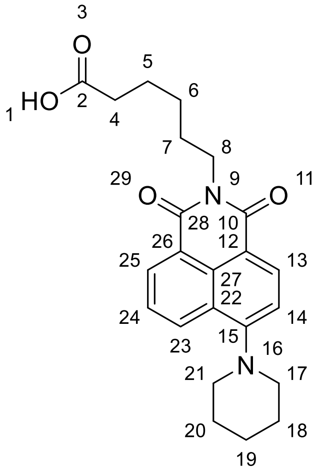

The synthesis was performed with a modified procedure referenced to Mata et al.[3] 4-Piperidinyl-1,8-naphthalic anhydride (**2**, 100 mg; 0.36 mmol; 1.0 equiv), 6-aminohexanoic acid (55 mg; 0.43 mmol; 1.2 equiv) and 4-DMAP (5 mg; 0.04 mmol; 0.1 equiv) were covered with EtOH (10 mL). The reaction mixture was stirred under reflux conditions for 24 h. Then, the mixture was diluted with DCM (10 mL). The solution was washed with 1 M HCl (3 × 5 mL) and brine (1 × 10 mL). The organic layer was dried over $MgSO_4$, filtered and the solvents were removed under reduced pressure. C_6-Nap **3** (140 mg; 0.36 mmol; 100%) was obtained as a bright orange solid.

LC–MS: (pos.): calculated for $[M + H]^+$: *m*/*z* = 395.5; measured: *m/z* = 395.2; calculated for $[2 M + Na]^+$: *m*/*z* = 811.9; measured: *m*/*z* = 811.5.

R*f*: 0.15 (DCM/MeOH = 9:1, *v/v*; 1 % AcOH).

^{1}H NMR, COSY (400 MHz, $CDCl_3$, 298 K): δ [ppm] = 8.57 (dd, 1H, *J* = 7.2 Hz, 1.2 Hz, H_{23}); 8.49 (d, 1H, *J* = 8.1 Hz, H_{25}); 8.39 (dd, 1H, *J* = 8.5 Hz, 1.3 Hz, H_{13}); 7.67 (dd, 1H, *J* = 8.5 Hz, 7.3 Hz, H_{24}); 7.17 (d, 1H, *J* = 8.2 Hz, H_{14}); 4.21–4.13 (m, 2H, H_8); 3.33–3.19 (m, 4H, $H_{17,21}$); 2.38 (t, 2H, *J* = 7.5 Hz, H_4); 1.88 (p, 4H, *J* = 5.8 Hz, $H_{18,20}$); 1.82–1.66 (m, 6H, $H_{5,7,19}$); 1.48 (tt, 2H, *J* = 9.9 Hz, 6.4 Hz, H_6).

^{13}C NMR, HSQC, HMBC (101 MHz, $CDCl_3$, 298 K): δ [ppm] = 157.5, 132.9, 131.2, 130.8, 126.4, 125.5, 123.3, 116.1, 114.9, 54.7, 53.6, 40.1, 33.7, 27.9, 26.7, 26.4, 24.6, 24.5.

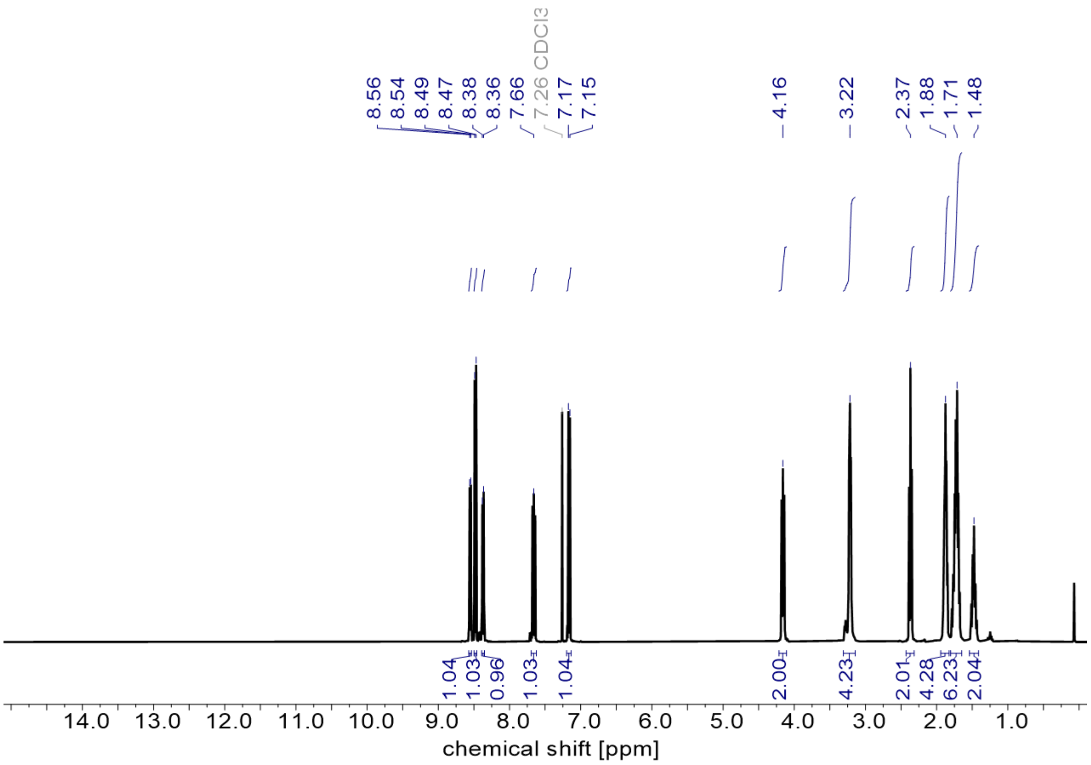

Figure S7: ^{1}H NMR spectrum ($CDCl_3$, 400 MHz, 298 K) of C_6-Nap **3**.

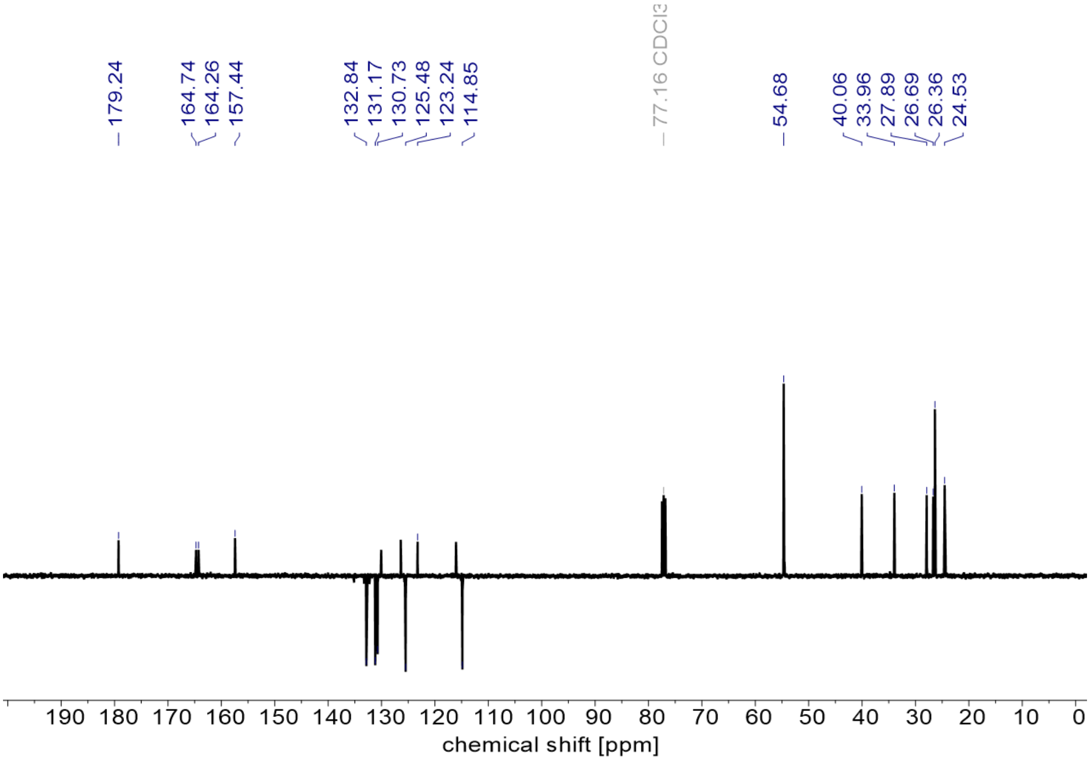

Figure S8: ^{13}C NMR spectrum ($CDCl_3$, 101 MHz, 298 K) of C_6-Nap **3**.

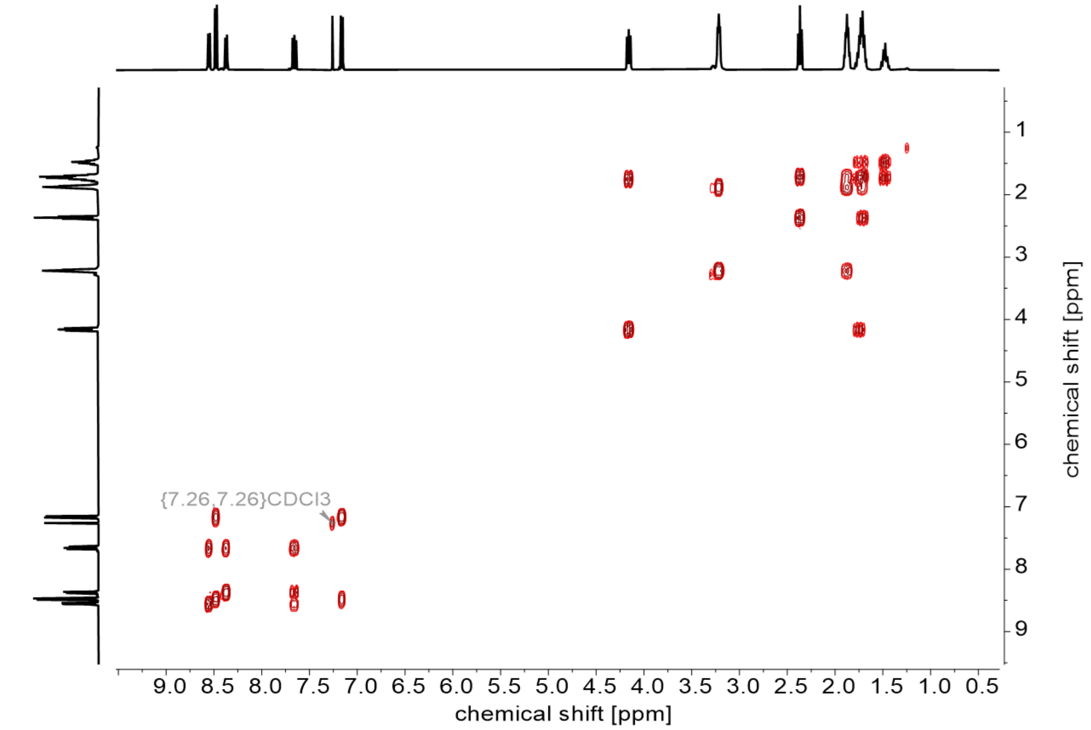

Figure S9: $^{1}H,^{1}H$-COSY-NMR spectrum ($CDCl_3$, 400 MHz, 298 K) of C_6-Nap **3**.

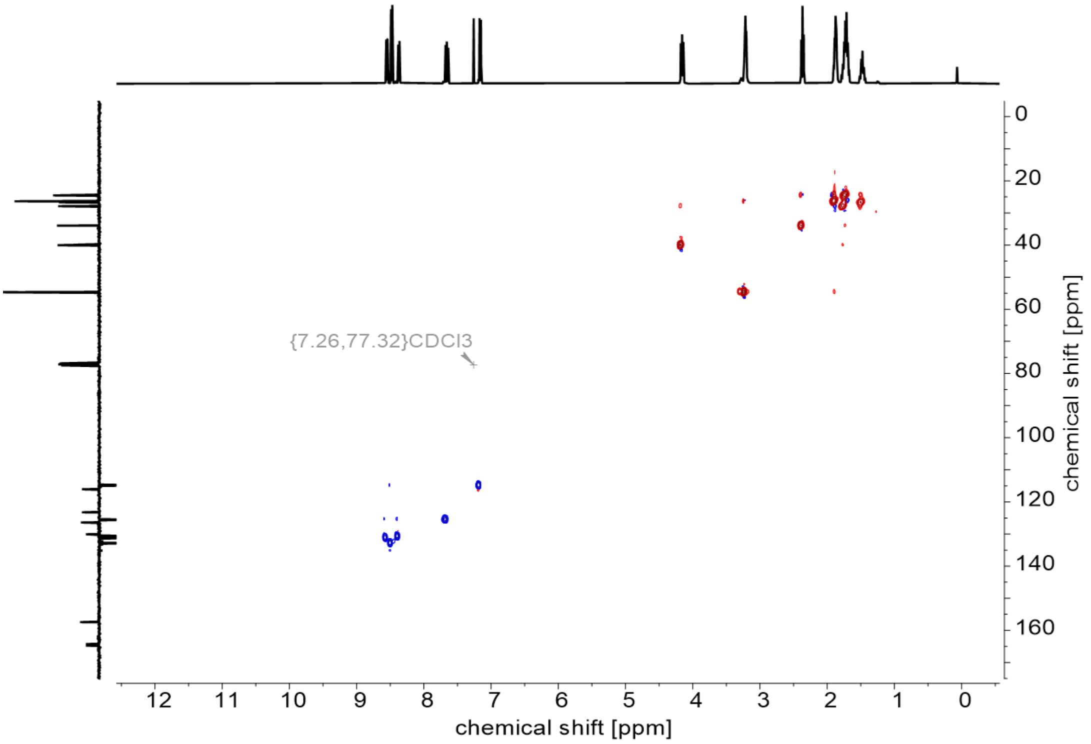

Figure S10: $^{1}H,^{13}C$-HSQC-NMR spectrum ($CDCl_3$, 101 MHz, 298 K) of C_6-Nap **3**.

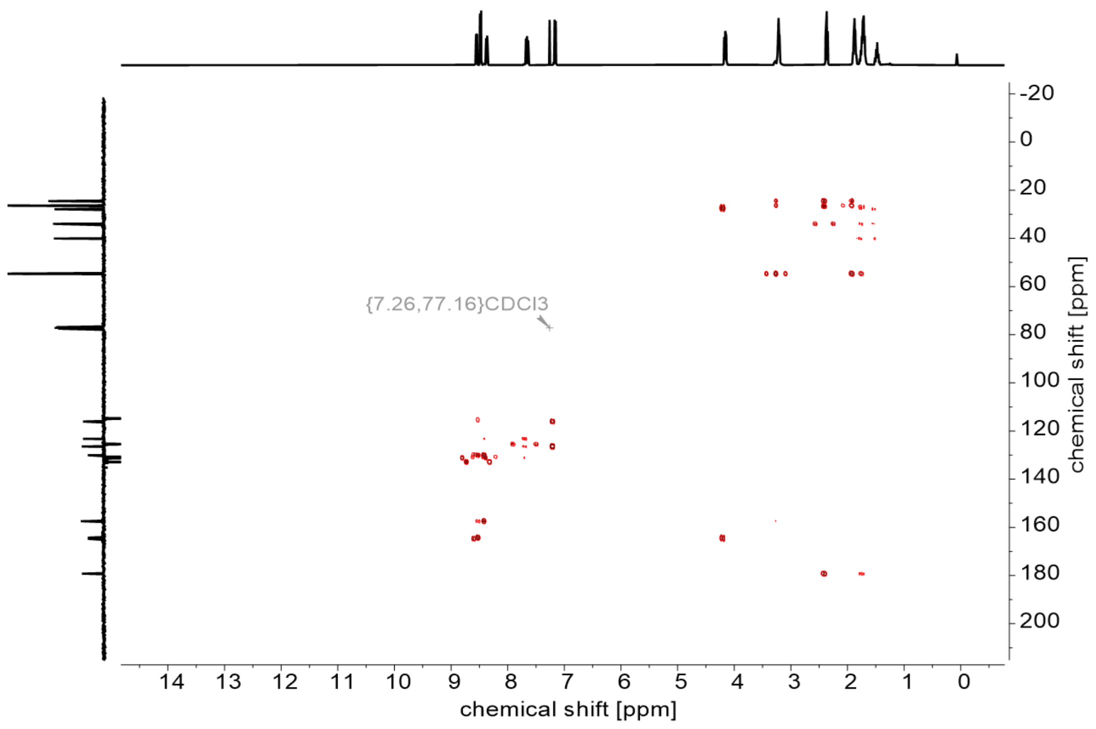

Figure S11: $^{1}H,^{13}C$-HMBC-NMR spectrum ($CDCl_3$, 101 MHz, 298 K) of C_6-Nap **3**.

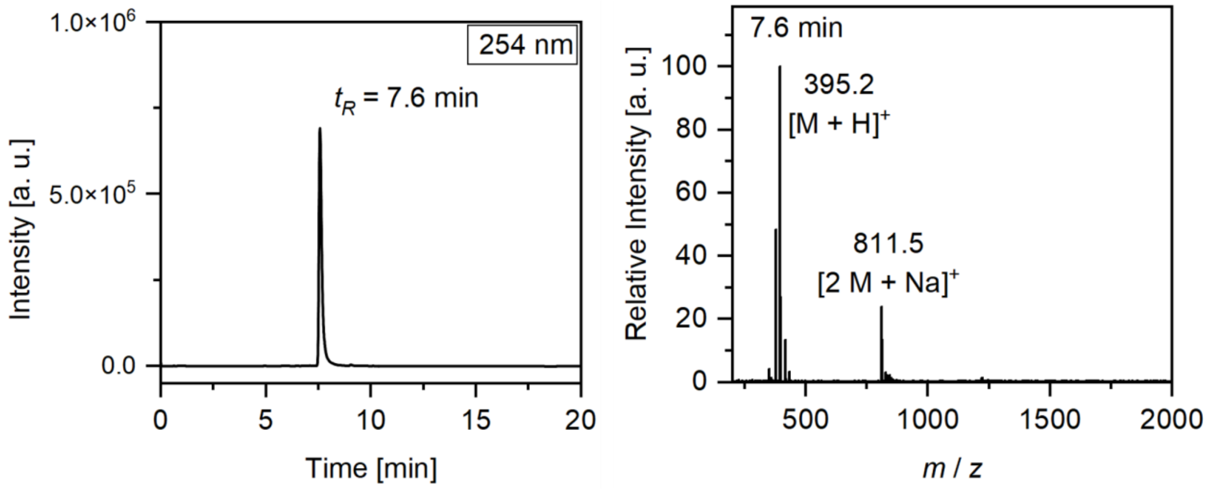

Figure S12: LC elugram (left, detection at 254 nm) and corresponding mass data (right) of C_6-Nap **3**.

2.1.3 Synthesis of 3-(1,3-dioxo-6-(piperidin-1-yl)-1*H*-benzo[*de*]isoquinolin-2(3*H*)-yl)-propanoic acid (C_3-Nap)

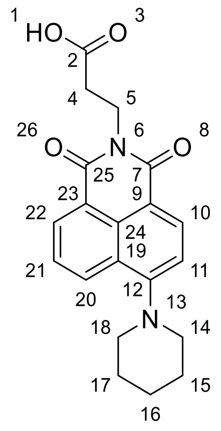

The synthesis was performed with a modified procedure referenced to Li et al.[2] 4-Piperidinyl-1,8-naphthalic anhydride (**2**, 100 mg; 0.36 mmol; 1.0 equiv), β-alanine (32 mg; 0.36 mmol; 1.0 equiv) and 4-DMAP (5 mg; 0.04 mmol; 0.1 equiv) were covered with EtOH (10 mL). The reaction mixture was stirred under reflux conditions for 24 h. The mixture was diluted with DCM (10 mL) and the solution was washed with 1 M HCl (3 × 5 mL) and brine (1 × 10 mL). The organic layer was dried over $MgSO_4$, filtered and the solvents were removed under reduced pressure. C_3-Nap **4** (110 mg; 0.31 mmol; 87%) was obtained as an orange solid.

LC–MS: (pos.): calculated for $[M + H]^+$: m/z = 353.4; measured: m/z = 353.2; calculated for $[2 M + Na]^+$: m/z = 727.8; measured: m/z = 727.4.

R_f: 0.72 (DCM/MeOH = 7:3, *v/v*; 1 % AcOH).

^{1}H NMR, COSY (400 MHz, $CDCl_3$, 298 K): δ [ppm] = 8.58 (dd, 1H, J = 7.3 Hz, 1.2 Hz, H_{20}); 8.50 (d, 1H, J = 8.1 Hz, H_{22}); 8.40 (dd, 1H, J = 8.4 Hz, 1.2 Hz, H_{10}); 7.68 (dd, 1H, J = 8.4 Hz, 7.3 Hz, H_{21}); 7.18 (d, 1H, J = 8.1 Hz, H_{11}); 4.50 (dd, 2H, J = 7.9 Hz, 6.9 Hz, H_5); 3.24 (t, 4H, J = 5.3 Hz, $H_{14,18}$); 2.83 (dd, 2H, J = 7.9 Hz, 7.0 Hz, H_4); 1.89 (p, 4H, J = 5.6 Hz, $H_{15,17}$); 1.73 (t, 2H, J = 5.8 Hz, H_{16}).

^{13}C NMR, HSQC, HMBC (101 MHz, $CDCl_3$, 298 K): δ [ppm] = 175.0, 164.7, 164.2, 157.8, 133.1, 131.4, 131.1, 130.2, 126.4, 125.5, 123.0, 115.7, 114.9, 54.7, 35.8, 32.6, 26.4, 24.5.

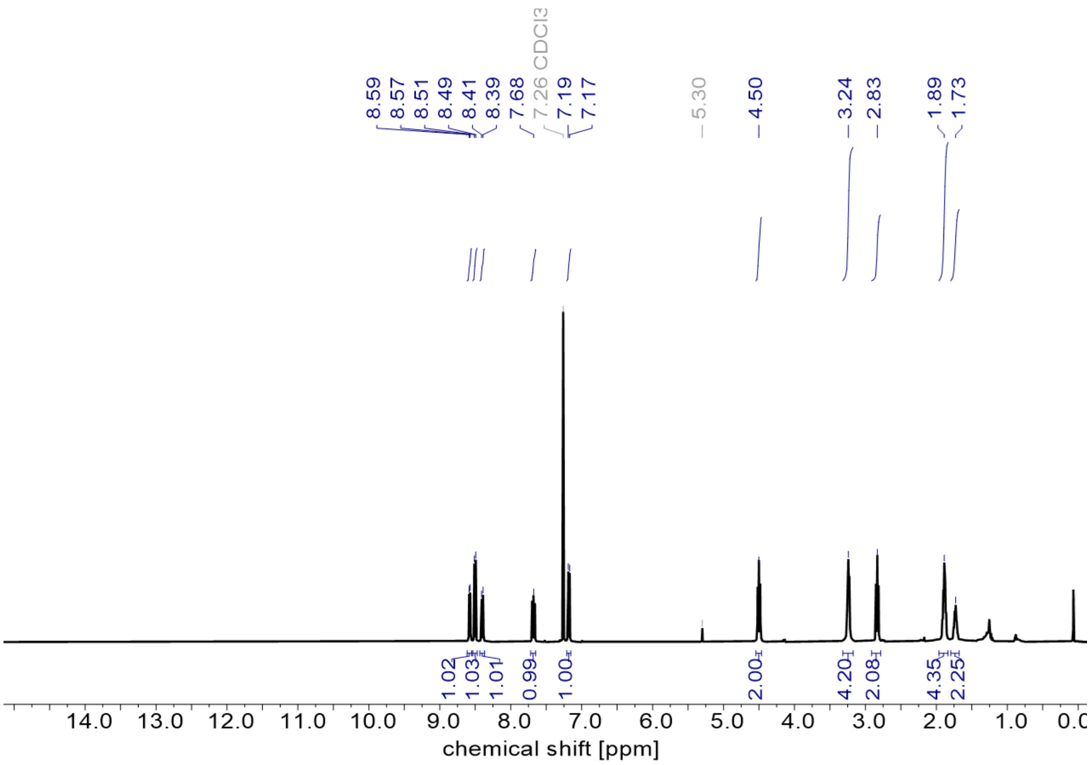

Figure S13: ^{1}H NMR spectrum ($CDCl_3$, 400 MHz, 298 K) of C_3-Nap **4**.

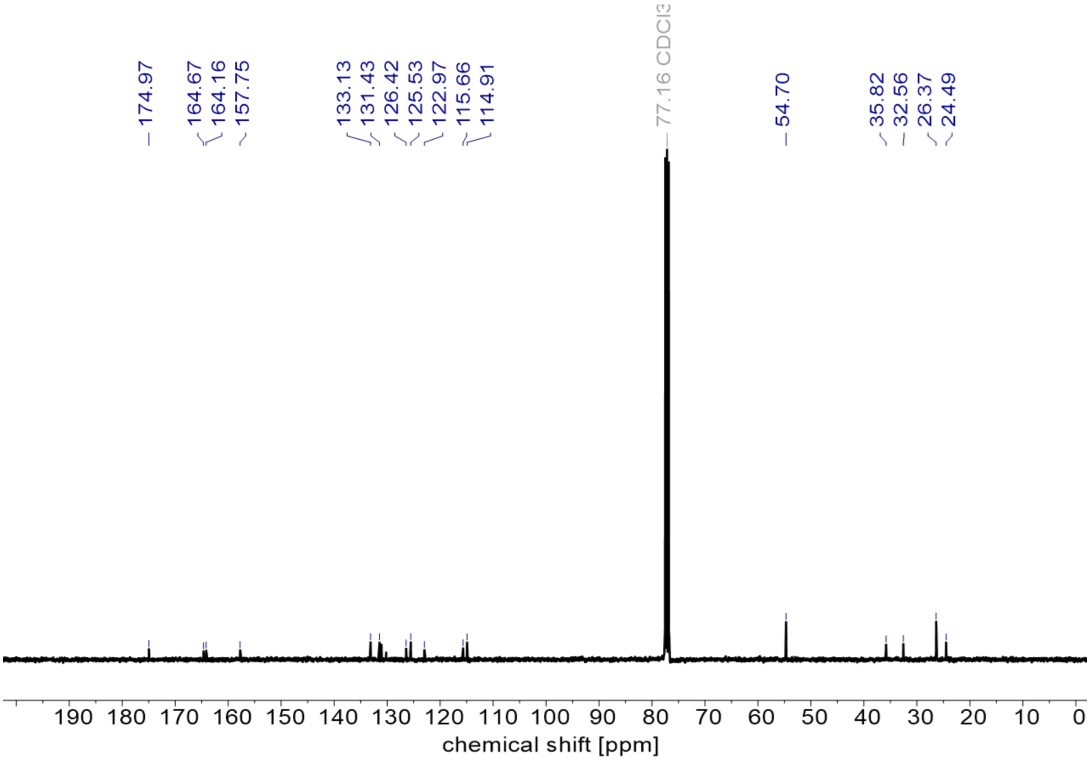

Figure S14: ^{13}C NMR spectrum ($CDCl_3$, 101 MHz, 298 K) of C_3-Nap **4**.

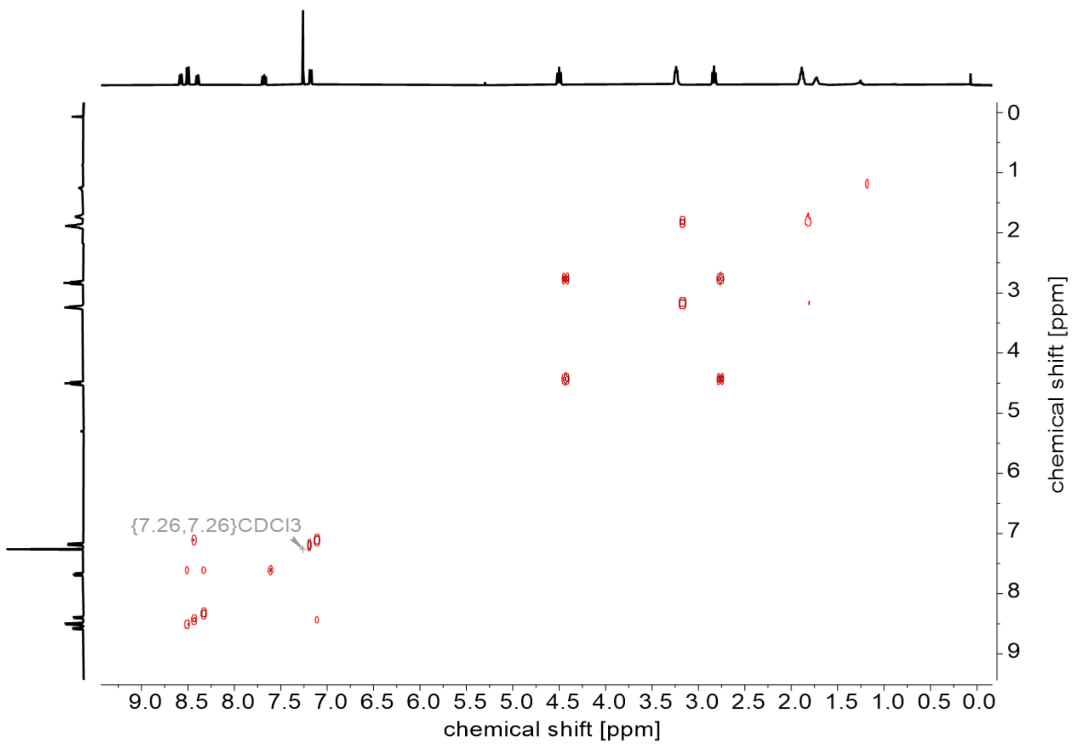

Figure S15: $^{1}H,^{1}H$-COSY-NMR spectrum ($CDCl_3$, 400 MHz, 298 K) of C_3-Nap **4**.

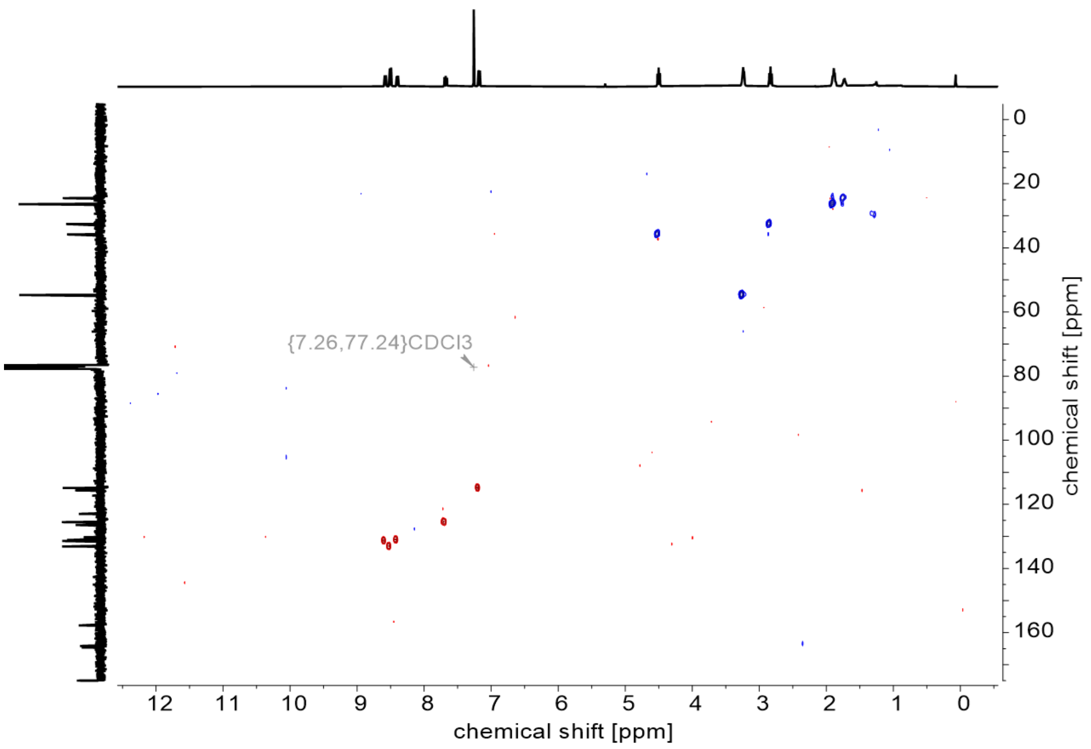

Figure S16: $^{1}H,^{13}C$-HSQC-NMR spectrum ($CDCl_3$, 101 MHz, 298 K) of C_3-Nap **4**.

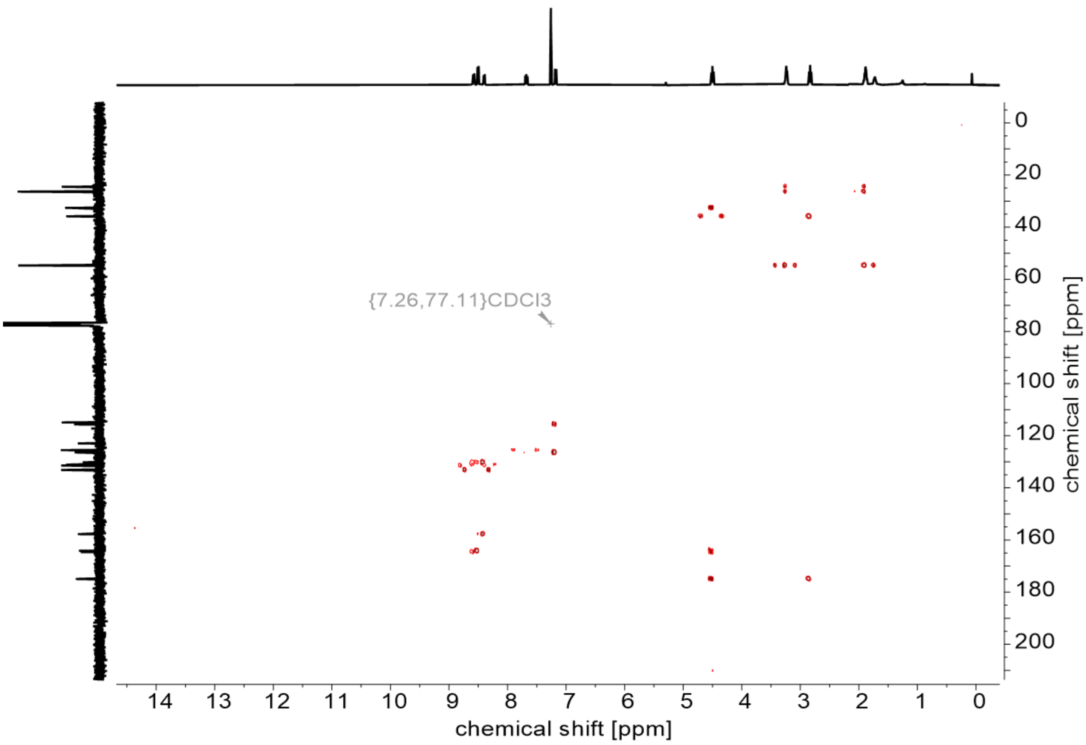

Figure S17: $^{1}H,^{13}C$-HMBC-NMR spectrum ($CDCl_3$, 101 MHz, 298 K) of C_3-Nap **4**.

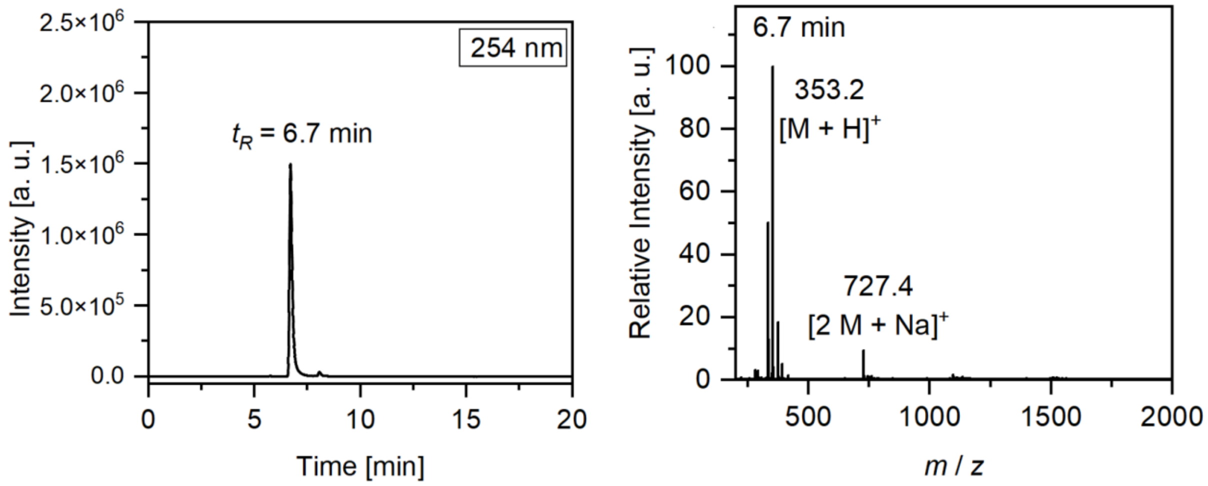

Figure S18: LC elugram (left, detection at 254 nm) and corresponding mass data (right) of C_3-Nap **4**.

2.1.4 Synthesis of (2*S*,3*S*)-2-(1,3-dioxo-6-(piperidin-1-yl)-1*H*-benzo[*de*]isoquinolin-2(3*H*)-yl)-3-methylpentanoic acid (C_0-Nap).

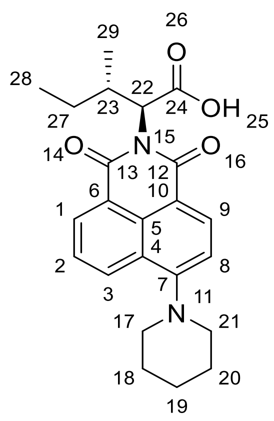

4-Piperidinyl-1,8-naphtalic anhydride (**2**, 200 mg; 0.71 mmol; 1.0 equiv), ʟ-isoleucine (115 mg; 0.85 mmol; 1.2 equiv) and 4-DMAP (9 mg; 0.07 mmol; 0.1 equiv) were covered with EtOH (10 mL). *N,N'*-DIPEA (150 µL; 0.85 mmol; 1.2 equiv) was added in one portion. The reaction mixture was stirred under reflux conditions overnight and diluted with DCM (10 mL). The solution was washed with 1 M HCl (3 × 5 mL) and brine (1 × 10 mL). The organic layer was dried over $MgSO_4$, filtered and the solvents were removed under reduced pressure. C_0-Nap **5** (280 mg; 0.71 mmol; 100%) was obtained as an orange solid.

LC–MS: (pos.): calculated for $[M + H]^+$: *m/z* = 395.5; measured: *m/z* = 395.2; calculated for $[2 M + Na]^+$: *m/z* = 811.9; measured: *m/z* = 811.5.

R_f: 0.75 (EtAc/^{C}Hex = 1:2, *v/v*; 1 % TFA).

^{1}H NMR, COSY (400 MHz, $CDCl_3$, 298 K): δ [ppm] = 8.57 (dd, 1H, J = 7.3 Hz, 1.2 Hz, H_3); 8.50 (d, 1H, J = 8.1 Hz, H_1); 8.40 (dd, 1H, J = 8.4 Hz, 1.2 Hz, H_9); 7.68 (dd, 1H, J = 8.4 Hz, 7.3 Hz, H_2); 7.18 (d, 1H, J = 8.1 Hz, H_8); 5.47 (d, 1H, J = 9.3 Hz, H_{22}); 3.24 (t, 4H, J = 5.3 Hz, $H_{17,21}$); 2.62 (tdd, 1H, J = 10.1 Hz, 6.6 Hz, 3.7 Hz, H_{23}); 1.88 (p, 4H, J = 5.6 Hz, $H_{18,20}$); 1.73 (q, 2H, J = 6.2 Hz, H_{19}); 1.34–1.26 (m, 1H, $H_{27'}$); 1.24 (d, 3H, J = 6.5 Hz, H_{29}); 1.09–0.91 (m, 1H, $H_{27''}$); 0.80 (t, 3H, J = 7.4 Hz, H_{28}).

^{13}C NMR, HSQC, HMBC (101 MHz, $CDCl_3$, 298 K): δ [ppm] = 174.3, 164.6, 164.1, 157.9, 133.6, 131.9, 131.3, 130.4, 126.4, 125.6, 122.7, 115.2, 114.9, 58.2, 54.7, 33.5, 31.1, 26.4, 25.2, 24.5, 18.0, 11.2.

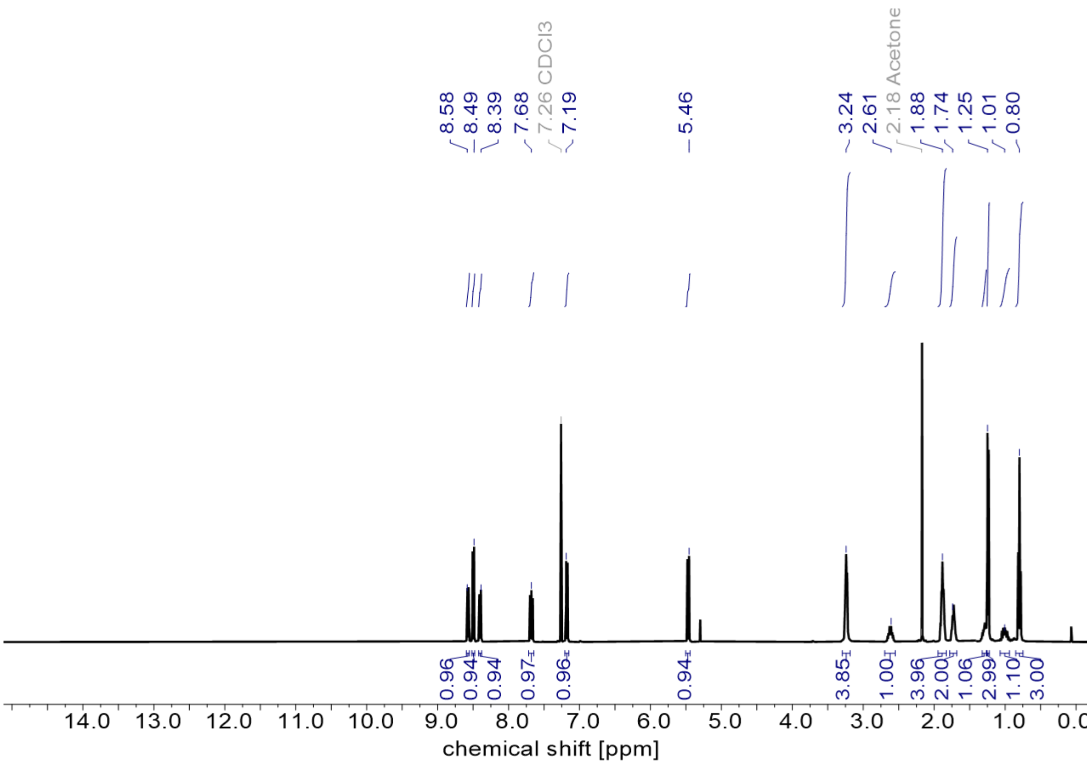

Figure S19: ^{1}H NMR spectrum ($CDCl_3$, 400 MHz, 298 K) of C_0-Nap **5**.

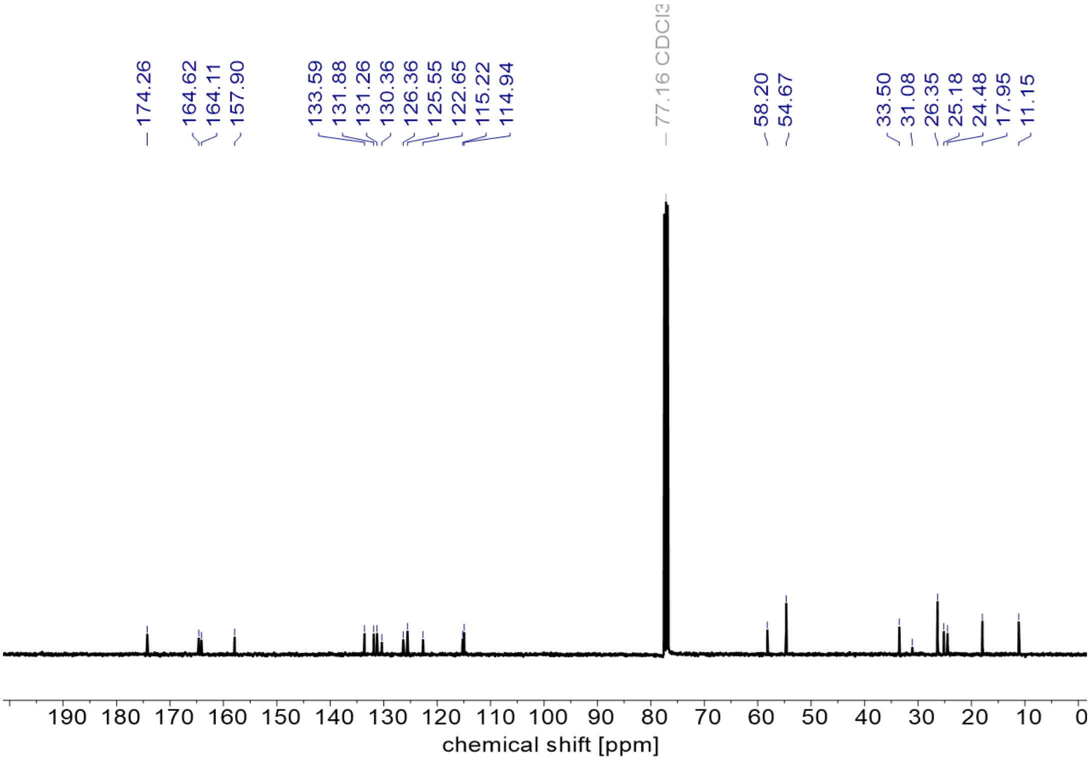

Figure S20: ^{13}C NMR spectrum ($CDCl_3$, 101 MHz, 298 K) of C_0-Nap **5**.

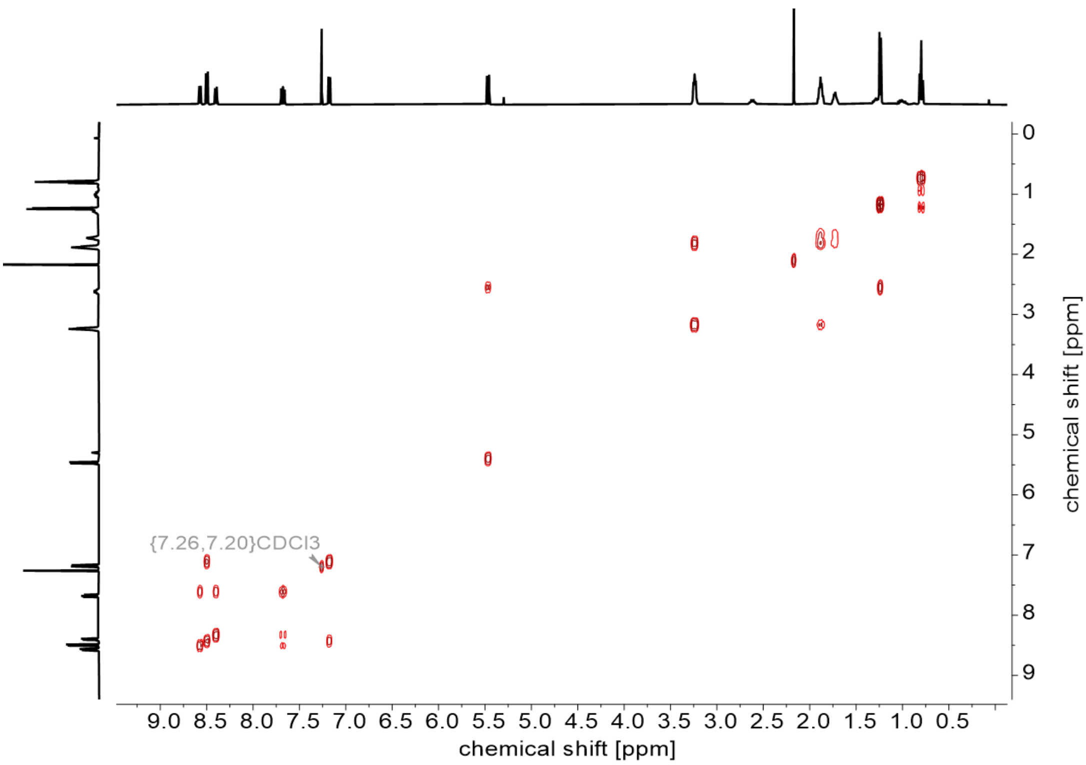

Figure S21: ^{1}H,^{1}H-COSY-NMR spectrum ($CDCl_3$, 400 MHz, 298 K) of C_0-Nap **5**.

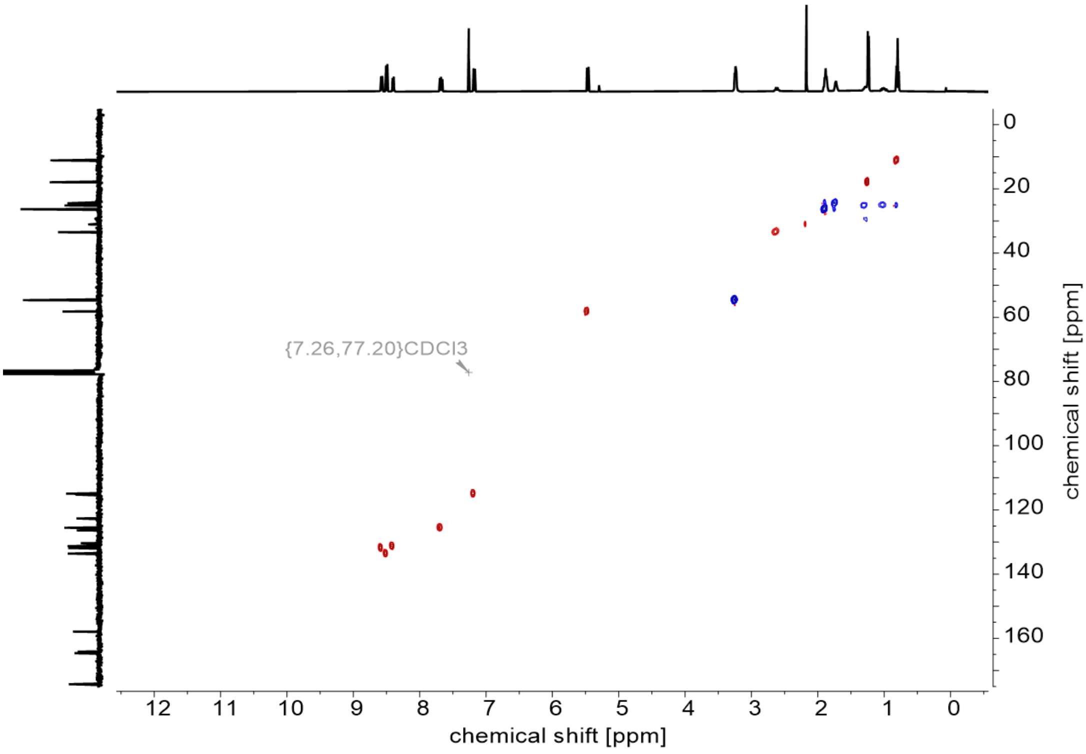

Figure S22: ^{1}H,^{13}C-HSQC-NMR spectrum ($CDCl_3$, 101 MHz, 298 K) of C_0-Nap **5**.

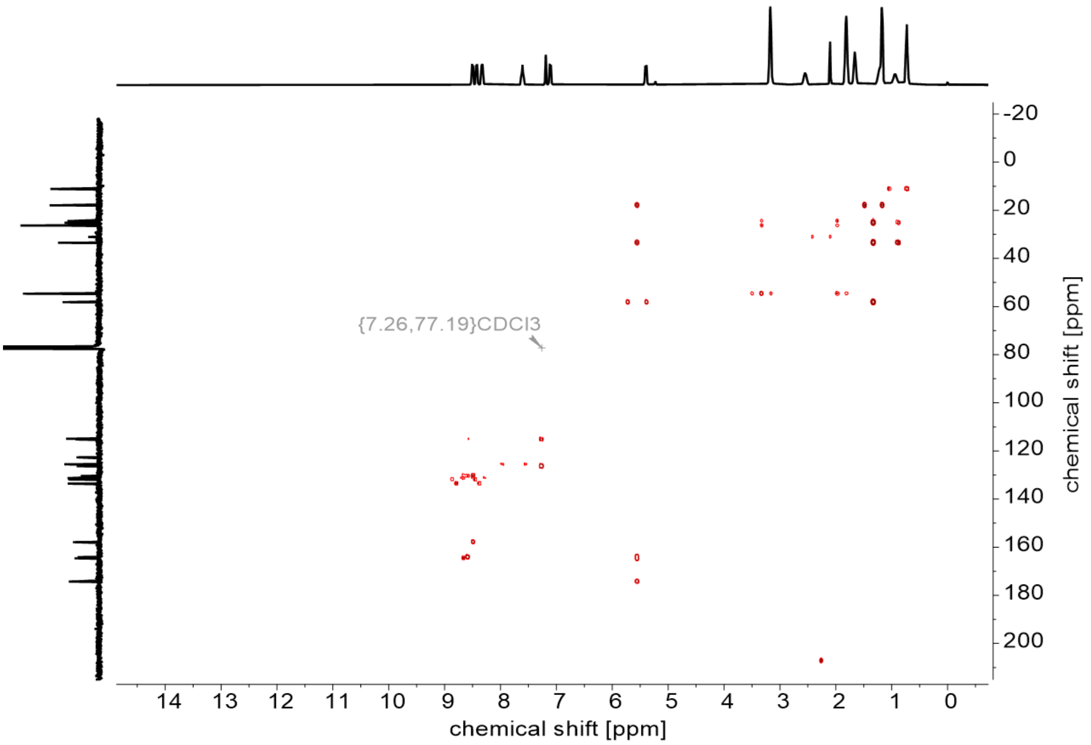

Figure S23: $^{1}H,^{13}C$-HMBC-NMR spectrum ($CDCl_3$, 101 MHz, 298 K) of C_0-Nap **5**.

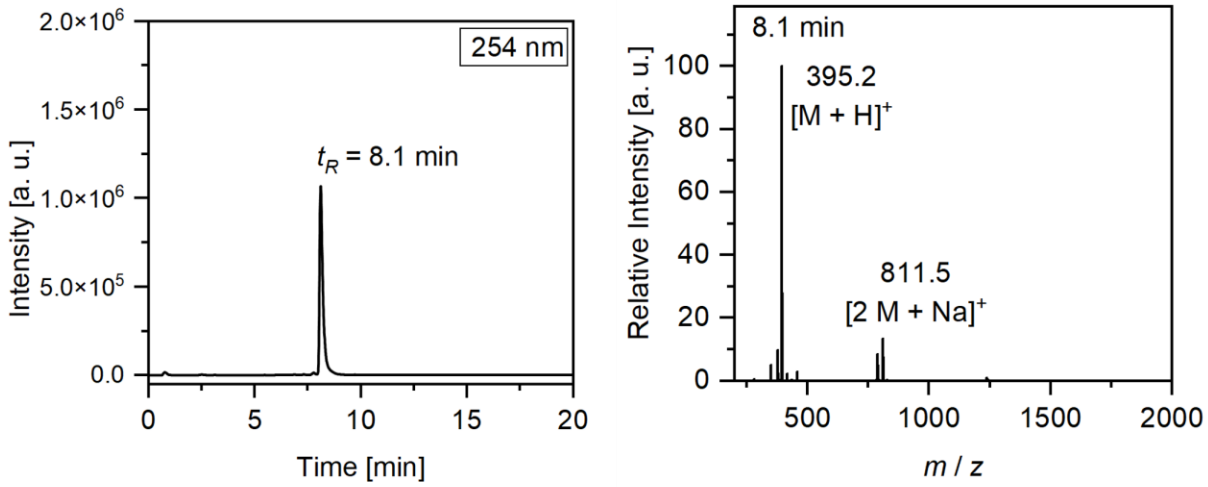

Figure S24: LC elugram (left, detection at 254 nm) and corresponding mass data (right) of C_0-Nap **5**.

2.2 Synthesis of peptides

2.2.1 Synthesis of C_6-Nap-ICA

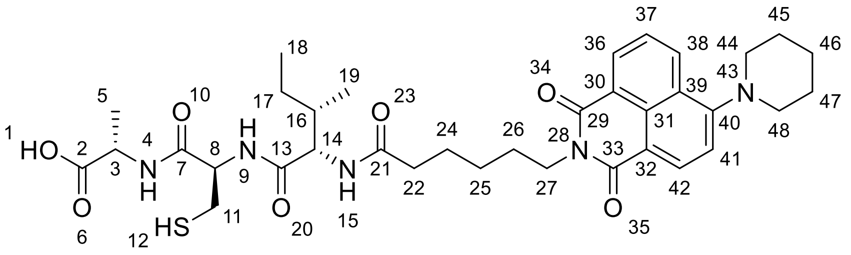

C_6-Nap-ICA **6** was synthesized using SPPS at a scale of 0.10 mmol on Fmoc-L-Ala Clt-resin according to the general procedure for peptide synthesis. After deprotection of the final Fmoc-protecting group, the H_2N-ICA-Clt resin was washed with DMF (3 × 5 mL). C_6-Nap **3** (80 mg; 0.12 mmol; 1.2 equiv), PyBOP® (155 mg; 0.30 mmol; 3.0 equiv) and *N,N'*-DIPEA (100 µL, 0.60 mmol; 6.0 equiv) were added to the resin and covered with DMF (5 mL). The reaction mixture was mixed overnight at room temperature. The mixture was filtered and washed with DMF (3 × 5 mL) and DCM (3 × 5 mL). The resin was treated with a cleavage cocktail consisting of 95% TFA, 2.5% TIPS and 2.5% H_2O for two hours at room temperature. The mixture was filtered and the resin was washed with DCM (3 × 5 mL). The solvents were removed under reduced pressure. The crude was purified using HPLC according to the general procedure. C_6-Nap-ICA **6** (25 mg; 0.037 mmol; 37%) was obtained as a yellow solid.

LC–MS: (pos.): calculated for $[M + H]^+$: *m/z* = 682.9; measured: *m/z* = 682.4; calculated for $[M + Na]^+$: *m/z* = 704.9; measured: *m/z* = 704.4; calculated for $[2 M + Na]^+$: *m/z* = 1386.7; measured: *m/z* = 1385.9.

^{1}H NMR, COSY (400 MHz, DMSO-d_6, 298 K): δ [ppm] = 12.61 (s, 1H, H_1); 8.47 (dd, 1H, J = 7.3 Hz, 1.2 Hz, H_{38}); 8.43–8.36 (m, 2H, $H_{36,42}$); 8.15 (d, 1H, J = 7.1 Hz, H_9); 7.98 (d, 1H, J = 7.9 Hz, H_4); 7.87 (d, 1H, J = 8.4 Hz, H_{15}); 7.81 (dd, 1H, J = 8.5 Hz, 7.3 Hz, H_{37}); 7.31 (d, 1H, J = 8.2 Hz, H_{41}); 4.43 (td, 1H, J = 7.7 Hz, 5.1 Hz, H_8); 4.16 (dt, 2H, J = 13.8 Hz, 7.5 Hz, $H_{3,14}$); 4.01 (t, 2H, J = 7.4 Hz, H_{27}); 3.20 (t, 4H, J = 5.3 Hz, $H_{44,48}$); 2.84–2.63 (m, 2H, H_{11}); 2.26 (dd, 1H, J = 9.0 Hz, 7.9 Hz, H_{16}); 2.41 (qd, 1H, J = 14.3 Hz, 7.1 Hz, H_{22}); 1.82 (p, 4H, J = 5.6 Hz, $H_{45,47}$); 1.75–1.45 (m, 8H, $H_{24,25,26,46}$); 1.03–0.88 (m, 2H, H_{17}); 0.81–0.72 (m, 6H, $H_{18,19}$).

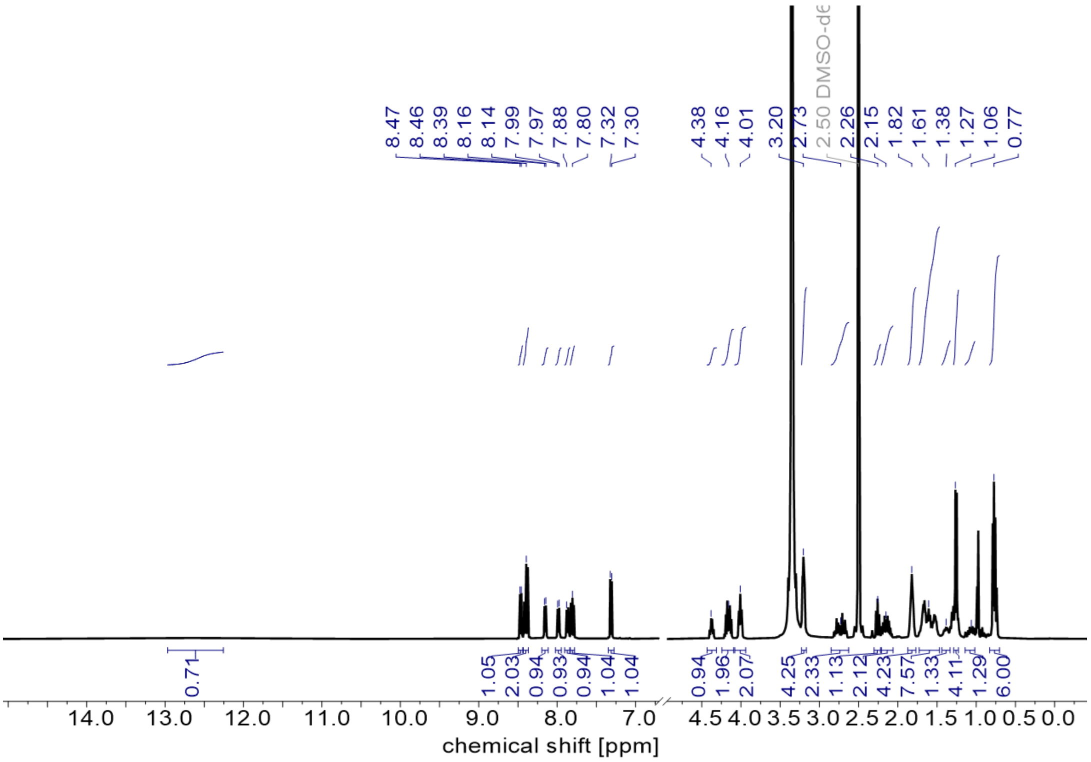

Figure S25: ^{1}H NMR spectrum (DMSO-d_6, 400 MHz, 298 K) of C_6-Nap-ICA **6**.

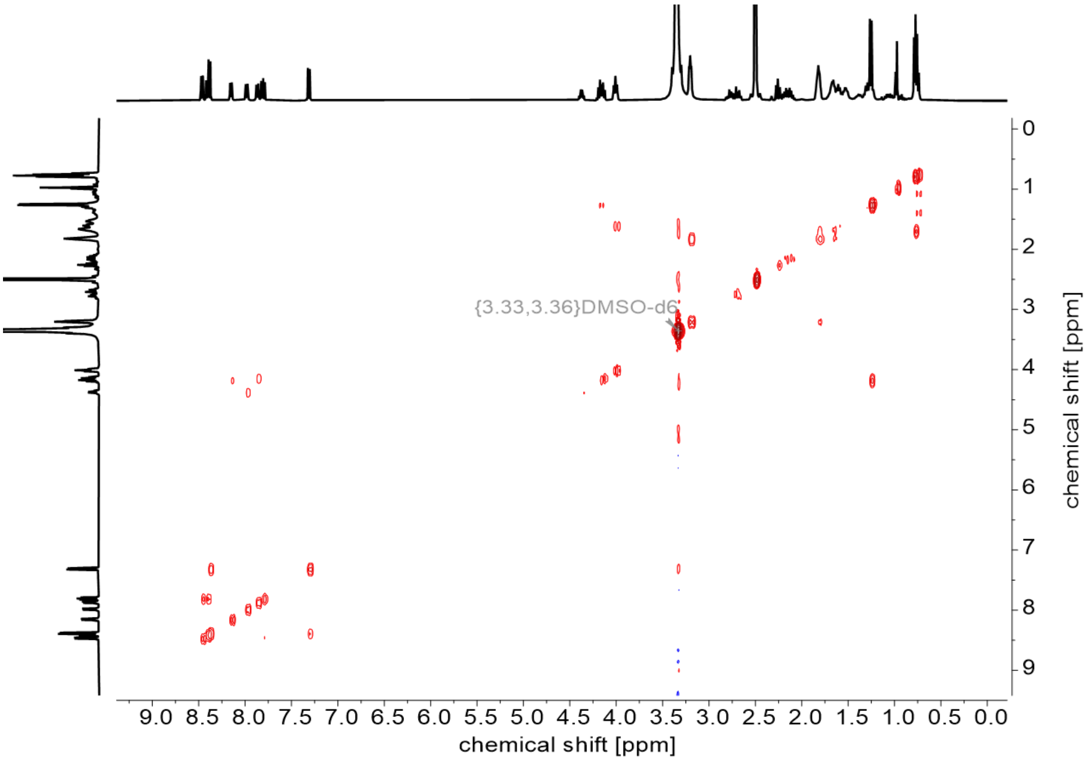

Figure S26: ^{1}H,^{1}H-COSY-NMR spectrum (DMSO-d_6, 400 MHz, 298 K) of C_6-Nap-ICA **6**.

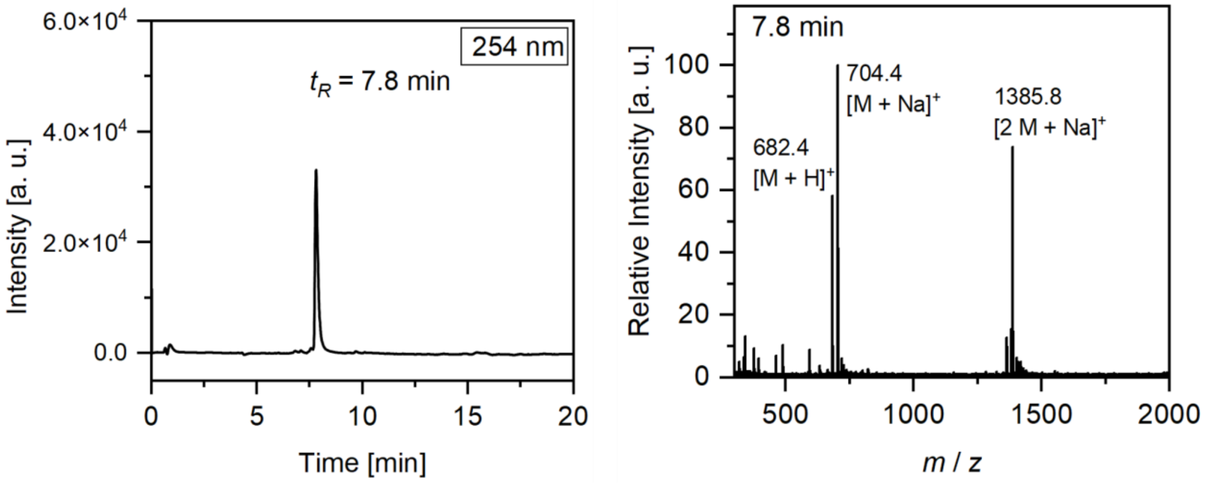

Figure S27: LC elugram (left, detection at 254 nm) and corresponding mass data (right) of C_6-Nap-ICA **6**.

2.2.2 Synthesis of C_3-Nap-ICA

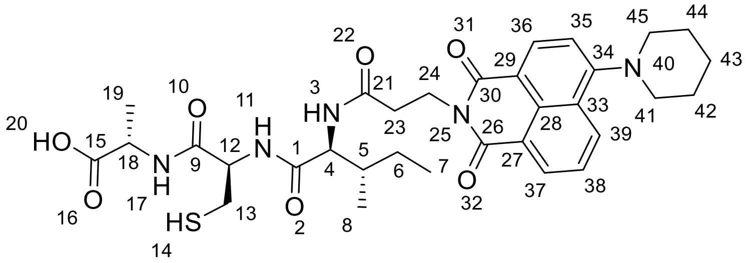

C_3-Nap-ICA **7** was synthesized using SPPS at a scale of 0.10 mmol on Fmoc-L-Ala Clt-resin according to the general procedure for automated peptide synthesis. After deprotection of the final Fmoc-protecting group, the H_2N-ICA-Clt resin was washed with DMF (3 × 5 mL). C_3-Nap **4** (75 mg; 0.12 mmol; 1.2 equiv), PyBOP® (155 mg; 0.30 mmol; 3.0 equiv) and *N,N'*-DIPEA (100 µL, 0.60 mmol; 6.0 equiv) were added to the resin and covered with DMF (5 mL). The reaction mixture was mixed overnight at room temperature. The mixture was filtered and washed with DMF (3 × 5 mL) and DCM (3 × 5 mL). Then, the resin was treated with a cleavage cocktail consisting of 95% TFA, 2.5% TIPS and 2.5% H_2O for two hours at room temperature. The mixture was filtered and the resin was washed with DCM (3 × 5 mL). The solvents were removed under reduced pressure. A yellow solid crude was obtained and purified via HPLC according to the general procedure. C_3-Nap-ICA **7** (19 mg; 0.03 mmol; 30%) was obtained as an orange solid.

LC–MS: (pos.): calculated for $[M + H]^+$: m/z = 640.8; measured: m/z = 640.3; calculated for $[M + Na]^+$: m/z = 662.8; measured: m/z = 662.3; calculated for $[2 M + H]^+$: m/z = 1280.5; measured: m/z = 1279.8; calculated for $[2 M + Na]^+$: m/z = 1302.5; measured: m/z = 1301.8.

^{1}H NMR, COSY (700 MHz, DMSO-d_6, 298 K): δ [ppm] = 12.60 (s, 1H, H_{20}); 8.47 (dd, 1H, J = 7.3 Hz, 1.2 Hz, H_{39}); 8.42 (dd, 1H, J = 8.5 Hz, 1.2 Hz, H_{37}); 8.38 (d, 1H, J = 8.1 Hz, H_{36}); 8.13 (d, 1H, J = 7.1 Hz, H_{11}); 8.07 (d, 1H, J = 8.1 Hz, H_{17}); 8.04 (d, 1H, J = 8.0 Hz, H_3); 7.81 (dd, 1H, J = 8.4 Hz, 7.2 Hz, H_{38}); 7.32 (d, 1H, J = 8.1 Hz, H_{35}); 4.39 (td, 1H, J = 7.9 Hz, 5.0 Hz, H_{12}); 4.27 (ddd, 1H, J = 13.0 Hz, 8.8 Hz, 6.6 Hz, H_4); 4.23–4.18 (m, 1H, H_{18}); 4.14 (dt, 2H, J = 17.0 Hz, 7.5 Hz, H_{24}); 3.21 (s, 4H, $H_{41,45}$); 2.85–2.79 (m, 1H, $H_{13'}$); 2.75–2.68 (m, 1H, $H_{13''}$); 2.60–2.51 (m, 2H, H_{23}); 2.30 (t, 1H, J = 8.5 Hz, H_5); 1.82 (p, 4H, J = 5.6 Hz, $H_{42,44}$); 1.66 (s, 2H, H_{43}); 1.41–1.35 (m, 1H, $H_{6'}$); 1.26 (d, 3H, J = 7.3 Hz, H_{19}); 1.07–0.99 (m, 1H, $H_{6''}$); 0.79 (d, 3H, J = 6.9 Hz, H_8); 0.73 (t, 3H, J = 7.4 Hz, H_7).

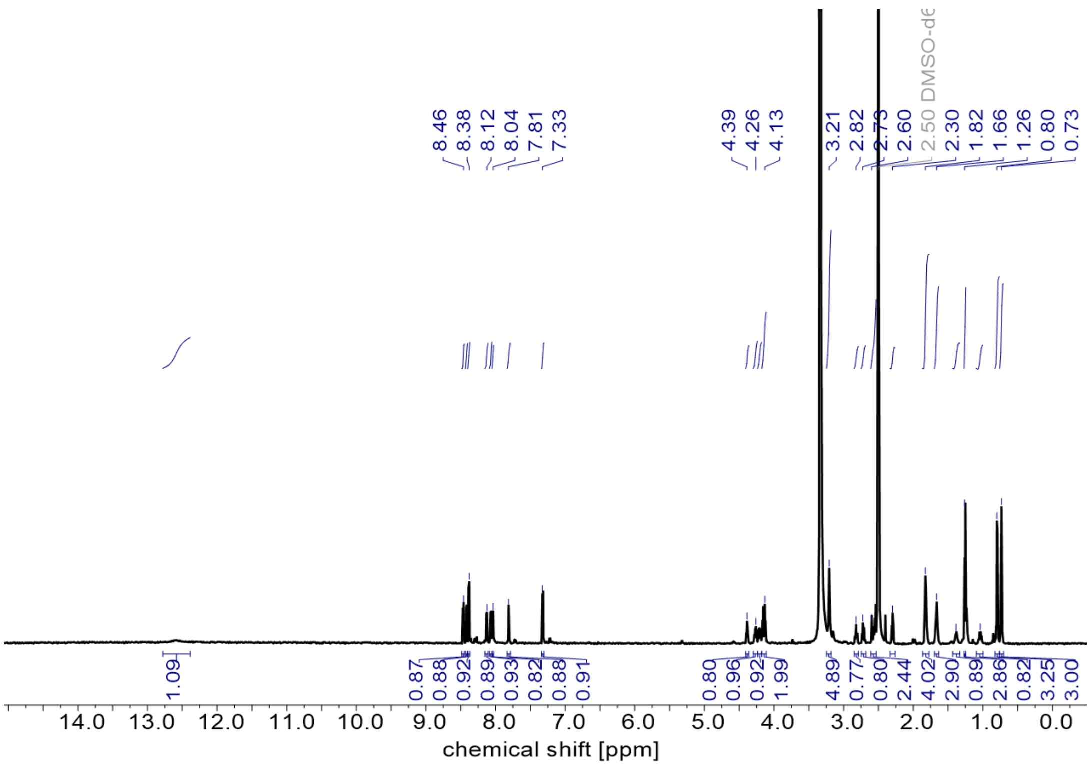

Figure S28: ^{1}H NMR spectrum (DMSO-d_6, 700 MHz, 298 K) of C$_3$-Nap-ICA **7**.

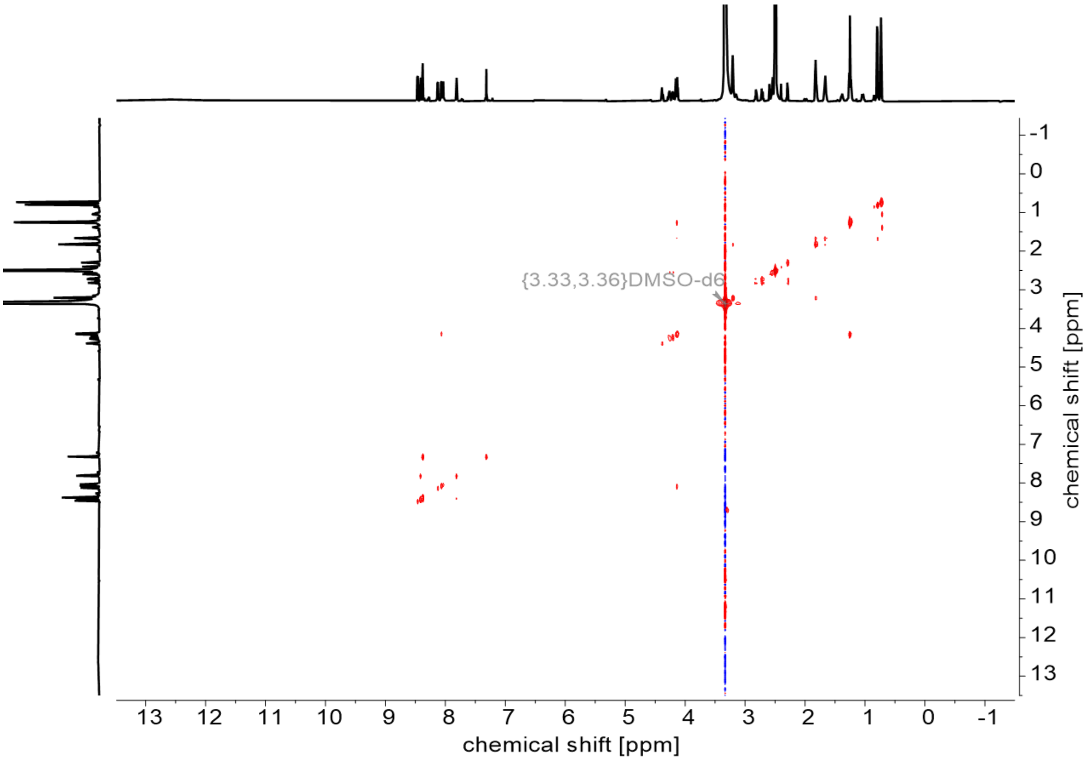

Figure S29: ^{1}H,^{1}H-COSY-NMR spectrum (DMSO-d_6, 700 MHz, 298 K) of C_3-Nap-ICA **7**.

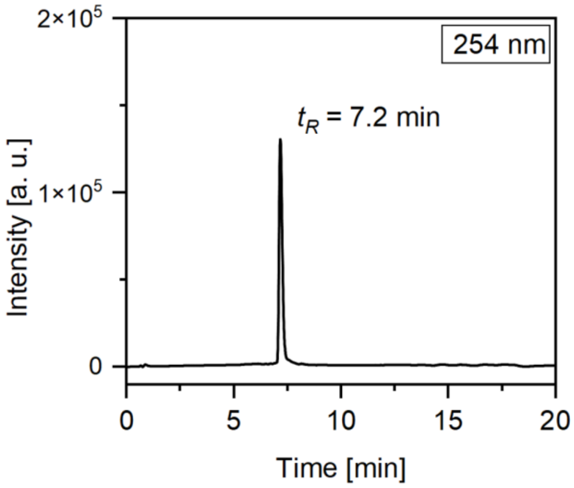

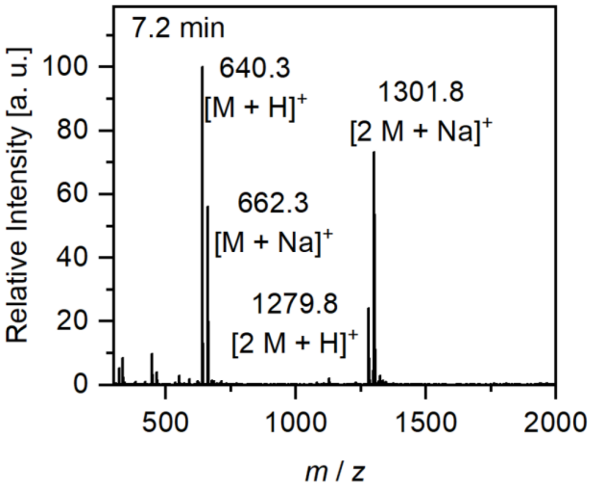

Figure S30: LC elugram (left, detection at 254 nm) and corresponding mass data (right) of C_3-Nap-ICA **7**.

2.2.3 Synthesis of C_0-Nap-ICA

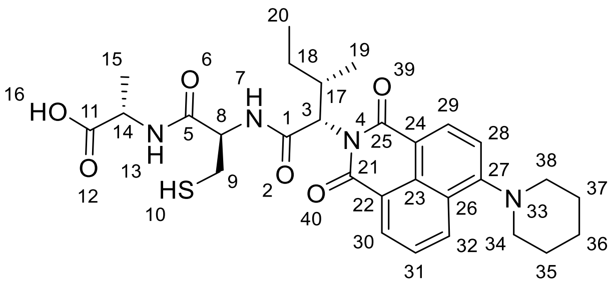

C_0-Nap-ICA **8** was synthesized using SPPS at a scale of 0.10 mmol on Fmoc-L-Ala Clt-resin according to the general procedure for automated peptide synthesis. After deprotection of the final Fmoc-protecting group, the H_2N-CA-Clt resin was washed with DMF (3 × 5 mL). C_0-Nap **8** (70 mg; 0.12 mmol; 1.2 equiv), PyBOP® (155 mg; 0.30 mmol; 3.0 equiv), *N,N'*-DIPEA (100 µL; 0.60 mmol; 6.0 equiv) were dissolved in DMF (5 mL) and added to the resin. The mixture was mixed overnight at room temperature. After filtration, the mixture was washed with DMF (3 × 5 mL) and DCM (3 × 5 mL). The resin was treated with a cleavage cocktail consisting of 95% TFA, 2.5% TIPS and 2.5% H_2O for two hours. The mixture was filtered and washed with DCM (3 × 5 mL). The solvents were removed under reduced pressure. A yellow solid crude was obtained and purified via HPLC according to the general procedure. C_0-Nap-ICA **8** (14 mg; 0.025 mmol; 25%) was obtained as a yellow solid.

LC MS: (pos.): calculated for $[M + H]^+$: *m/z* = 569.7; measured: *m/z* = 569.3; calculated for $[2 M + H]^+$: *m/z* = 1138.4; measured: *m/z* = 1137.7.

^{1}H NMR, COSY (700 MHz, DMSO-d_6, 298 K): δ [ppm] = 12.53 (s, 1H, H_{16}); 8.47 (d, 1H, J = 7.6 Hz, H_{32}); 8.44 (dd, 1H, J = 7.2 Hz, 1.2 Hz, H_{30}); 8.38 (d, 1H, J = 7.9 Hz, H_{29}); 8.19 (d, 1H, J = 7.1 Hz, H_7); 8.05 (d, 1H, J = 8.1 Hz, H_{13}); 7.83 (ddd, 1H, J = 14.0 Hz, 8.4 Hz, 7.2 Hz, H_{28}); 7.34 (dd, 1H, J = 14.6 Hz, 8.2 Hz, H_{31}); 5.15 (d, 1H, J = 9.2 Hz, H_3); 4.40 (qd, 1H, J = 8.6 Hz, 4.7 Hz, H_8); 4.15 (p, 1H, J = 7.3 Hz, H_{14}); 3.22 (t, 4H, J = 5.4 Hz, $H_{34,38}$); 2.70–2.57 (m, 2H, $H_{9',9''}$); 2.02–1.96 (m, 1H, H_{17}); 1.83 (p, 4H, J = 5.6 Hz, $H_{35,37}$); 1.70–1.64 (m, 2H, H_{36}); 1.29 (d, 2H, J = 7.3 Hz, H_{18}); 1.11 (d, 3H, J = 6.4 Hz, H_{15}); 0.97 – 0.87 (m, 3H, H_{19}); 0.72 (t, 3H, J = 7.4 Hz, H_{20}).

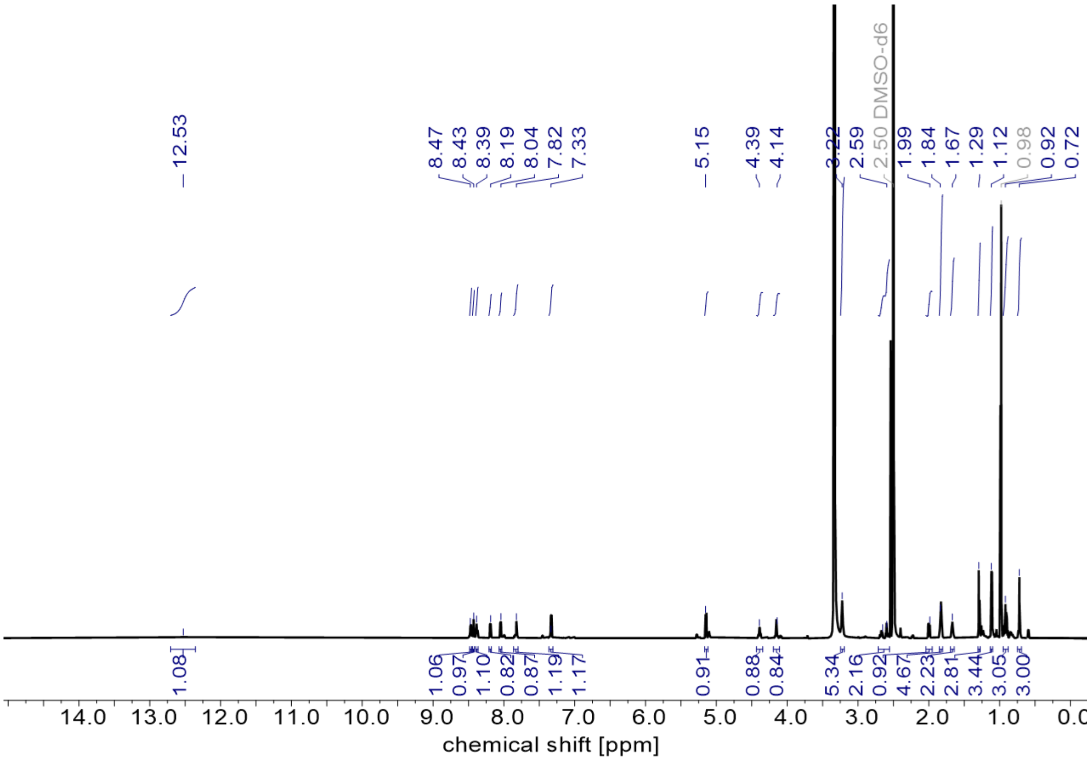

Figure S31: ^{1}H NMR spectrum (DMSO-d_6, 700 MHz, 298 K) of C_0-Nap-ICA **8**.

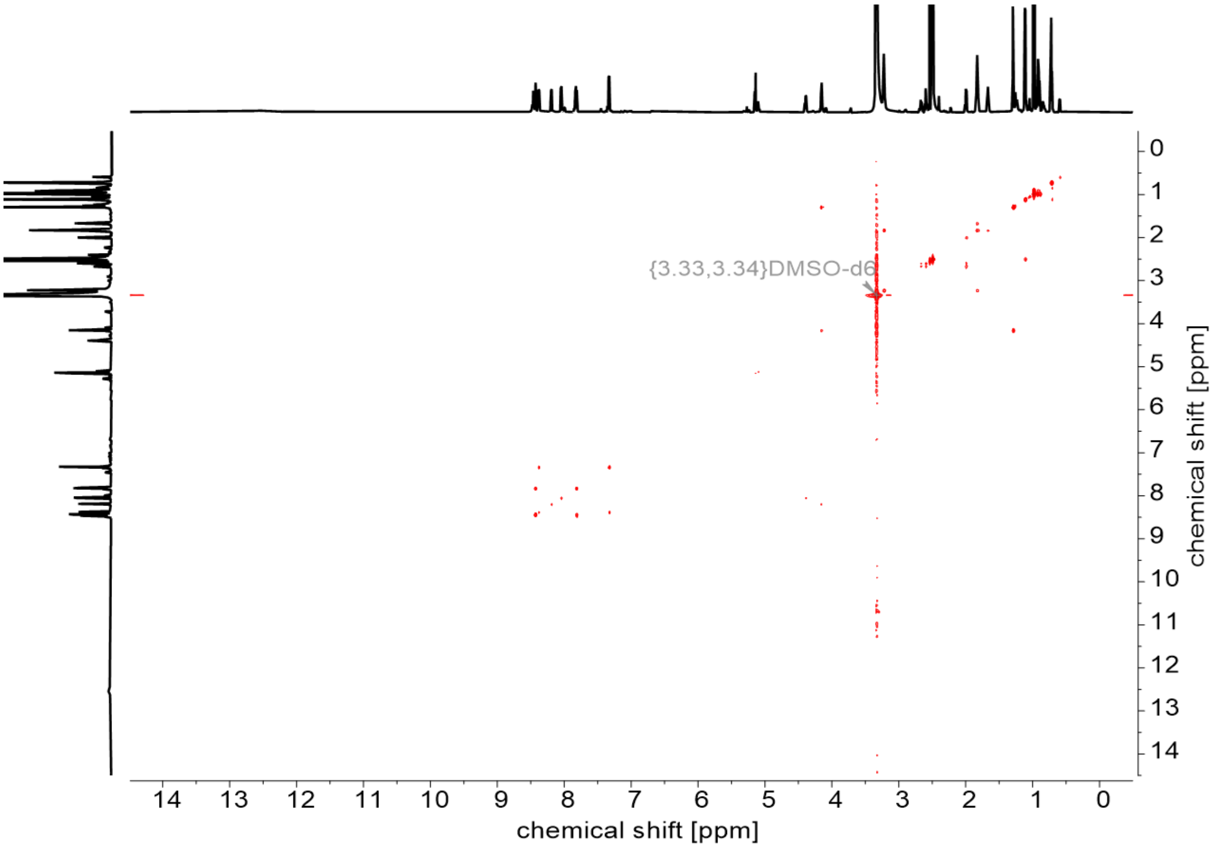

Figure S32: ^{1}H,^{1}H-COSY-NMR spectrum (DMSO-d_6, 700 MHz, 298 K) of C_0-Nap-ICA **8**.

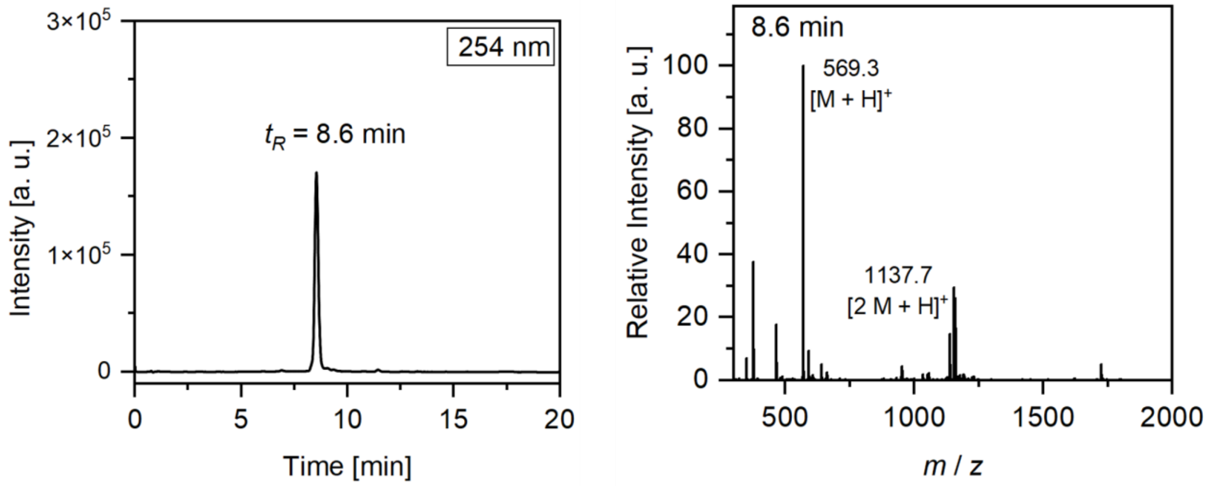

Figure S33: LC elugram (left, detection at 254 nm) and corresponding mass data (right) of C_0-Nap-ICA **8**.

3 Self-assembly

3.1 Assembly conversion rate

The peptides were analyzed according to the general procedure for assembly conversion rate determination. The elugrams were referenced to the standard and plotted using Origin Pro by OriginLab®. The area under curve (AUC) of the peptide signals in PBS (AUC_{Buffer}) and in MeOH ($AUC_{Organic\ Solvent}$) were calculated and used to determine the conversion rate (CR) using the following equation S1:

$$CR\ [\%] = (1 - \frac{AUC_{Buffer}}{AUC_{Organic\ Solvent}}) \cdot 100\ \% \qquad \text{(equation S1)}$$

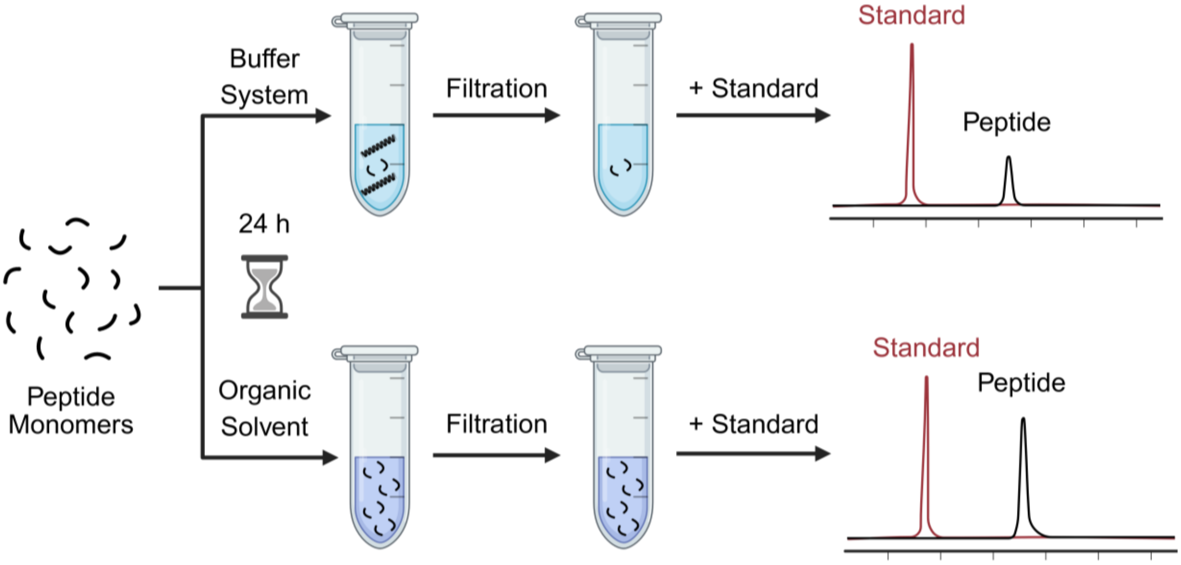

Figure S34: Scheme describing the procedure of the assembly conversion assay. Peptide monomers (10 mM in TFE) are added to an aqueous buffer system and an organic solvent, where the last-mentioned serves as a control in which peptide monomers do not assemble. The samples were incubated for 24 h. Aggregated peptides in the aqueous buffer system were removed through filtration. The peptide monomers in the filtrate were analyzed regarding their elution time and absorbance using analytical HPLC in both systems. The internal standard was added to normalize the absorbance. The differences in absorbance of the peptide monomers between the samples allows to determine the assembly conversion using equation S1. Buffer system: PBS buffer (50 mM, pH 7.4, 1% TFE), organic solvent: MeOH (1% TFE). Created in BioRender. Lahu, A. (2026) https://BioRender.com/bvkpq0n. This content is not subject to CC BY 4.0.

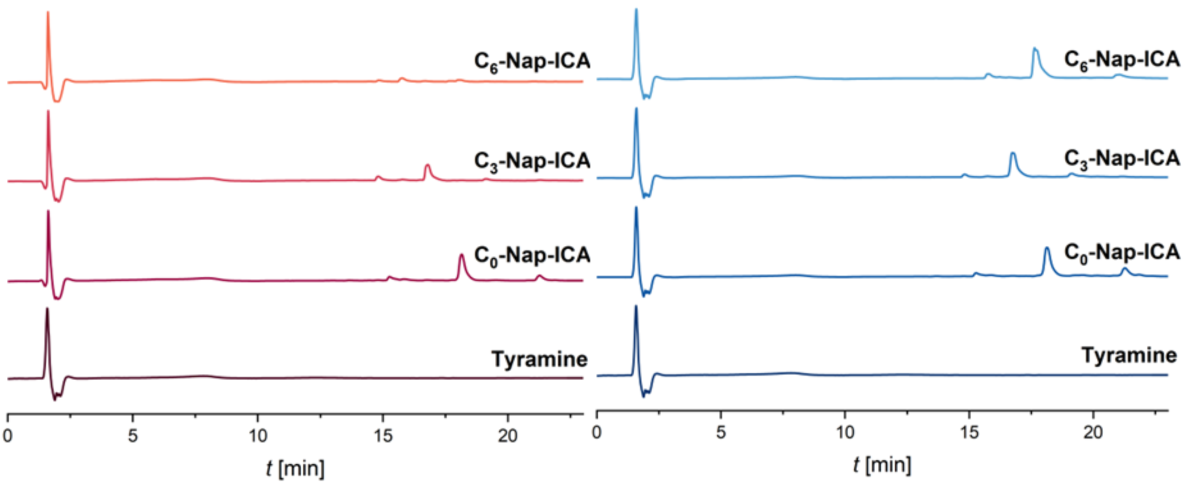

Figure S35: Elugrams of C_6-Nap-ICA **6**, C_3-Nap-ICA **7** and C_0-Nap-ICA **8** (all 100 µM) in PBS (50 mM, pH 7.4, 1% TFE; *left*) and MeOH (1% TFE; *right*) as well as the internal standard tyramine (200 µM in MeOH, **Standard**). The elugrams are shown as a plot of absorbance intensity at 214 nm against retention time t_R.

3.2 Thioflavin T (ThT) assay

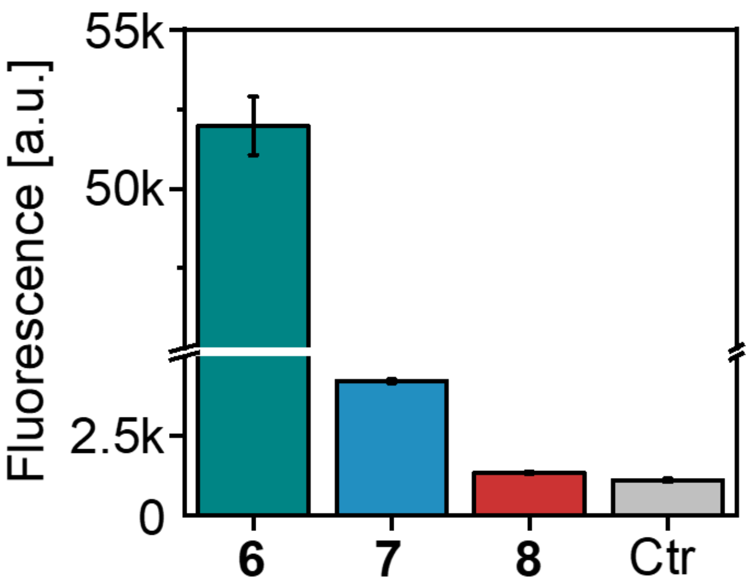

Figure S36: Bar plot showing the fluorescence intensity of thioflavin T at λ_{em} = 482 nm (λ_{ex} = 450 nm) after incubation with C_6-Nap-ICA **6**, C_3-Nap-ICA **7** or C_0-Nap-ICA **8** (all 100 µM) in PBS (50 mM, pH 7.4, 1% TFE). Control shows the fluorescence of Thioflavin T in PBS (50 mM, pH 7.4, 1% TFE).

3.3 Fluorescence microscopy, transmission electron and atomic force microscopy

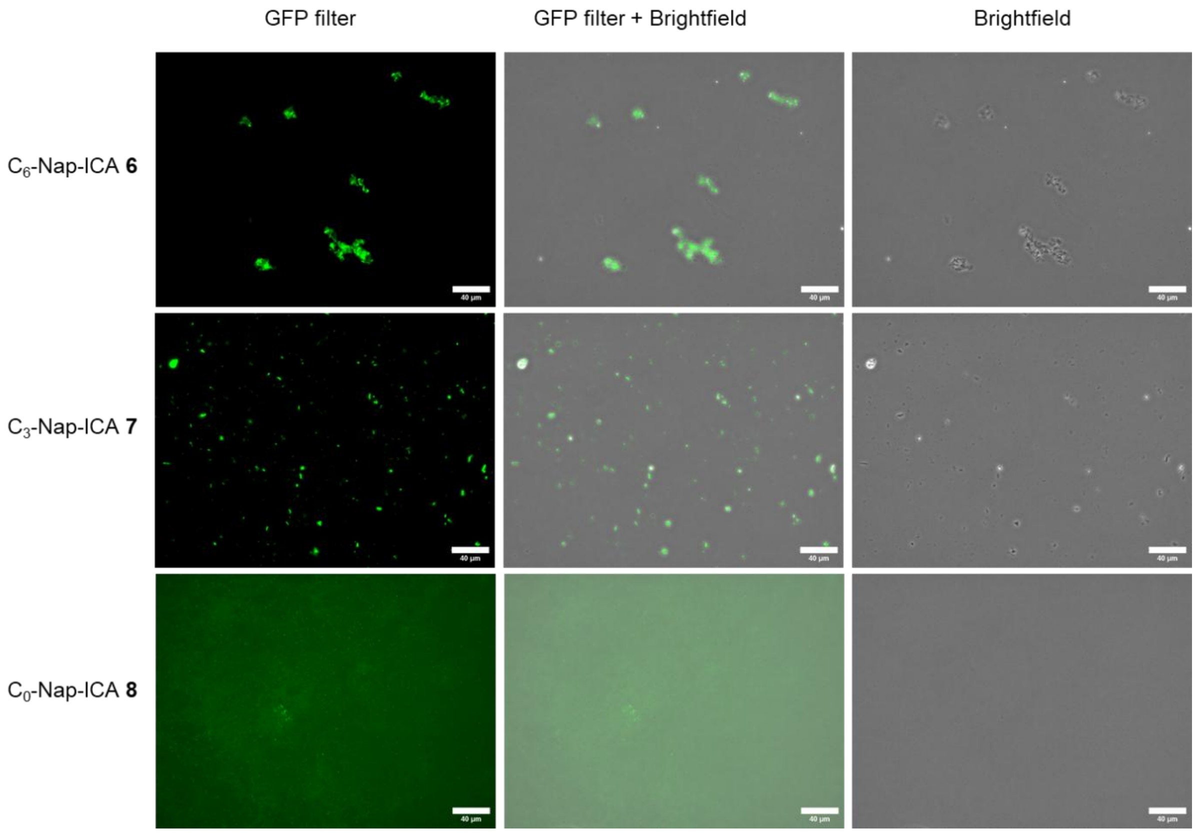

Figure S37: Fluorescence microscopy micrographs of C_6-Nap-ICA **6**, C_3-Nap-ICA **7** or C_0-Nap-ICA **8** (all 100 µM) in PBS (50 mM, pH 7.4, 1% TFE) with green fluorescence filter (λ_{ex} = 470/40 nm, λ_{em} = 525/50 nm) and brightfield, scale bar = 40 µm.

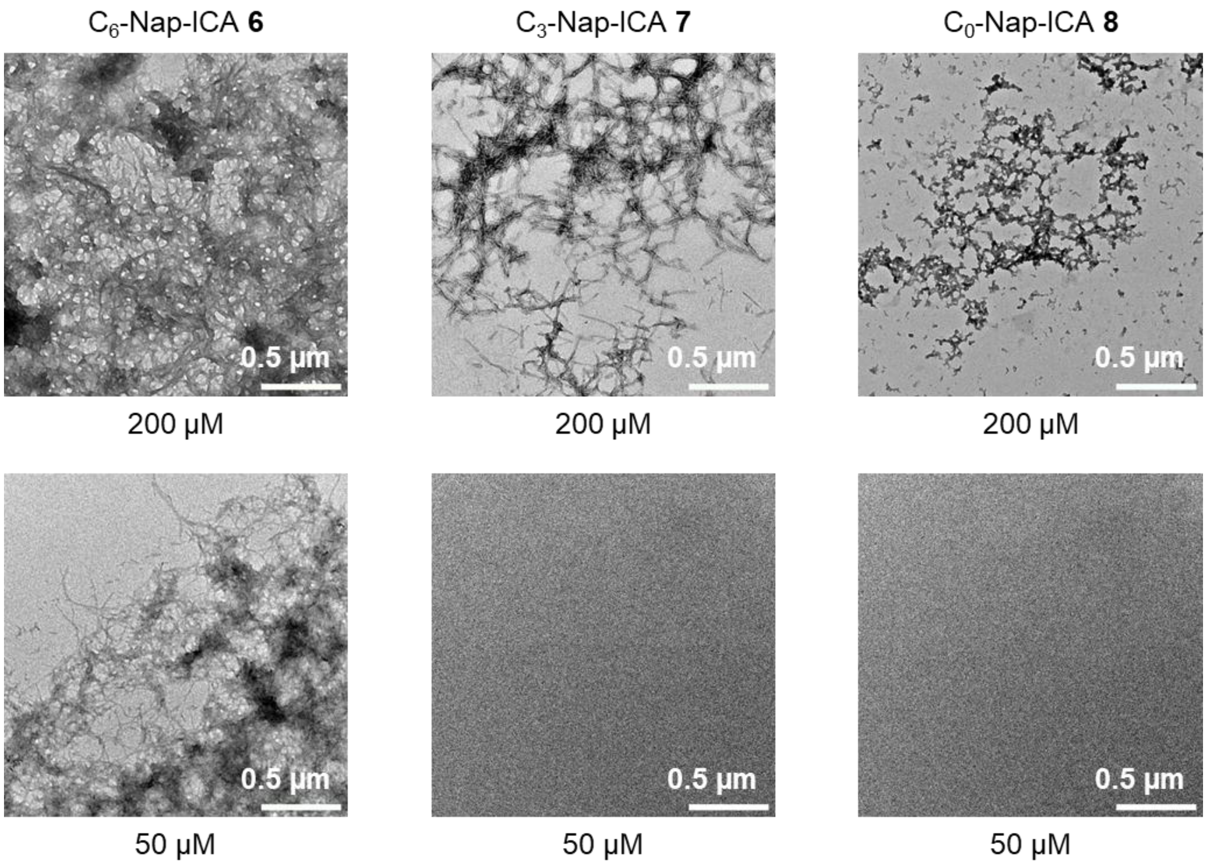

Figure S38: TEM micrographs of C_6-Nap-ICA **6**, C_3-Nap-ICA **7** and C_0-Nap-ICA **8** (200/50 µM) in PBS (50 mM, pH 7.4, 1% TFE) after 24 h incubation at room temperature. The structures were stained with uranyl(IV) acetate (4%), scale bar = 0.5 µm.

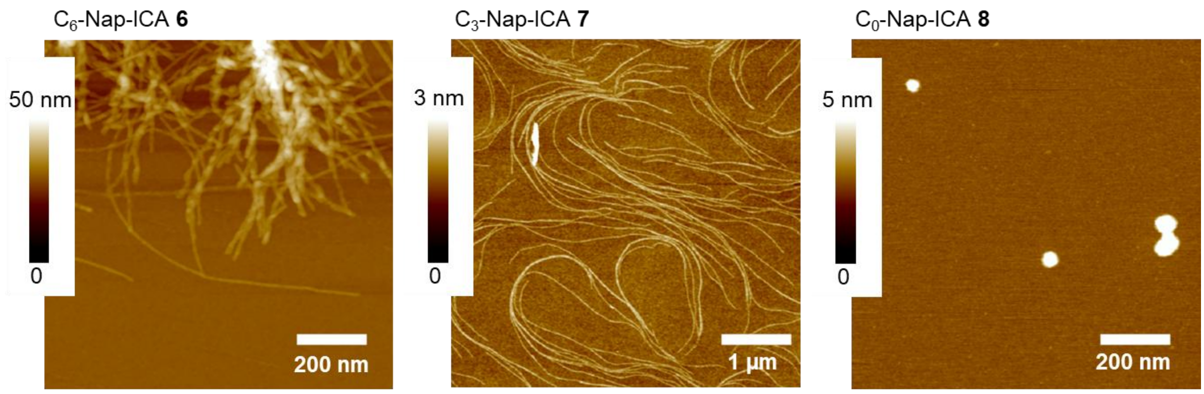

Figure S39: AFM images of supramolecular structures formed by C_6-Nap-ICA **6**, C_3-Nap-ICA **7**, C_0-Nap-ICA **8** samples at 100 µM in MilliQ-H_2O (1% TFE) after 24 h incubation at room temperature.

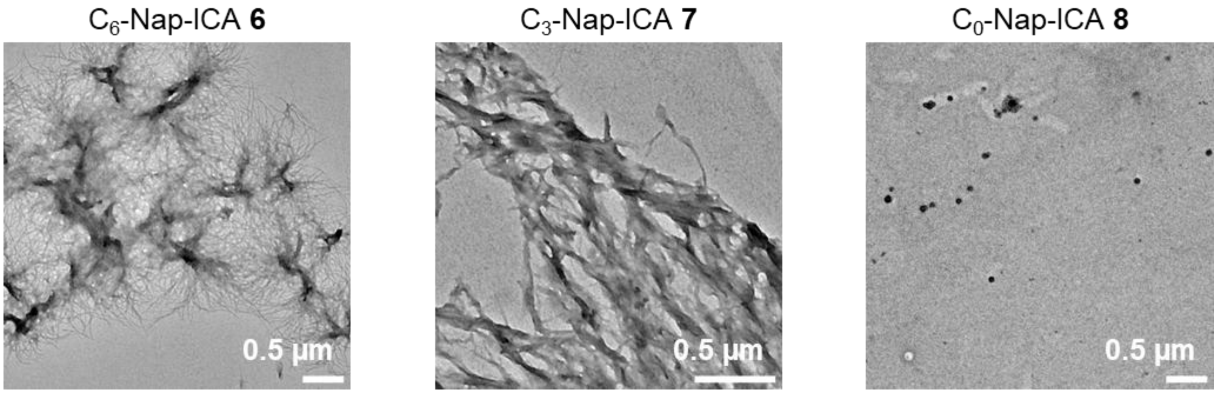

Figure S40: TEM micrographs of C_6-Nap-ICA **6**, C_3-Nap-ICA **7** and C_0-Nap-ICA **8** (100 µM) in MilliQ-H_2O (1% TFE) after 24 h incubation at room temperature. The structures were stained with uranyl(IV) acetate (4%), scale bar = 0.5 µm.

3.4 Circular dichroism (CD) spectroscopy

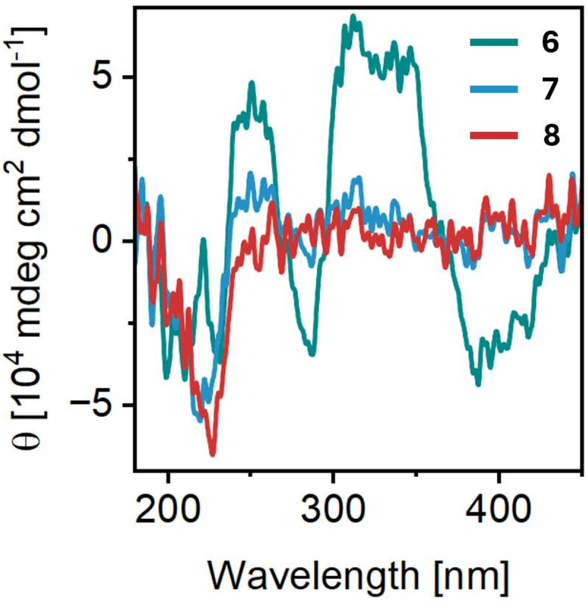

Figure S41: Circular dichroism spectra of C_6-Nap-ICA **6**, C_3-Nap-ICA **7** and C_0-Nap-ICA **8** (all 100 µM) in PBS (50 mM, pH 7.4, 1% TFE). The molar ellipticity is plotted against the wavelength.

4 NMR study with increasing TFE content

To investigate the self-assembly propensity of the three peptide conjugates, the compounds were dissolved in mixtures of trifluoroethanol-d_3 and PBS-buffered D_2O with increasing TFE-d_3 content (1:99, 10:90, 20:80, 30:70, 40:60, 50:50, and 100:0, *v*/*v*) and analyzed by ^{1}H NMR spectroscopy.

The aqueous component of the solvent mixtures was prepared by lyophilizing 40 mL of commercial PBS to dryness and redissolving the residue in the same volume of D_2O. Dimethyl sulfone (DMS, 6.96 mg) was added as an internal reference. Each peptide conjugate was initially dissolved in TFE-d_3, followed by the addition of the DMS-containing PBS/D_2O buffer (250 µL) and additional PBS/D_2O buffer without DMS to achieve the desired solvent compositions. The exact volumes used are summarized in Table S1.

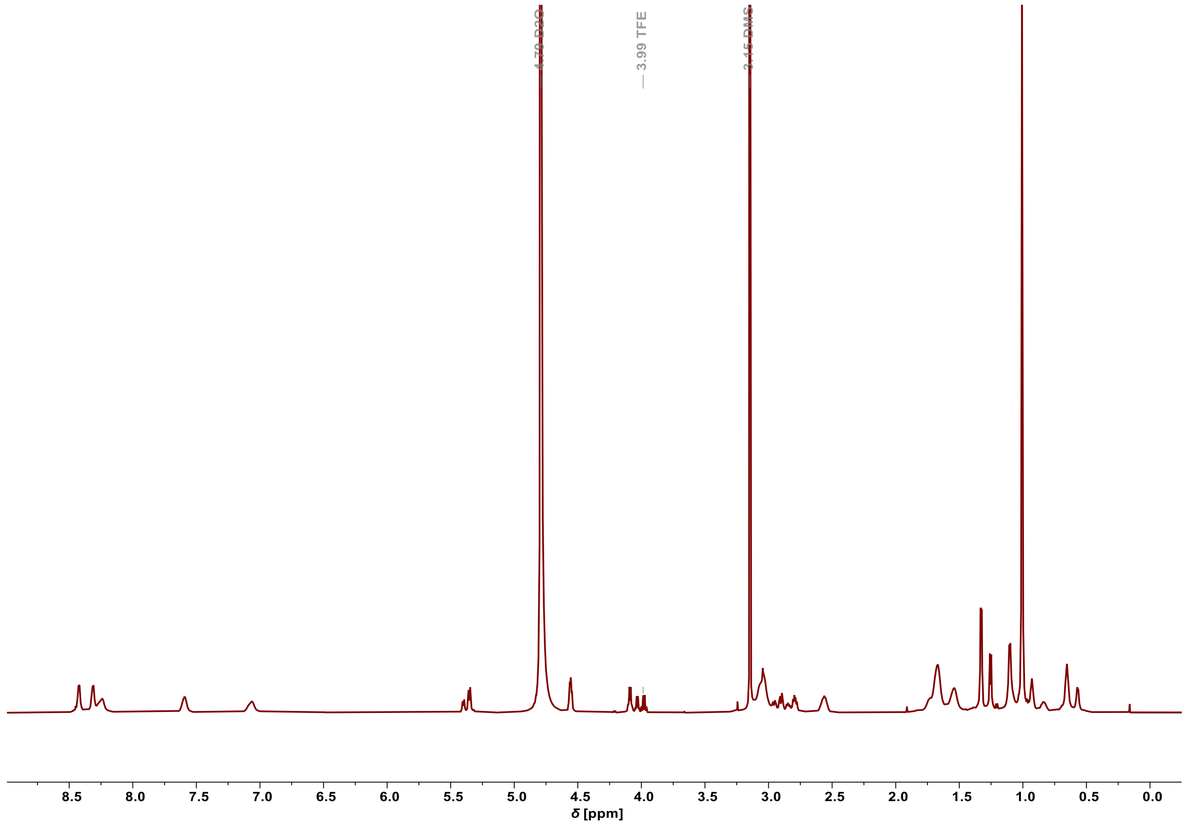

Figure S42: ^{1}H NMR (700 MHz, 298 K, D_2O/TFE-d_3 99:1) spectrum of C_0-Nap-ICA **8**.

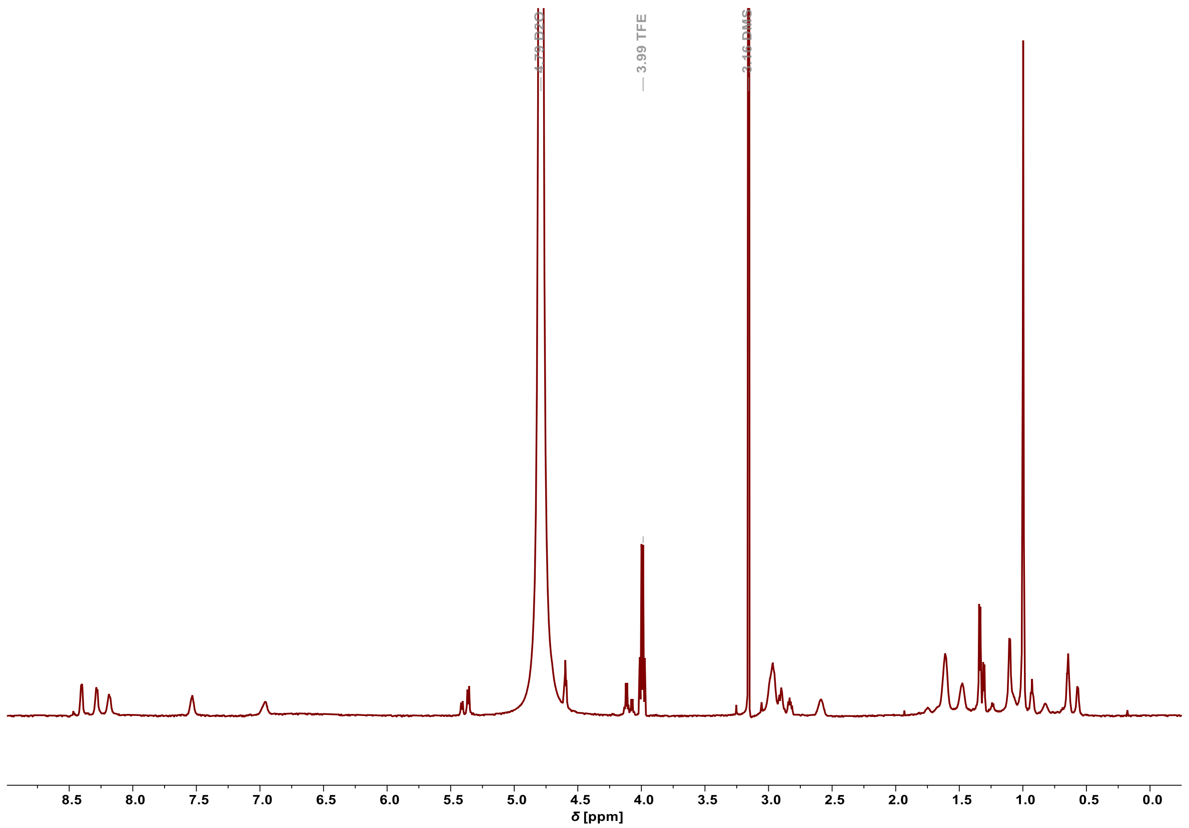

Figure S43: ^{1}H NMR (700 MHz, 298 K, D_2O/TFE-d_3 90:10) spectrum of C_0-Nap-ICA **8**.

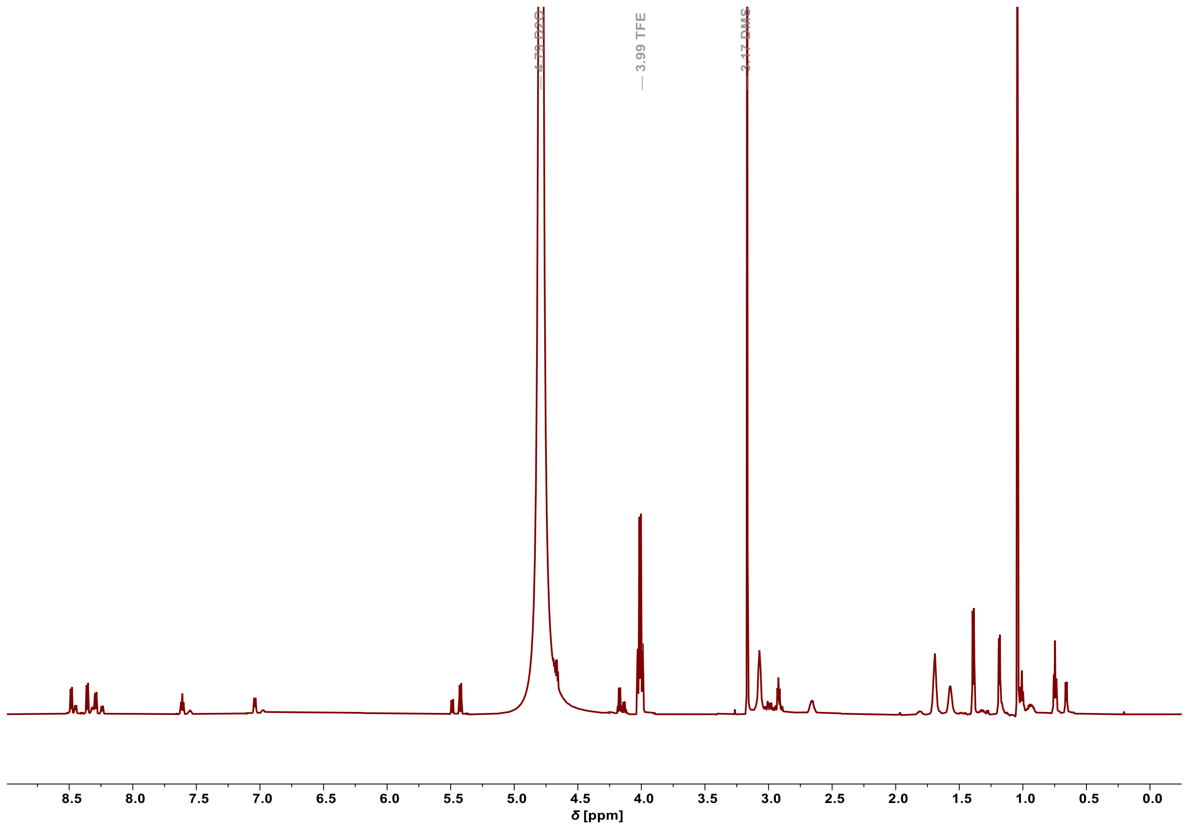

Figure S44: ^{1}H NMR (700 MHz, 298 K, D_2O/TFE-d_3 80:20) spectrum of C_0-Nap-ICA **8**.

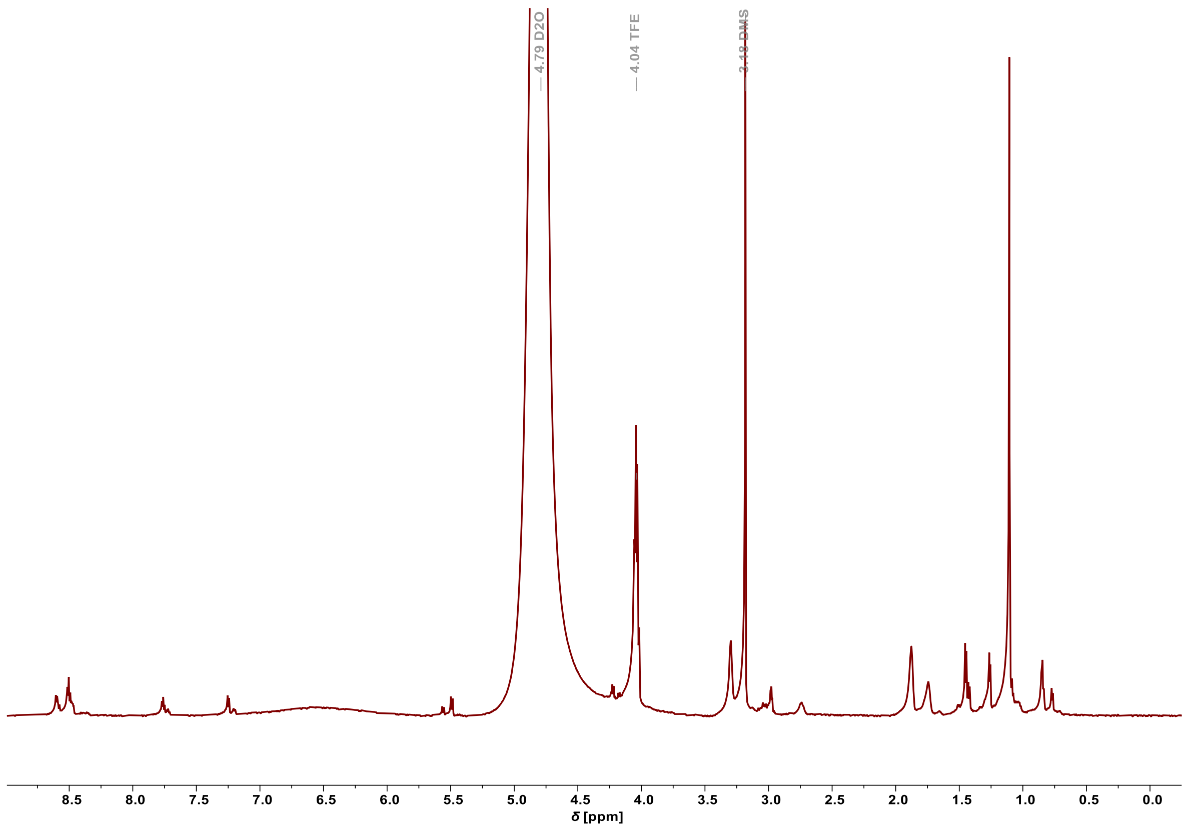

Figure S45: ^{1}H NMR (700 MHz, 298 K, D_2O/TFE-d_3 70:30) spectrum of C_0-Nap-ICA **8**.

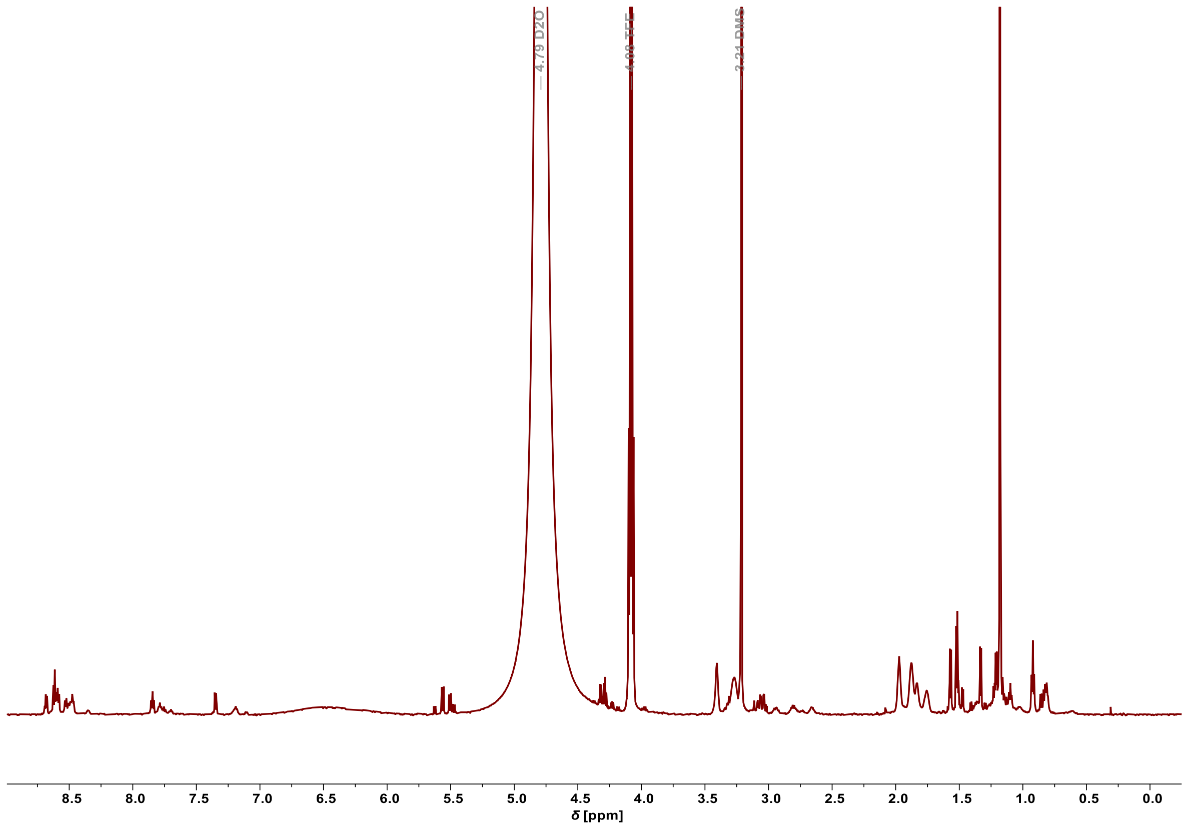

Figure S46: ^{1}H NMR (700 MHz, 298 K, D_2O/TFE-d_3 60:40) spectrum of C_0-Nap-ICA **8**.

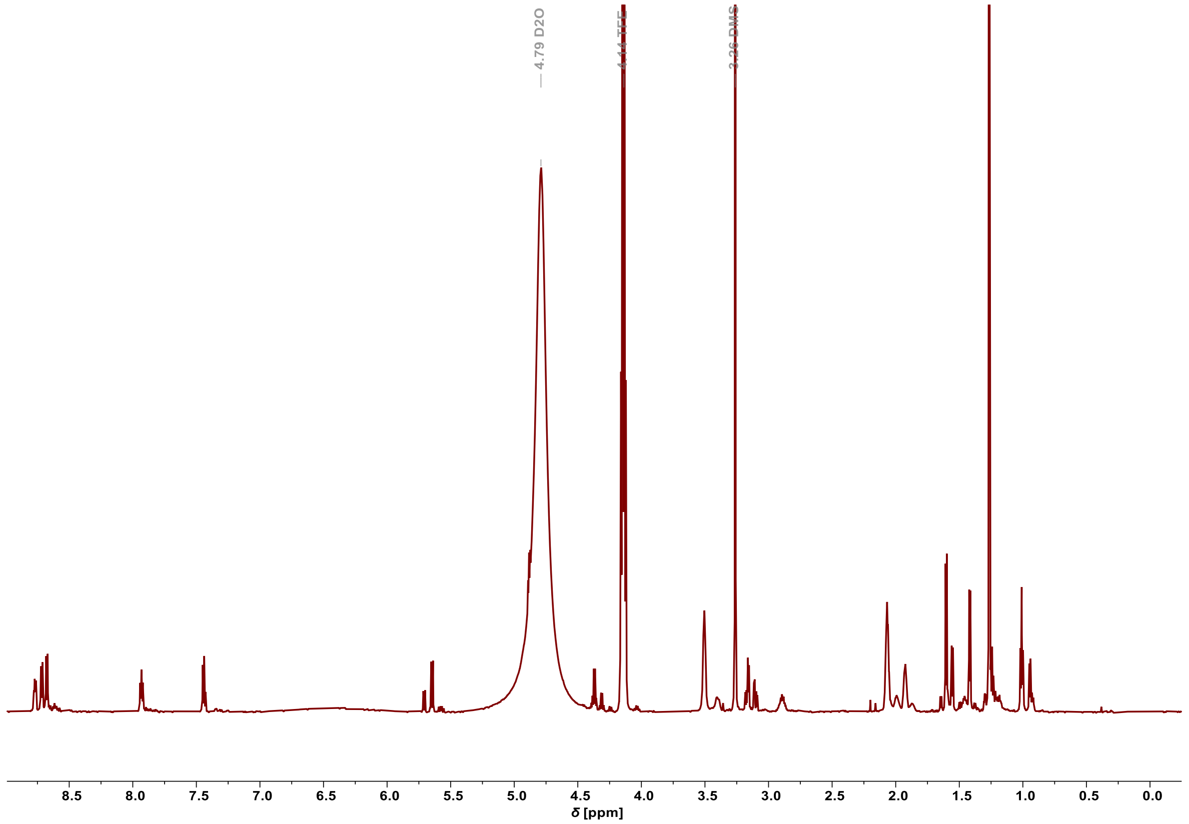

Figure S47: ^{1}H NMR (700 MHz, 298 K, D_2O/TFE-d_3 50:50) spectrum of C_0-Nap-ICA **8**.

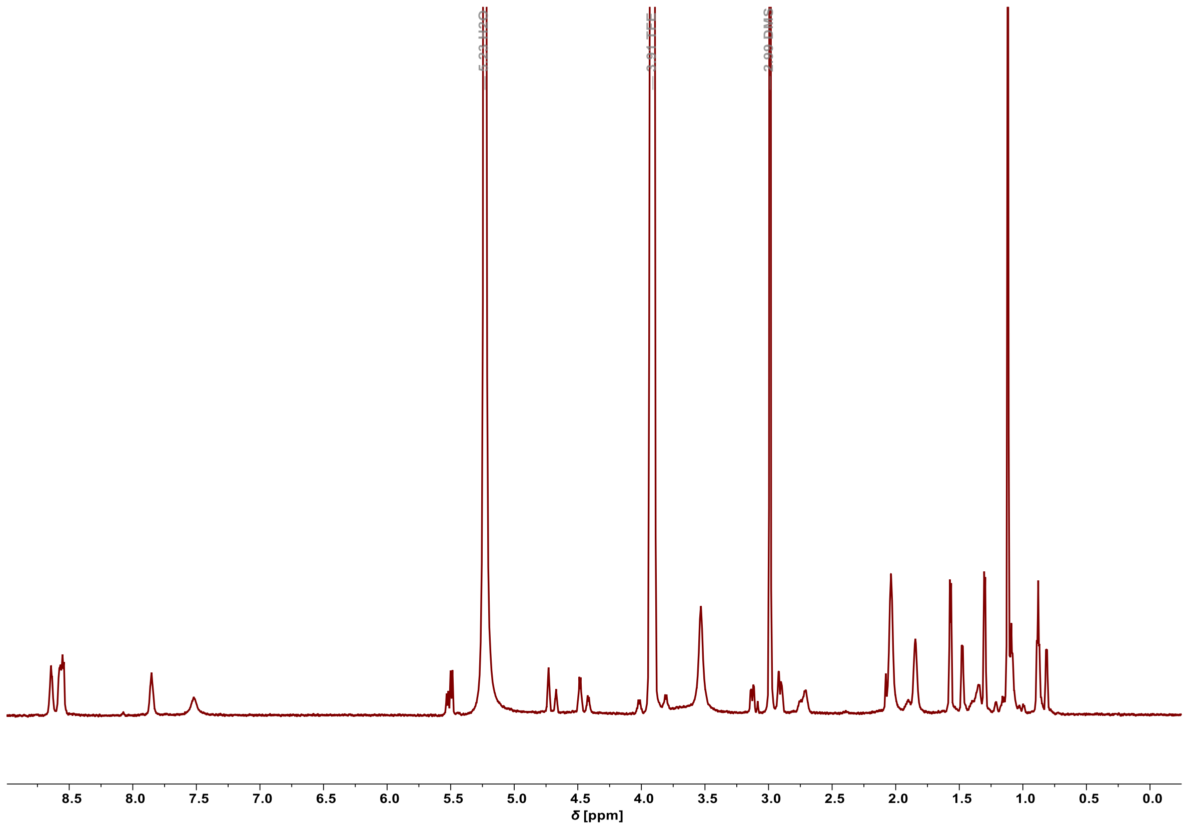

Figure S48: ^{1}H NMR (700 MHz, 298 K, TFE-d_3) spectrum of C_0-Nap-ICA **8**.

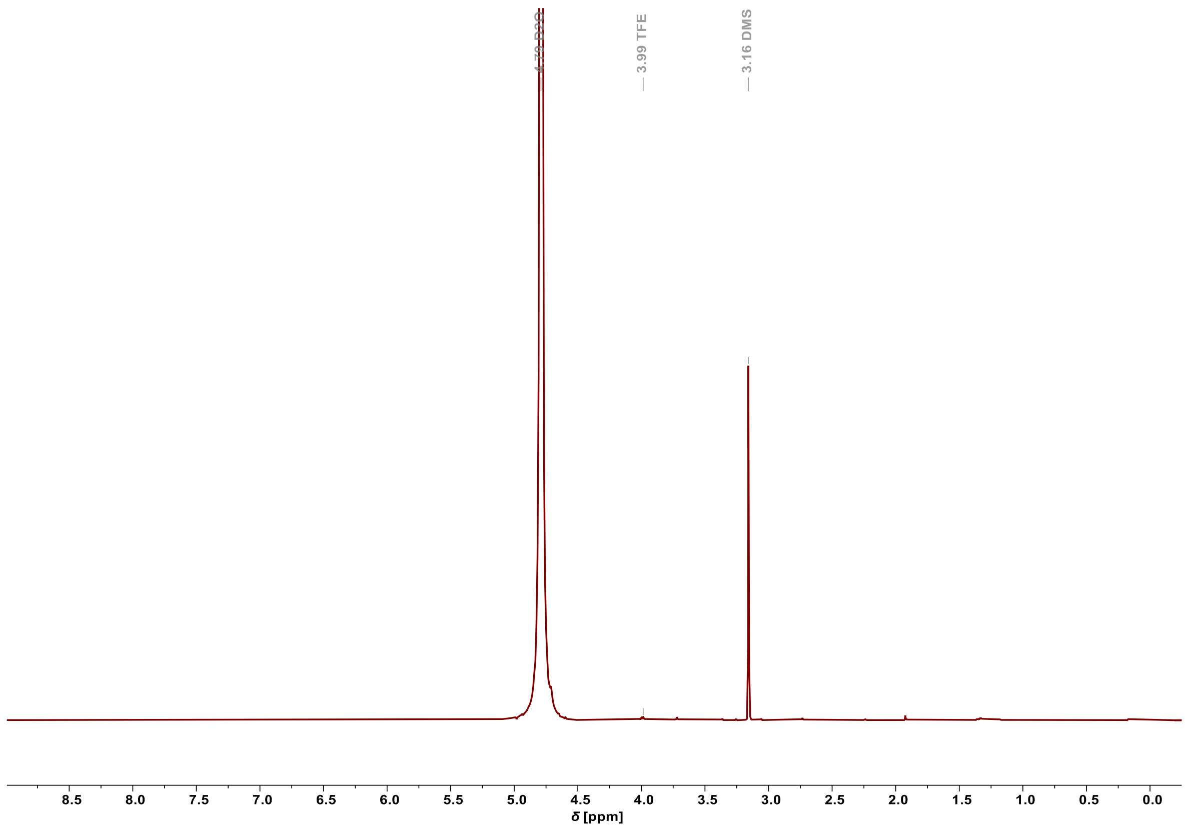

Figure S49: ^{1}H NMR (700 MHz, 298 K, D_2O/TFE-d_3 99:1) spectrum of C_3-Nap-ICA **7**.

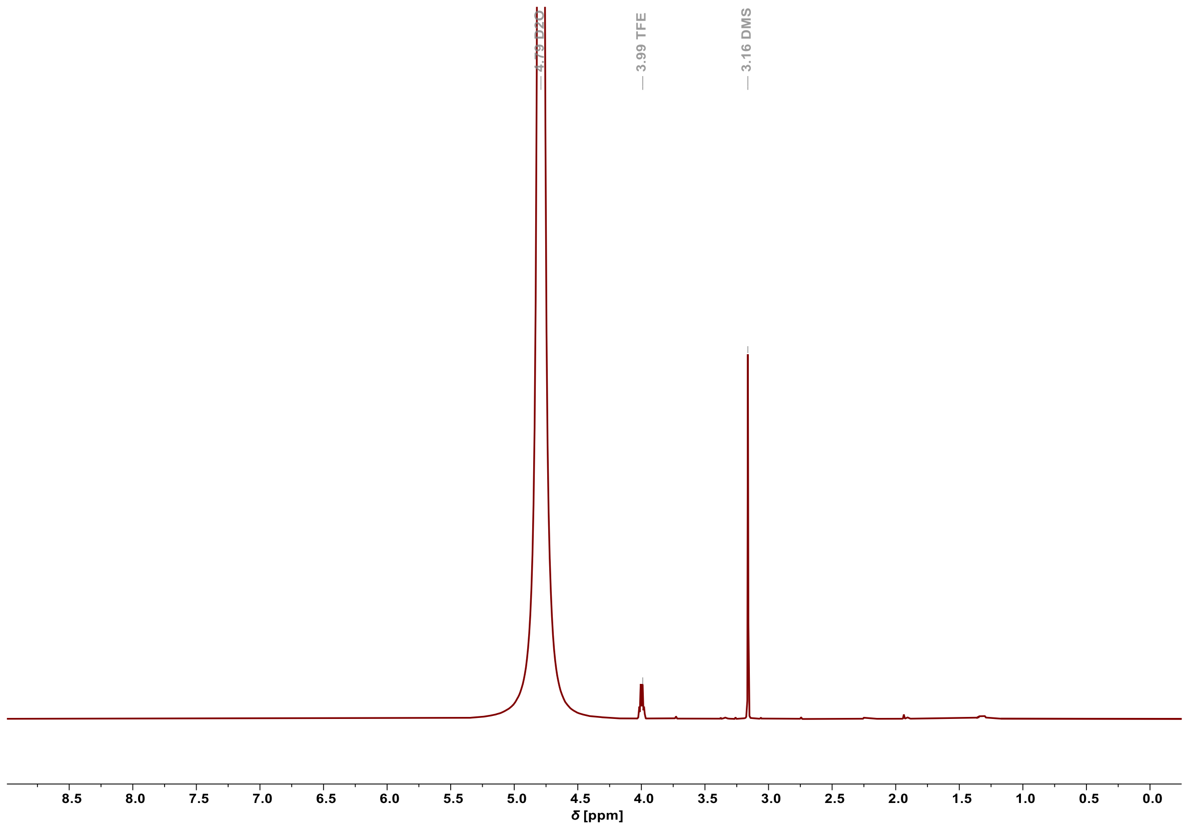

Figure S50: ^{1}H NMR (700 MHz, 298 K, D_2O/TFE-d_3 90:10) spectrum of C_3-Nap-ICA **7**.

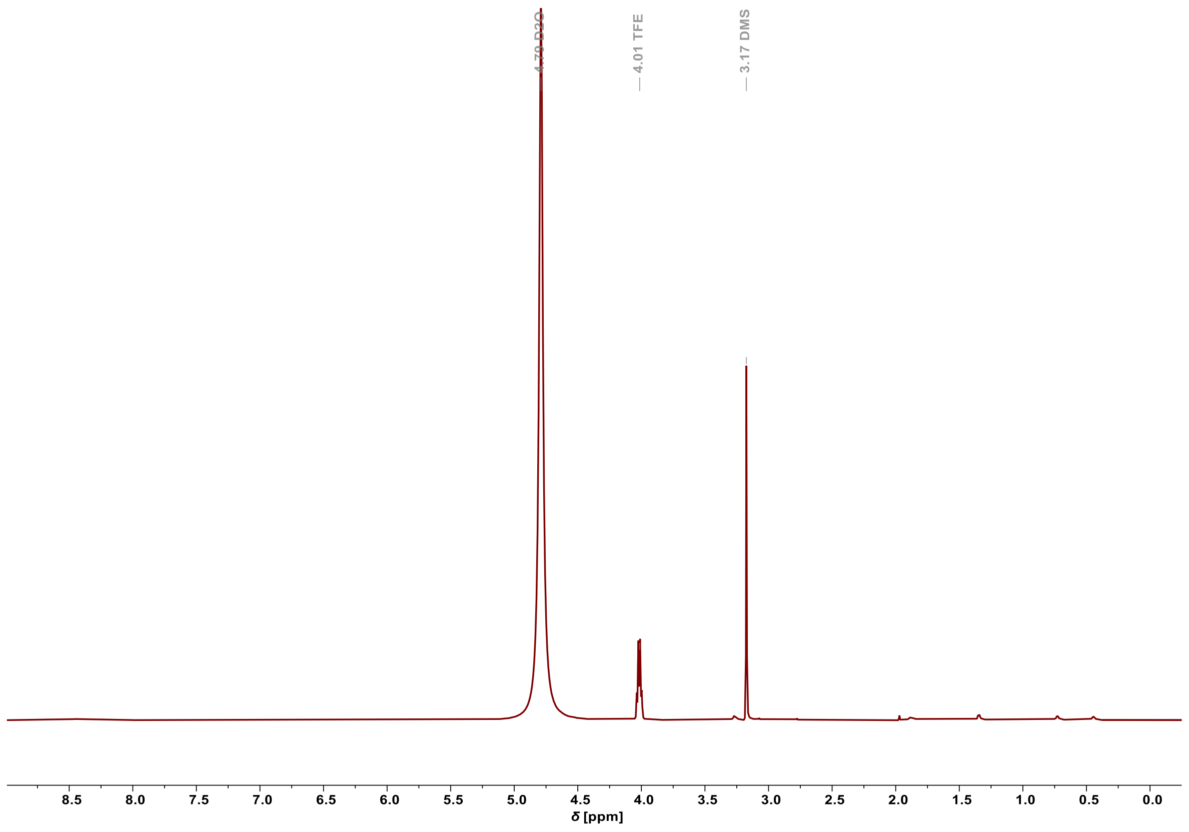

Figure S51: ^{1}H NMR (700 MHz, 298 K, D_2O/TFE-d_3 80:20) spectrum of C_3-Nap-ICA **7**.

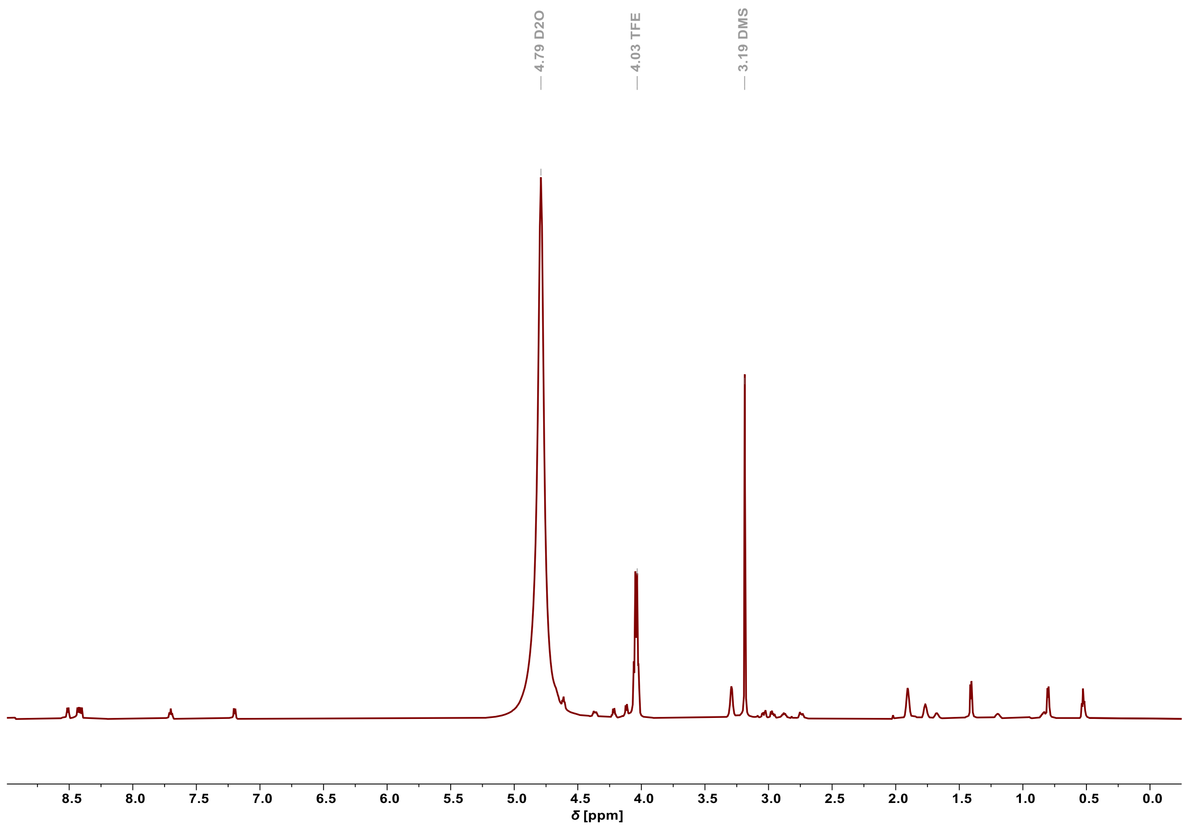

Figure S52: ^{1}H NMR (700 MHz, 298 K, D_2O/TFE-d_3 70:30) spectrum of C_3-Nap-ICA **7**.

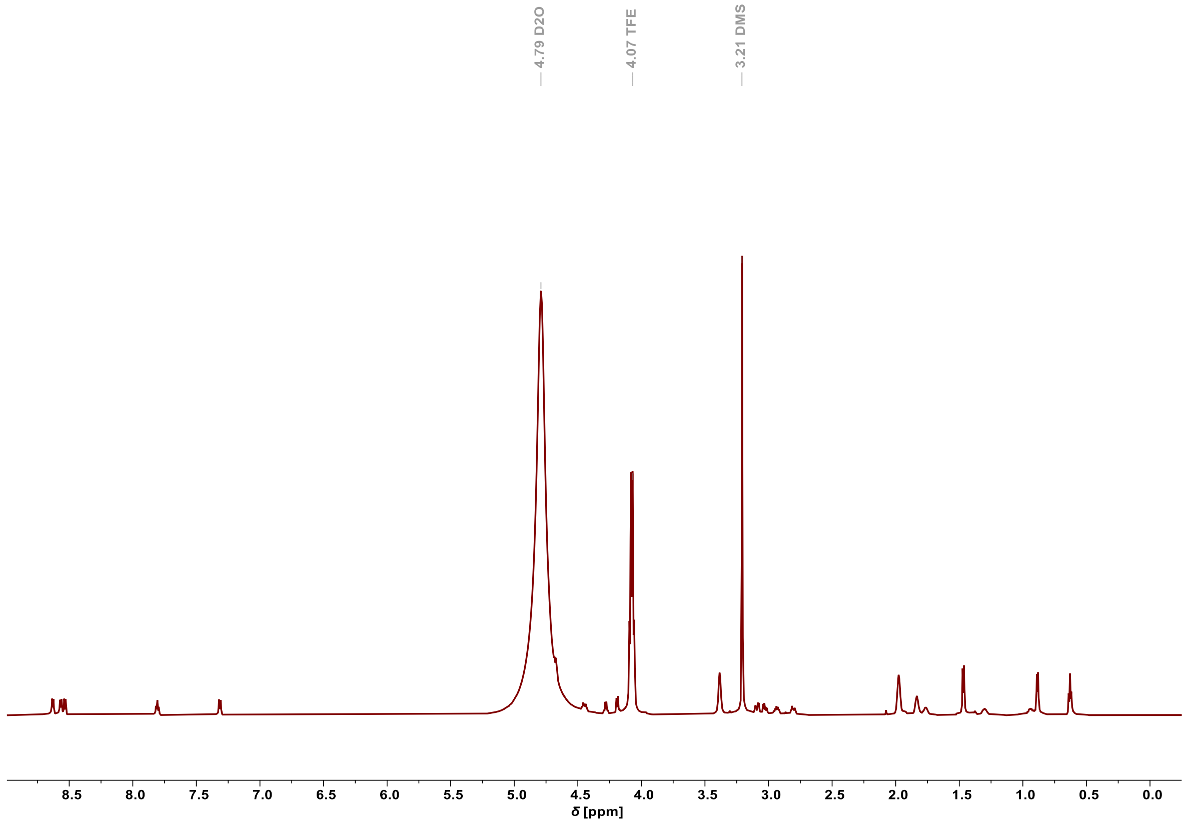

Figure S53: ^{1}H NMR (700 MHz, 298 K, D_2O/TFE-*d*$_3$ 60:40) spectrum of C_3-Nap-ICA **7**.

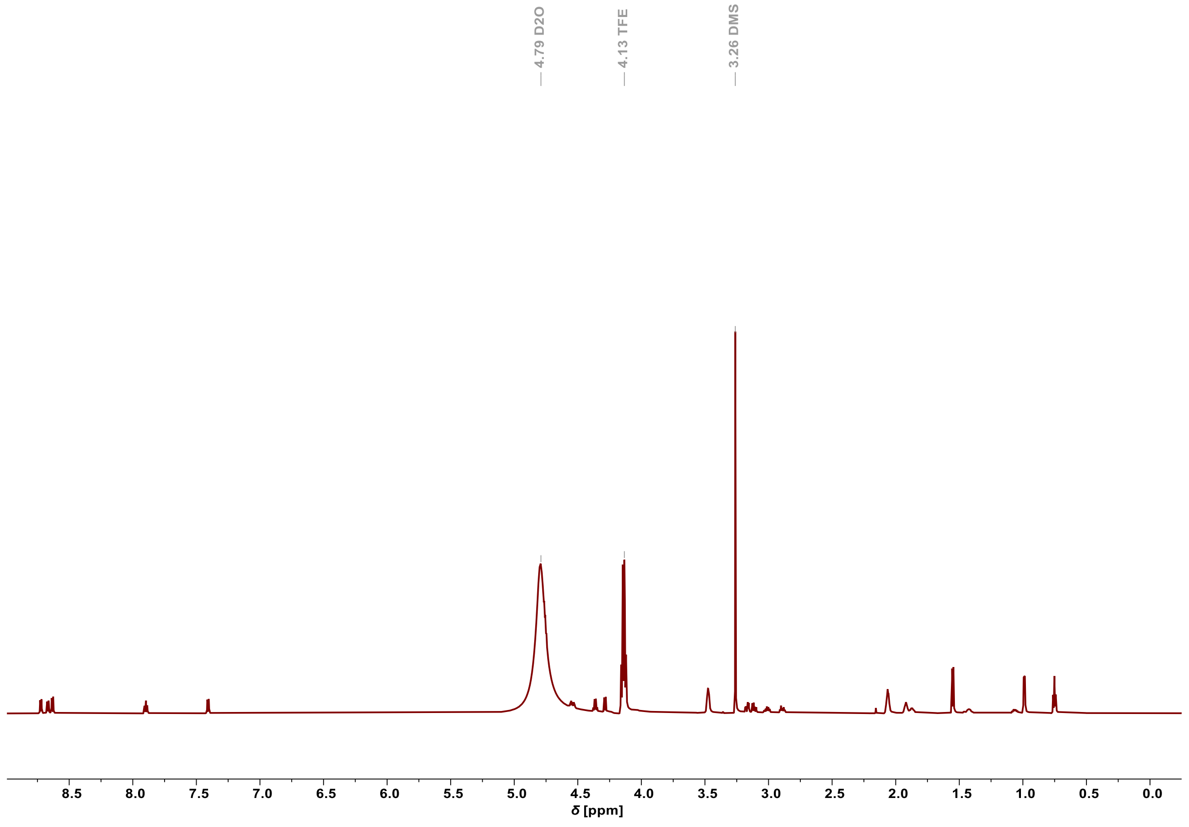

Figure S54: ^{1}H NMR (700 MHz, 298 K, D_2O/TFE-*d*$_3$ 50:50) spectrum of C_3-Nap-ICA **7**.

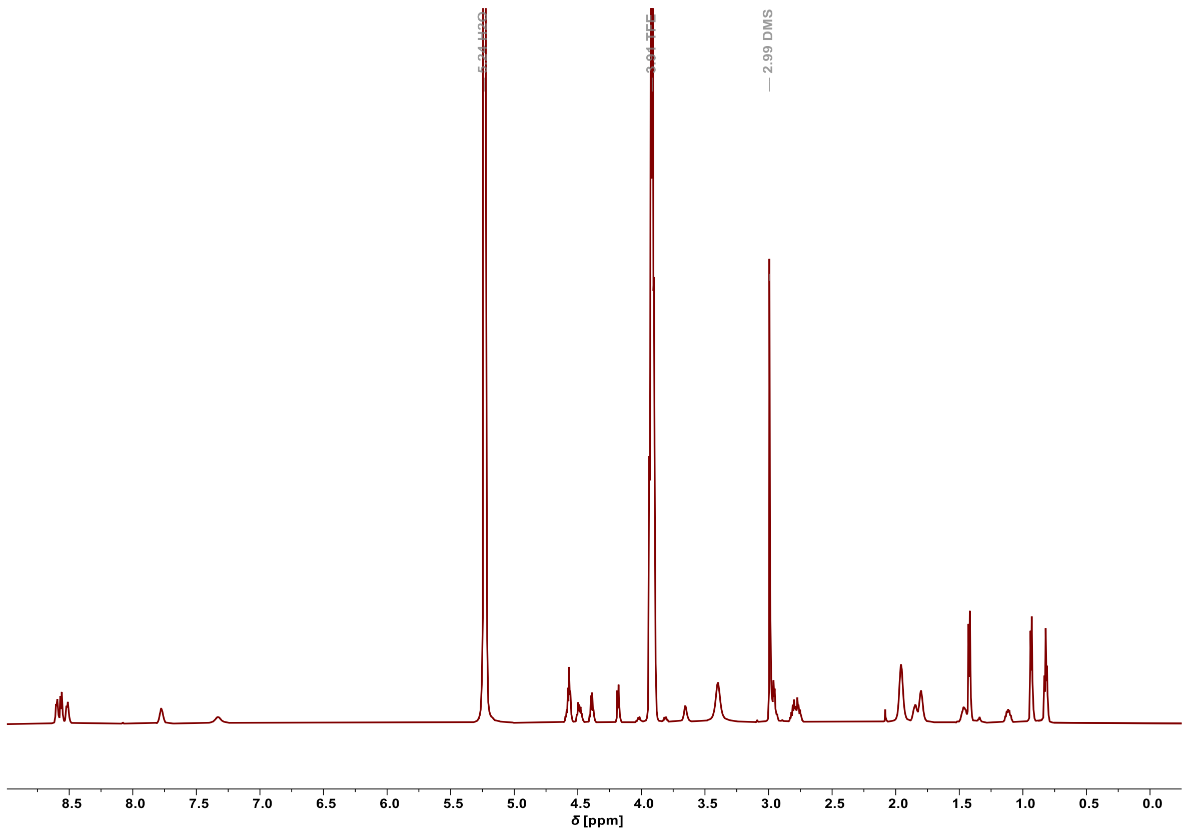

Figure S55: ^{1}H NMR (700 MHz, 298 K, TFE-d_3) spectrum of C_3-Nap-ICA **7**.

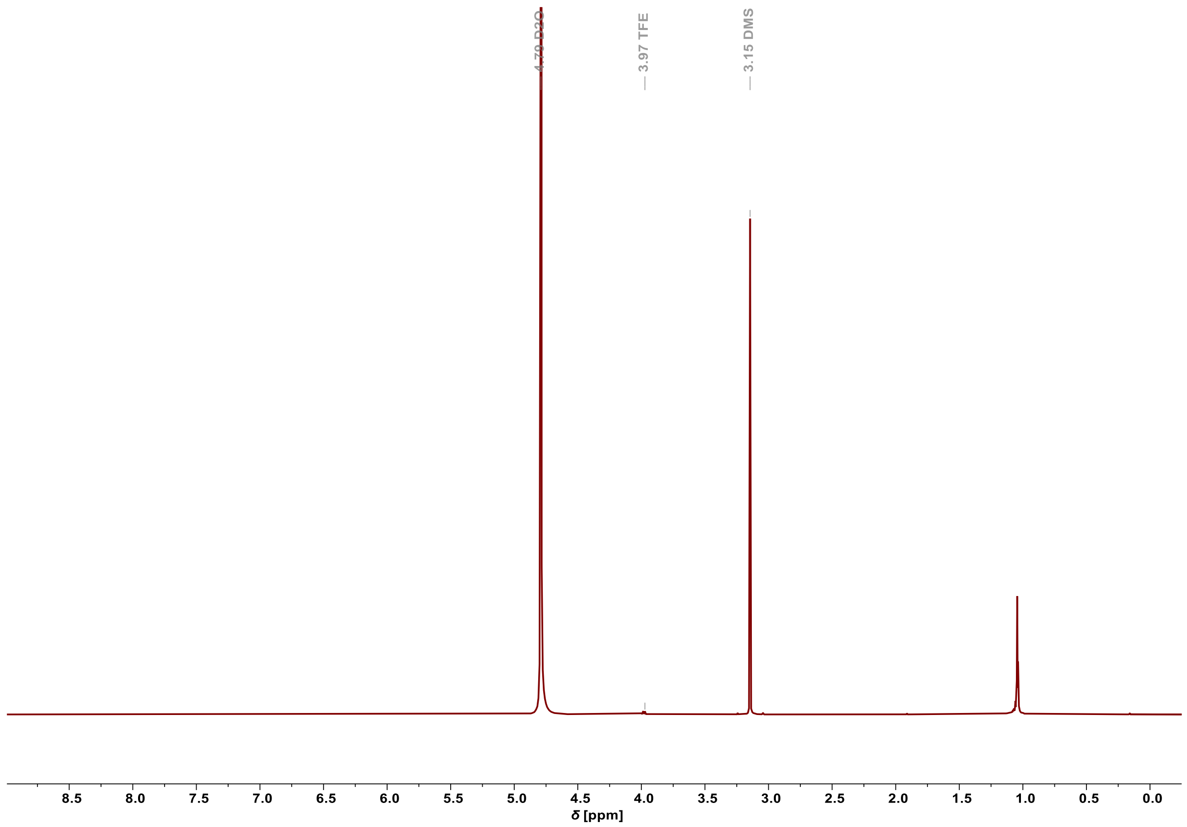

Figure S56: ^{1}H NMR (700 MHz, 298 K, D_2O/TFE-d_3 99:1) spectrum of C_6-Nap-ICA **6**.

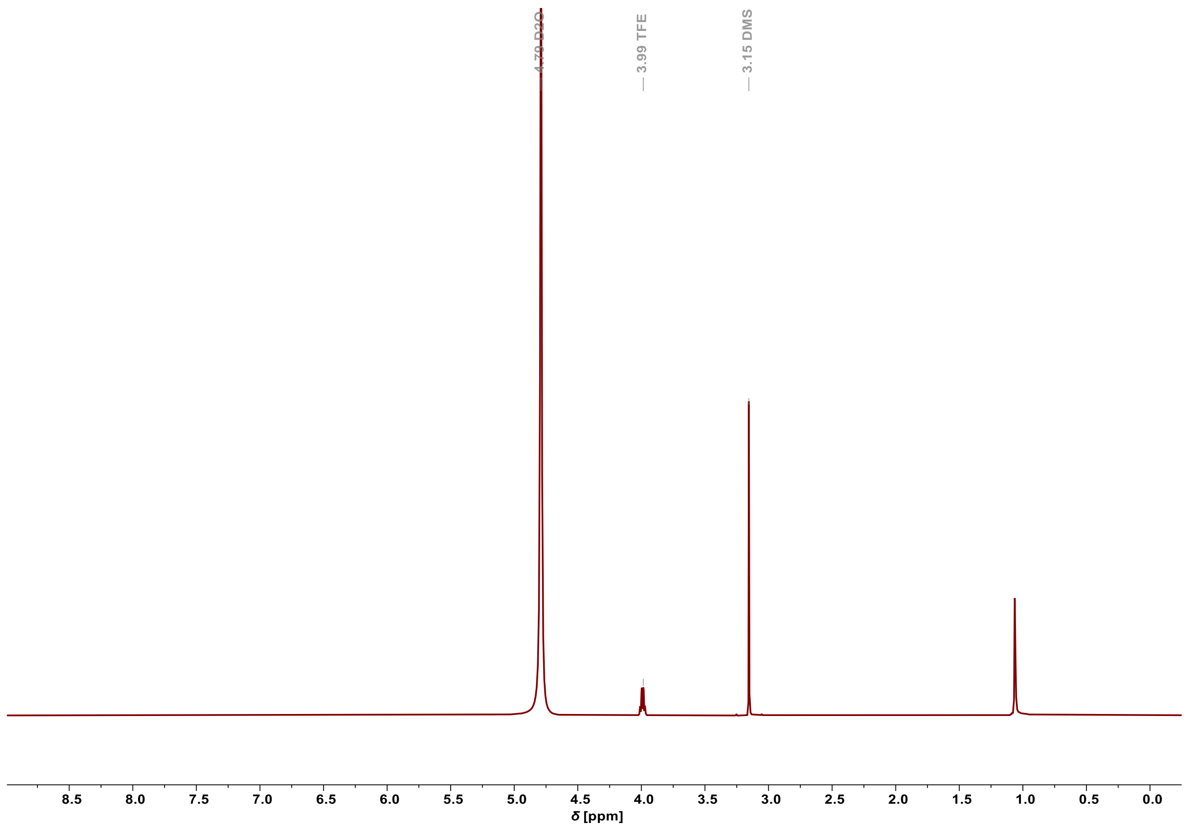

Figure S57: ^{1}H NMR (700 MHz, 298 K, D_2O/TFE-d_3 90:10) spectrum of C_6-Nap-ICA **6**.

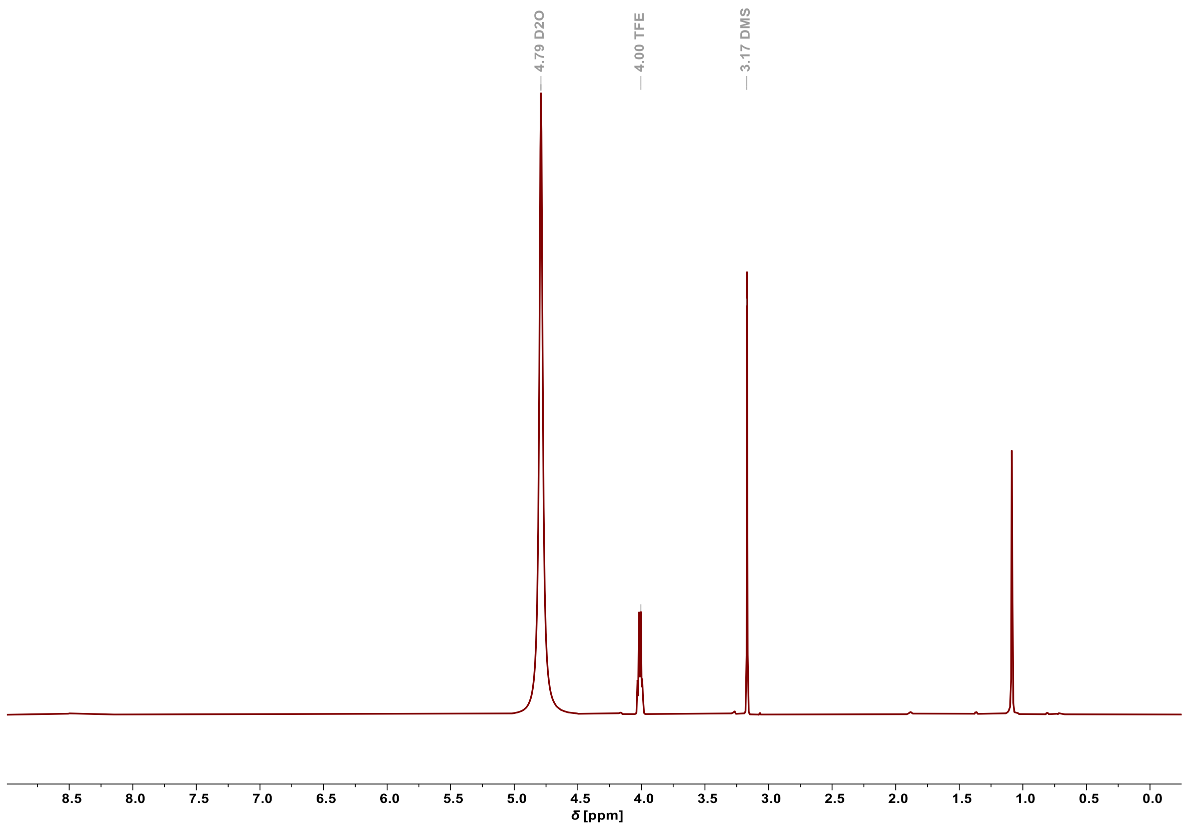

Figure S58: ^{1}H NMR (700 MHz, 298 K, D_2O/TFE-d_3 80:20) spectrum of C_6-Nap-ICA **6**.

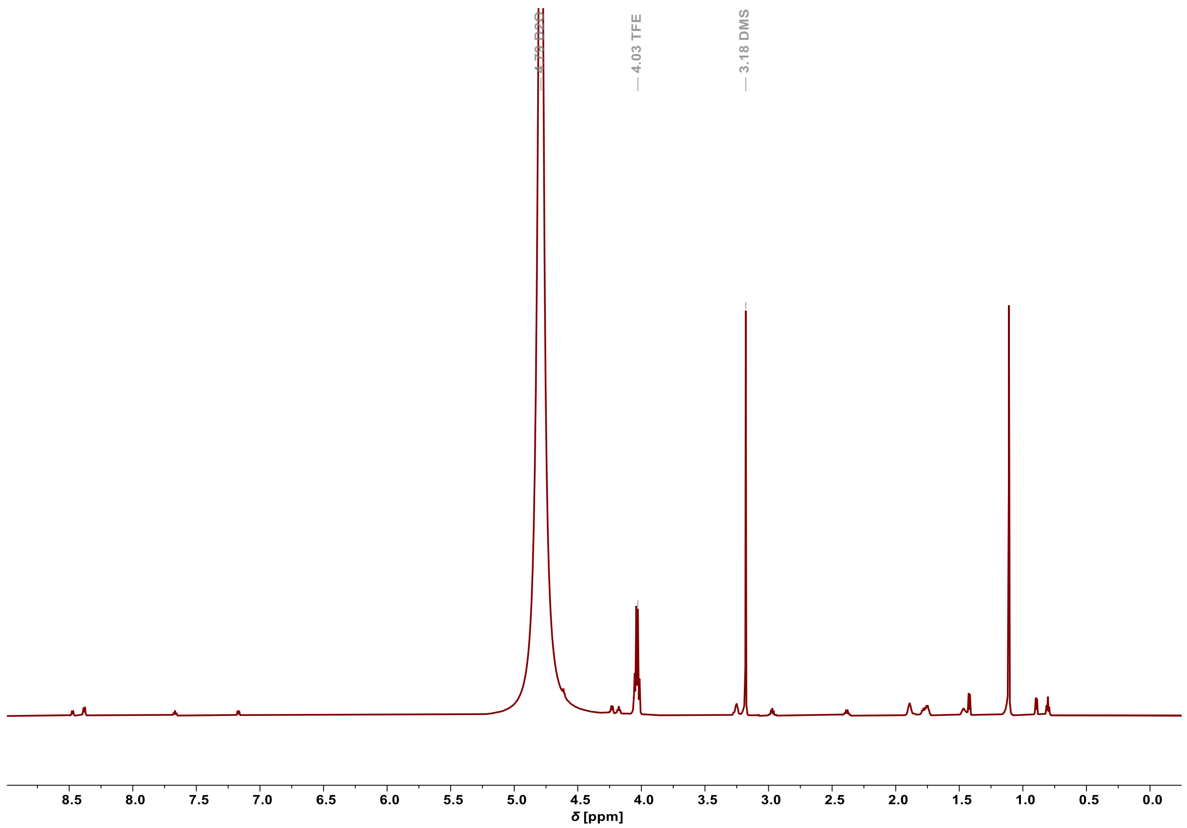

Figure S59: ^{1}H NMR (700 MHz, 298 K, D_2O/TFE-d_3 70:30) spectrum of C_6-Nap-ICA **6**.

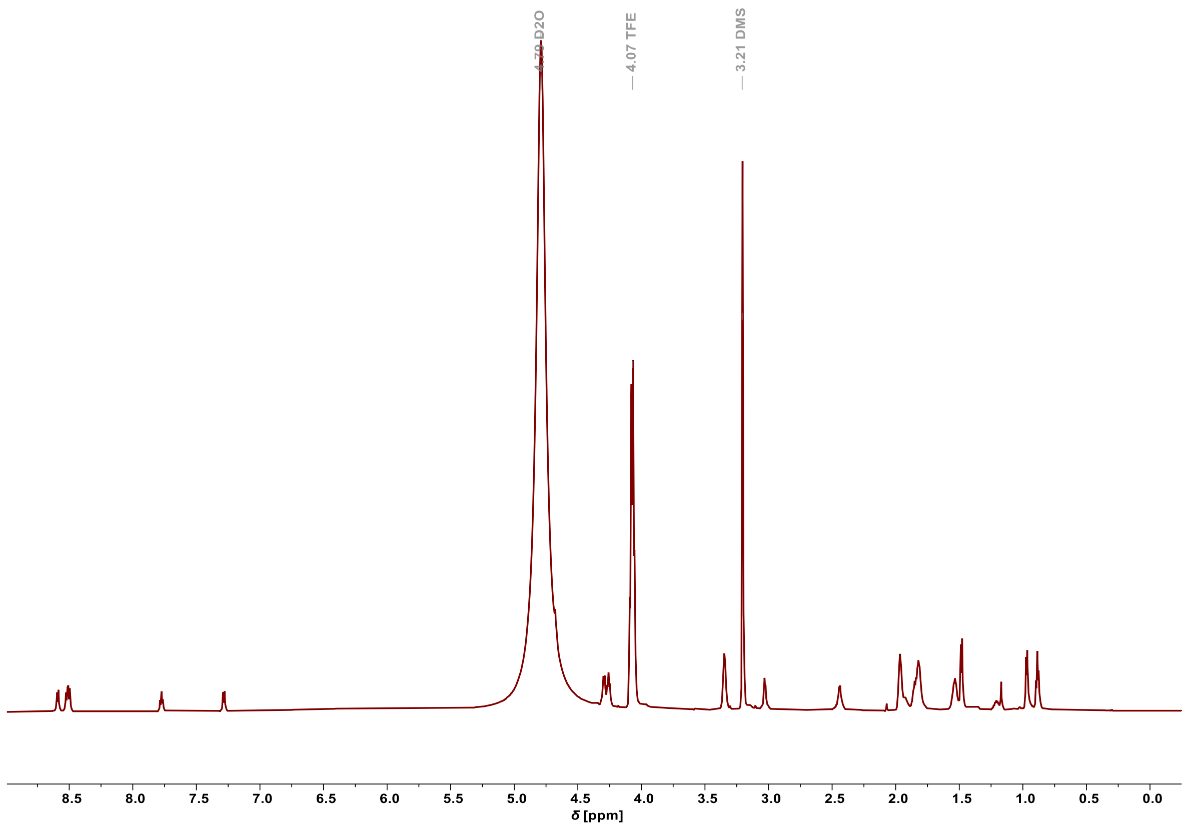

Figure S60: ^{1}H NMR (700 MHz, 298 K, D_2O/TFE-d_3 60:40) spectrum of C_6-Nap-ICA **6**.

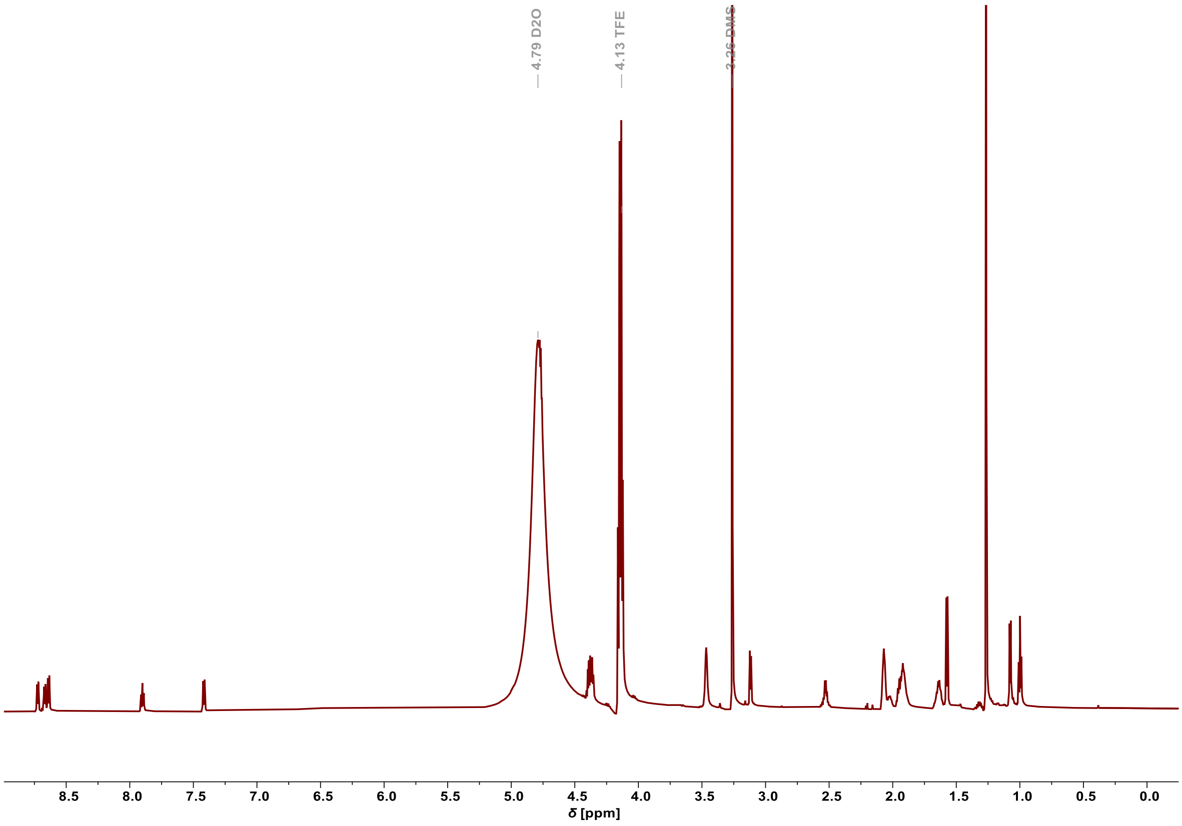

Figure S61: ^{1}H NMR (700 MHz, 298 K, D_2O/TFE-d_3 50:50) spectrum of C_6-Nap-ICA **6**.

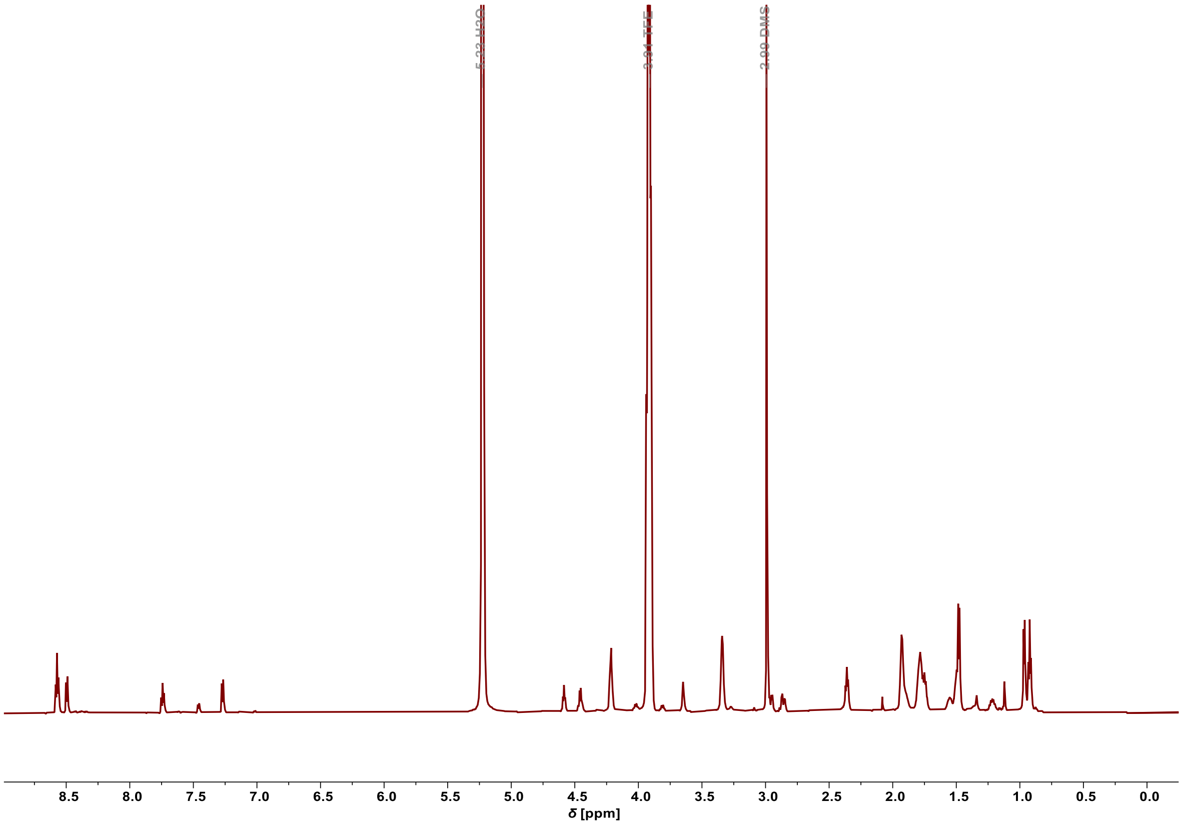

Figure S62: ^{1}H NMR (700 MHz, 298 K, TFE-d_3) spectrum of C_6-Nap-ICA **6**.

Table S1: Masses and amounts of peptides and masses of solvents for NMR experiment with increasing TFE-d_3 contents

Compound and % TFE	*m* [ug]	*n* [µmol]	m_{TFE} [mg]	$m_{DMS\text{-}buffer}$ [mg]	$m_{noDMS\text{-}buffer}$ [mg]
C_0-Nap-ICA **8** 1%	497	0.874	6.3	275.3	273.3
C_0-Nap-ICA **8** 10%	493	0.867	66.2	276.2	217.8
C_0-Nap-ICA **8** 20%	500	0.879	132.5	275.9	162.5
C_0-Nap-ICA **8** 30%	466	0.819	198.4	277.7	110.8
C_0-Nap-ICA **8** 40%	512	0.900	267.3	277.6	53.4
C_0-Nap-ICA **8** 50%	504	0.886	350.0	276.0	0
C_0-Nap-ICA **8** 100%	508	0.893	702.3	0	0
C_3-Nap-ICA **7** 1%	482	0.753	6.3	277.1	264.8
C_3-Nap-ICA **7** 10%	475	0.742	65.9	276.1	217.1
C_3-Nap-ICA **7** 20%	460	0.719	132.7	275.9	164.7
C_3-Nap-ICA **7** 30%	466	0.728	198.3	276.1	110.2
C_3-Nap-ICA **7** 40%	468	0.731	264.5	276.3	55.3
C_3-Nap-ICA **7** 50%	488	0.763	329.8	277.7	0
C_3-Nap-ICA **7** 100%	528	0.825	711.4	0	0
C_6-Nap-ICA **6** 1%	514	0.754	6.1	275.1	272.1
C_6-Nap-ICA **6** 10%	524	0.769	62.9	277.0	198.8
C_6-Nap-ICA **6** 20%	534	0.784	133.0	277.7	163.0
C_6-Nap-ICA **6** 30%	528	0.775	199.3	278.6	110.0
C_6-Nap-ICA **6** 40%	480	0.705	264.8	278.0	53.4
C_6-Nap-ICA **6** 50%	536	0.787	353.7	274.1	0
C_6-Nap-ICA **6** 100%	507	0.744	708.9	0	0

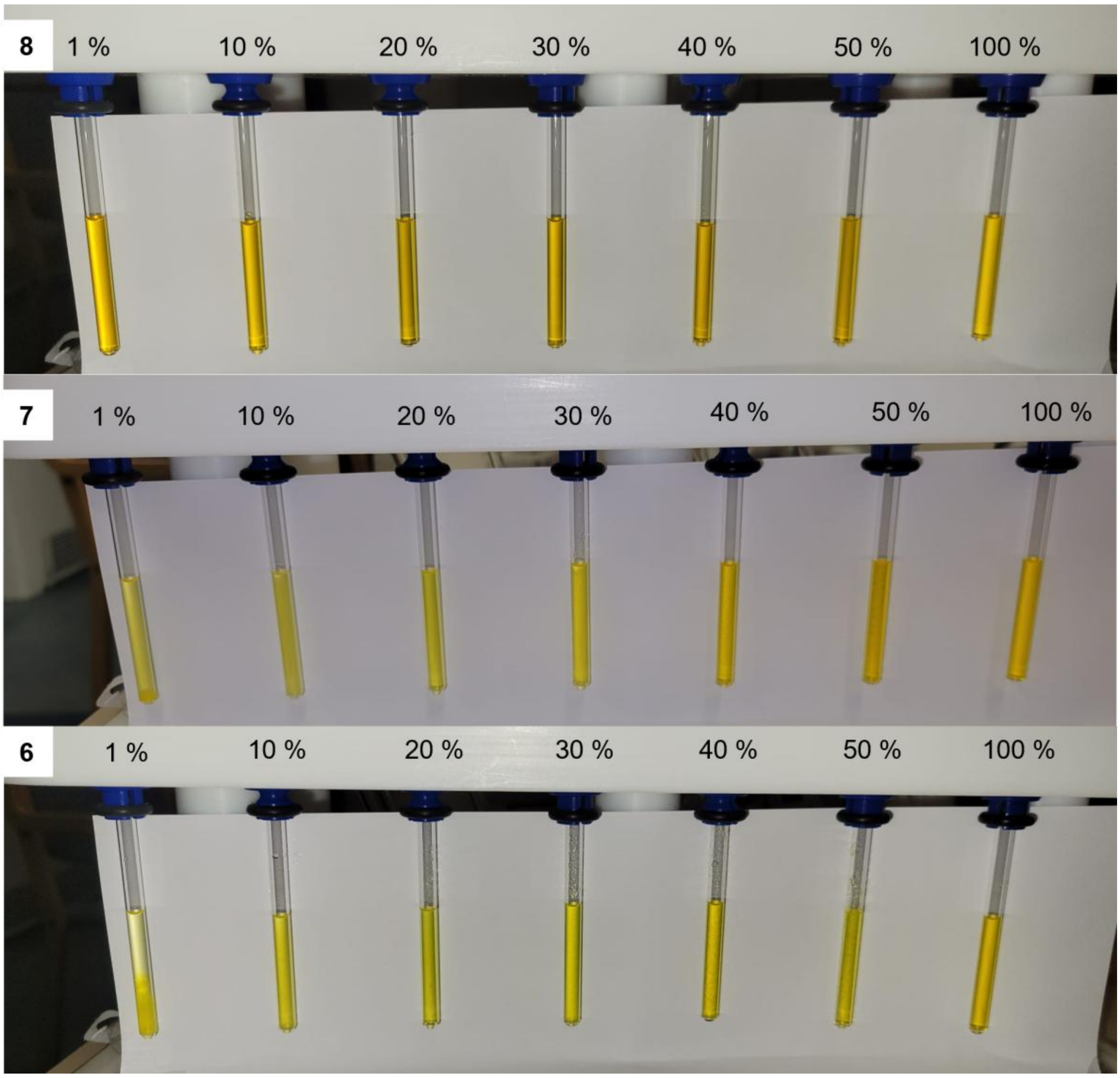

Figure S63: Photographs of the respective NMR tubes of the three peptide conjugates C_0-Nap-ICA **8** (top), C_3-Nap-ICA **7** (middle) and C_6-Nap-ICA **6** (bottom) with different TFE-d_3 contents.

References

[1] Atherton, E.; Fox, H.; Harkiss, D.; Logan, C. J.; Sheppard, R. C.; Williams, B. J. *Journal of the Chemical Society, Chemical Communications* **1978**, No. 13, 537–539. doi:10.1039/C39780000537

[2] Li, L.; Wu, Y.; Li, E.; Shen, C.; Zhang, H.; Xu, X.; Wu, G.; Cai, M.; Zhu, W.-H. *Chemical Communications* **2019**, *55*, 13239–13242. doi:10.1039/C9CC06345E

[3] Mata, M. A.; Satterly, N.; Versteeg, G. A.; Frantz, D.; Wei, S.; Williams, N.; Schmolke, M.; Peña-Llopis, S.; Brugarolas, J.; Forst, C. V.; White, M. A.; García-Sastre, A.; Roth, M. G.; Fontoura, B. M. A. *Nature Chemical Biology* **2011**, *7*, 712–719. doi:10.1038/nchembio.645
